# Transmetalation Reactions Triggered by Electron Transfer between Organocopper Complexes

**DOI:** 10.1021/acs.inorgchem.1c01595

**Published:** 2021-07-14

**Authors:** Olmo Lozano-Lavilla, Pablo Gómez-Orellana, Agustí Lledós, Juan A. Casares

**Affiliations:** & IU CINQUIMA/Química Inorgánica, Facultad de Ciencias, Universidad de Valladolid, Valladolid 47011, Spain; § Departament de Química, Edifici C.n. Universitat Autònoma de Barcelona, Cerdanyola del Vallès, Catalonia 08193, Spain

## Abstract

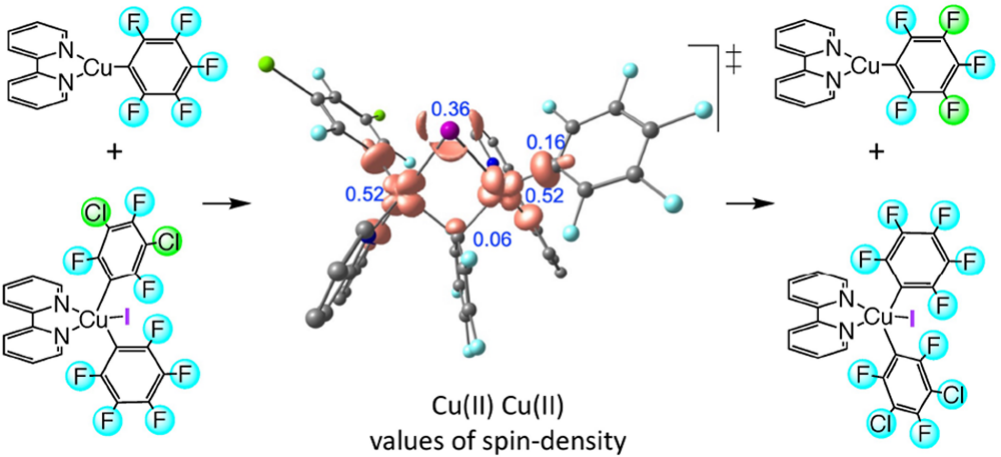

[Cu(bipy)(C_6_F_5_)] reacts with most aryl iodides to form heterobiphenyls by cross-coupling, but when Rf–I is used (Rf = 3,5-dicholoro-2,4,6-trifluorophenyl), homocoupling products are also formed. Kinetic studies suggest that, for the homocoupling reaction, a mechanism based on transmetalation from [Cu(bipy)(C_6_F_5_)] to Cu(III) intermediates formed in the oxidative addition step is at work. Density functional theory calculations show that the interaction between these Cu(III) species and the starting Cu(I) complex involves a Cu(I)–Cu(III) electron transfer concerted with the formation of an iodine bridge between the metals and that a fast transmetalation takes place in a dimer in a triplet state between two Cu(II) units.

## Abstract 2

In copper-catalyzed cross-coupling reactions, electron-transfer processes between Cu(I) and Cu(III) species are overlooked behind RDS (C−X activation). Density functional theory studies considering two molecules of the catalyst and two spin states throughout the course of the reaction have revealed the feasibility of such a process and the transmetalation between Cu(II) species, justifying in this way the formation of homocoupling products.

## Introduction

Copper occupies a prominent place among the metals used in organic synthesis, and its compounds are extremely versatile and efficient homogeneous catalysts. The Ullmann reaction for the homocoupling of arenes using metallic copper as the catalyst was reported more than a century ago, and during this time milder conditions have been developed by using copper complexes instead of the bulk metal.^1,2^ Simultaneously, methodologies for cross-coupling based on copper have been developed and complement palladium and nickel catalysts.^3−6^

**Scheme 1:**
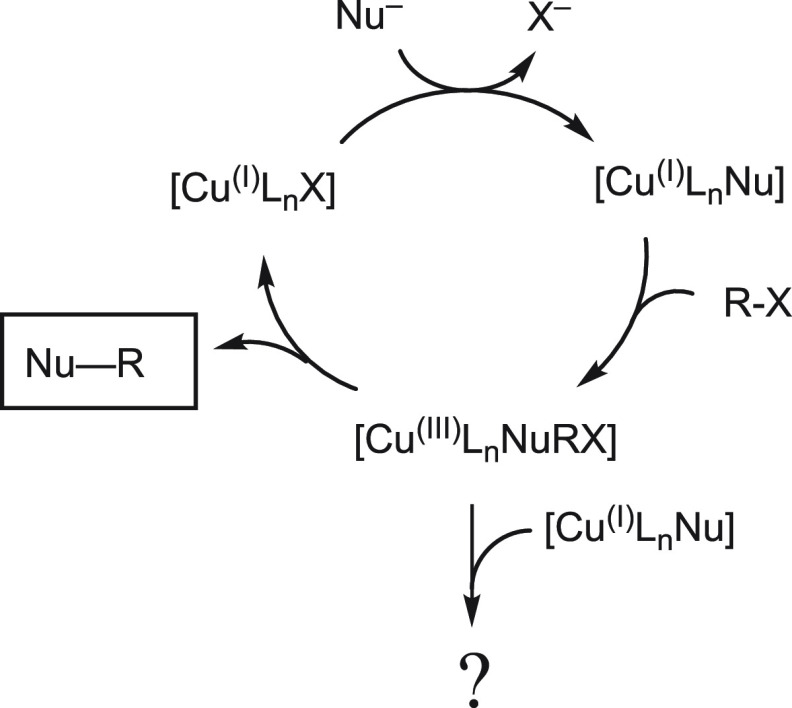
Accepted Catalytic Cycle for Copper-Catalyzed Cross-Coupling Reactions and the Hypothetical Interaction between Cu(III) and Cu(I) Species

Quite surprisingly, it is difficult to correlate mechanistically both reactions, leading to homocoupling and cross-coupling. By analogy with palladium, the most accepted mechanistic proposal for copper-catalyzed cross-coupling reactions includes the oxidative addition of R–X to a Cu(I), forming a Cu(III) intermediate in which reductive elimination takes place (Scheme 1). However, mechanisms involving radical oxidation to produce Cu(II) intermediates seem to operate in a significant number of reactions, and Cu(II) intermediates have been invoked in several mechanistic proposals for the Ullmann reaction.^2,7,8^

The two-electron oxidative addition reaction has been addressed in computational and experimental studies.^2,9−19^ It entails an oxidation state change between Cu(I) (the resting state of the catalyst) and Cu(III). This is usually the slowest step on the cycle, and unlike what happens in palladium-catalyzed reactions, the oxidized state, Cu(III), is often considered to be unstable and therefore a very reactive intermediate.^20^ Hence, the reaction between the Cu(I) species, very abundant in the reaction medium, and the very reactive Cu(III) intermediates cannot be disregarded in these systems.

Surprisingly, the interaction between Cu(III) and Cu(I) species remains almost unexplored, although the reversal process involving the disproportionation of Cu(II) organometallics has been proposed as a critical step in several catalytic systems.^21,22^ Also, even though transmetalation between copper species is implicitly assumed in several coupling processes and connects the Ullmann homocoupling reaction with the cross-coupling reaction, transmetalation between Cu(I) complexes and other copper species has not been explicitly considered in experimental- or computational-based mechanistic proposals. This is striking considering the known ability of Cu(I) organometallics to transmetalate to other metals, particularly d^8^ complexes such as Pd(II) derivatives.^23,24^ This lack of fundamental chemical information prompted us to address the study of these reactions.

## Results and Discussion

### Kinetic Studies

**Scheme 2:**
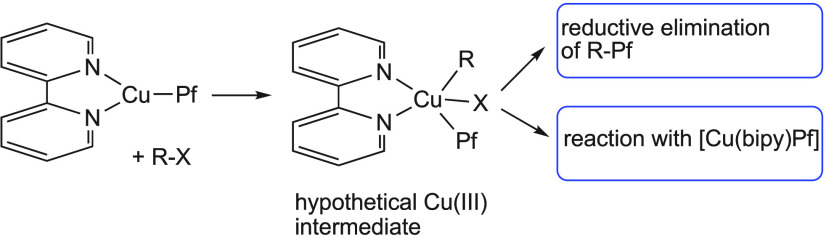
Expected Pathways for Cu(III) Intermediates Formed by Oxidative Addition

We have pursued to induce the interaction between Cu(I) and Cu(III) complexes in transmetalation reactions between [Cu(bipy)(Pf)] (**1**; Pf = C_6_F_5_) and the putative Cu(III) intermediates formed after the oxidative addition of aryl iodides to **1**. If the reductive elimination takes place very fast, the cross-coupling product is obtained; otherwise, the Cu(III) intermediate could be intercepted by reacting with Cu(I) species (Scheme 2).

**Figure 1:**
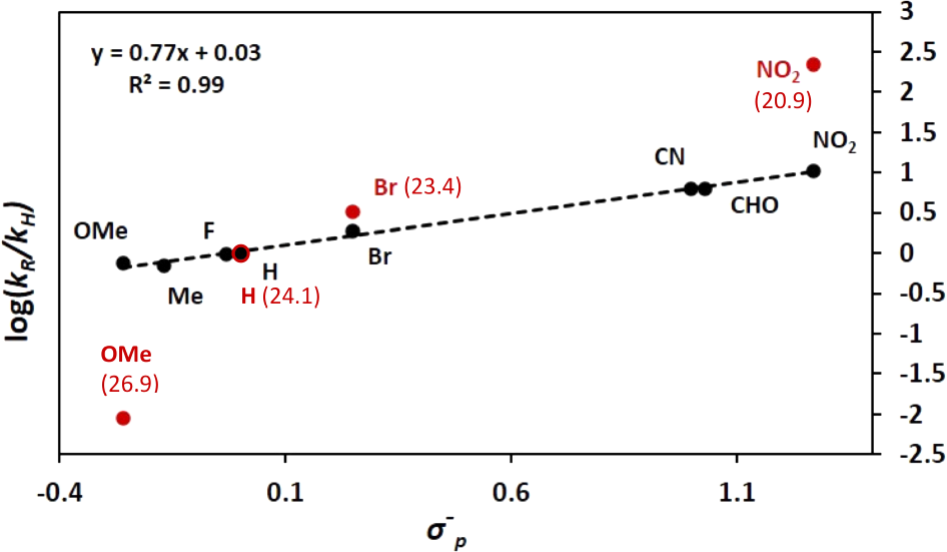
Hammett plot for the reaction rates of the pentafluorophenylation reaction of aryl iodides with the para substituents indicated in the figure. The numbers in red are the computed Gibbs energy barriers in THF, in kcal mol^–1^.

The reactions of **1** with nonfluorinated aryl iodides R–I follow only the first path, giving the cross-coupling products R–Pf without any detectable intermediate and without homocoupling products other than those formed by the basal decomposition of **1**. The kinetics show a first-order dependence on the copper complex. To explore the influence of the electronic properties of the aryl halides in this reaction, competitive experiments with different para-substituted aryl iodides along with iodobenzene were carried out. For the reaction with Ph–I at 50 °C, *k*_obs_ = 2.14 × 10^–4^ s^–1^ M^–1^ and Δ*G*^⧧^_318.16_ = 24 kcal mol^–1^.^25^ The larger relative rates were obtained for the aryl iodides bearing the most electron-withdrawing substituents, and the Hammett plots of the relative reaction rates displayed a very good correlation with the σ_p_^–^ values of the para substituents (Figure 1). The density functional theory (DFT)-computed barriers reproduce this tendency (red numbers in Figure 1). These results suggest that the reaction behaves as a nucleophilic attack of the copper complex to the *ipso*-carbon of the aryl iodide, in accordance with previous studies in other Cu(I) systems.^12,26^ The trend of computed atomic charges at the *ipso*-carbon agrees with this view (see the Supporting Information, SI).

As reported for analogous systems, **1** suffers a self-ionization equilibrium in solution to give the cation [Cu(bipy)_2_]^+^ and the cuprate [Cu(Pf)_2_]^−^ (eq 1).^12,27,28^
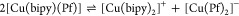
1From a van’t Hoff plot, the values Δ*H* = −2.7 ± 0.3 kcal mol^–1^ and Δ*S* = 23 ± 2 cal mol^–1^ K^–1^ were obtained for this reaction. Using these parameters, *K*_eq_ at 25 °C takes a value of 9 × 10^–4^, meaning that about 97% of the copper in the system is in the form of the neutral complex **1**. The participation of cuprates in the oxidative addition reaction has been previously discussed, both computationally and experimentally, and in a very general way, it has been found that they react at a much lower rate than the parent neutral tricoordinated complexes.^12,29^ To verify this fact in our system, the tetrabutylammonium salt of the organocuprate (NBu_4_)[CuPf_2_] was synthesized (see the synthesis and X-ray structure in the SI), and the reaction of pentafluorophenylation of iodobenzene was attempted in the same conditions as those for compound **1**. Only traces of the heterocoupling product were obtained from this experiment. The same result was obtained by using [Cu(bipy)_2_]^+^ or [Cu(bipy)I] as the copper reagent.^14^

Thus, in principle, the cross-coupling can be pictured as an oxidative addition initiated by a nucleophilic attack of complex **1** on the C–I bond to give an intermediate of the type [Cu(bipy)I(Pf)(Ar)], followed by fast reductive elimination.^30^

**Scheme 3:**
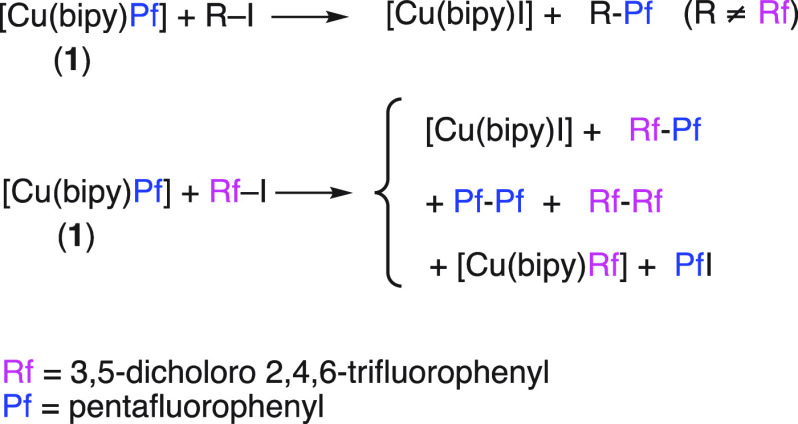
Products Obtained from the Reaction of Complex **1** with Aryl Iodides See the SI for reaction conditions and yields.

In an attempt to stabilize the Cu(III) species after the nucleophilic attack step, we tested the oxidative addition of 1-iodo-3,5-dichloro-2,4,6-trifluorobenzene (Rf–I). The reaction would lead to a Cu(III) intermediate with two different fluoroaryl substituents that would benefit from the large stabilization of the C–M bonds that fluoroaryls provide.^31^ The presumed intermediate was not stable enough to be spectroscopically observed. Instead, in addition to the expected cross-coupling product Rf–Pf, the homocoupling biaryls Pf–Pf and Rf–Rf were formed along with [Cu(bipy)(Rf)] and Pf–I (Scheme 3).

**Scheme 4:**
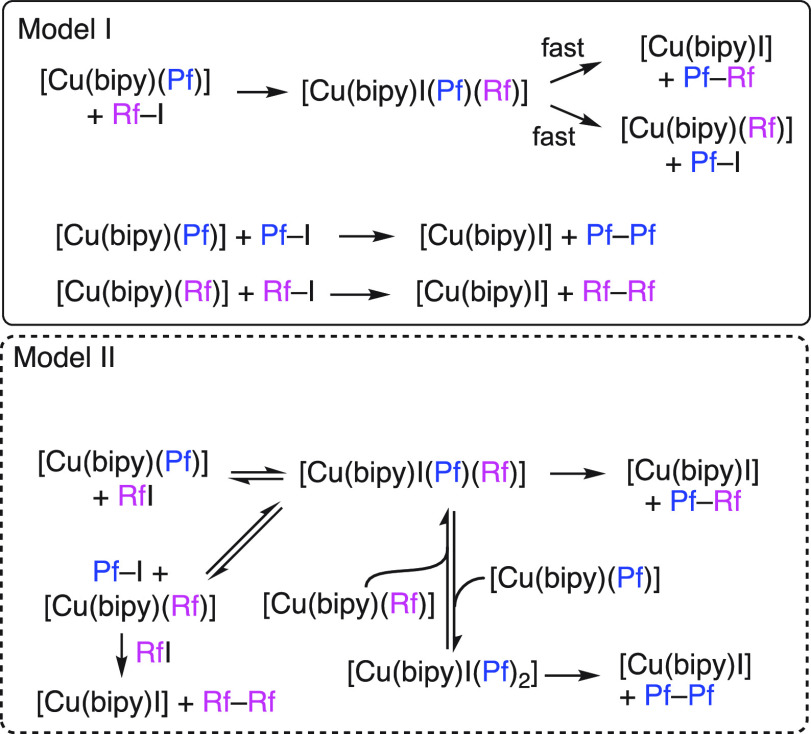
Simplified Kinetic Models See the SI for a more detailed description. In model I, the homocoupling product Pf–Pf is formed after accumulation in the medium of Pf–I, which can then react with Cu(I) complexes.

Two pathways can be envisioned to explain the formation of homocoupling products: a set of double oxidative addition–fast reductive elimination reactions (model I in Scheme 4) or a pathway involving Cu(III) intermediates that react with **1** to form species containing two Pf groups (model II in Scheme 4).^32^

**Figure 2:**
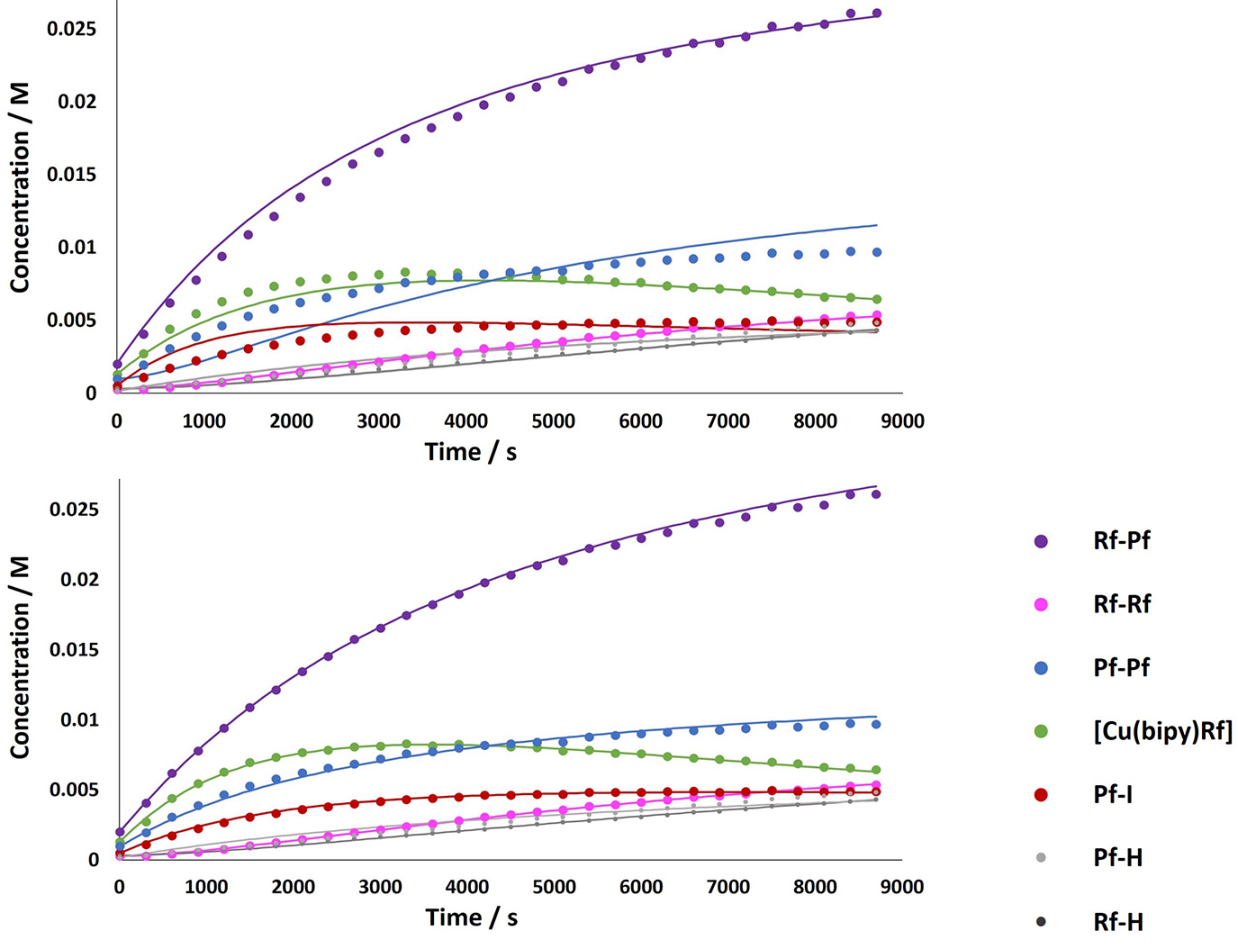
Experimental (dots) and best least-squares fitting (solid lines) of the products formed in the reaction of **1** (7.1 × 10^–2^ M) with Rf–I (7.1 × 10^–2^ M) in THF at 25 °C: (purple) Rf–Pf; (pink) Rf–Rf; (blue) Pf–Pf; (green) [Cu(bipy)(Rf)]; (red) Pf–I; (dark-gray) Rf–H; (light-gray) Pf–H. The upper figure shows the fitting with model I and the lower that with model II. Note the bad fitting on the upper figure, particularly for the homocoupling products Pf–Pf and Pf–I (blue and red).

**Figure 3:**
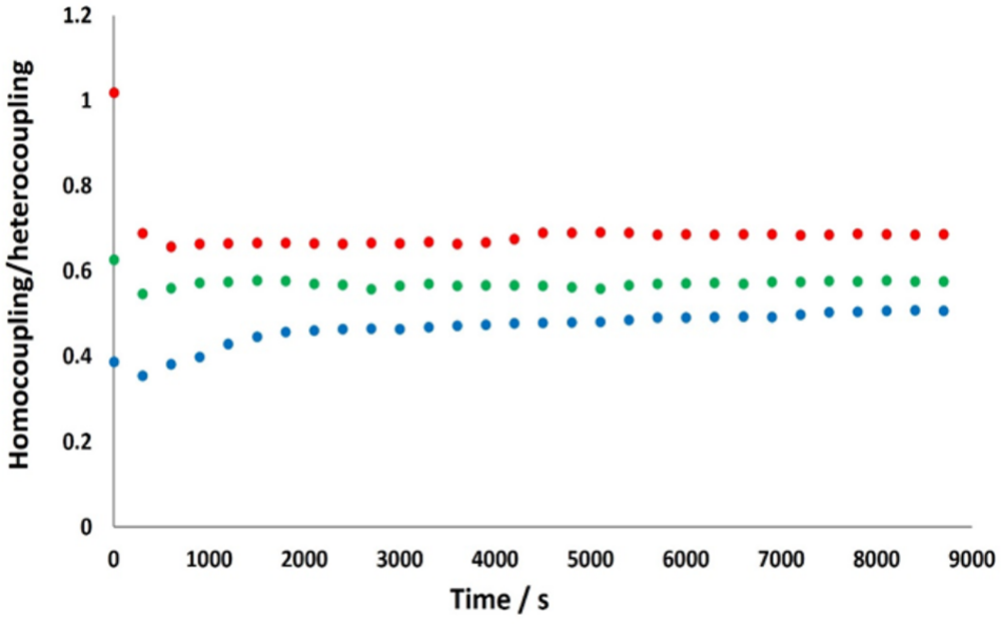
Homocoupling/heterocoupling ratio during the course of the reaction of **1** with Rf–I (7.1 × 10^–2^ M) in THF at 25 °C in reactions with different initial concentration of **1**. The homocoupling accounts for the sum of Pf–Pf plus Rf–Rf: (red dots) [**1**]_0_ = 1.07 × 10^–1^ M; (green dots) [**1**]_0_ = 7.1 × 10^–2^ M; (blue dots) [**1**]_0_ = 2.4 × 10^–2^ M.

Because [Cu(bipy)(Rf)] is also a reaction product, Pf–Pf and Rf–Rf should be formed at the same pace.^33^Figure 2 shows the formation of products starting from an equimolar mixture of **1** and Rf–I and the best nonlinear fittings obtained using models I and II (see the SI for further details) showing the different rates of formation of these two products. Also, the selectivity of the reaction agrees with the involvement of a second copper unit: In model I, the overall selectivity homocoupling/heterocoupling depends competitively on the rates of the reductive elimination of C–C and C–I. On the contrary, in model II, the selectivity depends on the relative kinetic rates of the reductive elimination and transmetalation from the intermediate [Cu(bipy)I(Pf)(Rf)] (**I1**) and then on the concentration of **1**. Figure 3 represents the evolution of the homo/heterocoupling ratio in reactions with different initial concentrations of **1**. Consistent with model II, the amount of homocoupling products increases with [**1**]_0_.

### DFT Calculations

**Figure 4:**
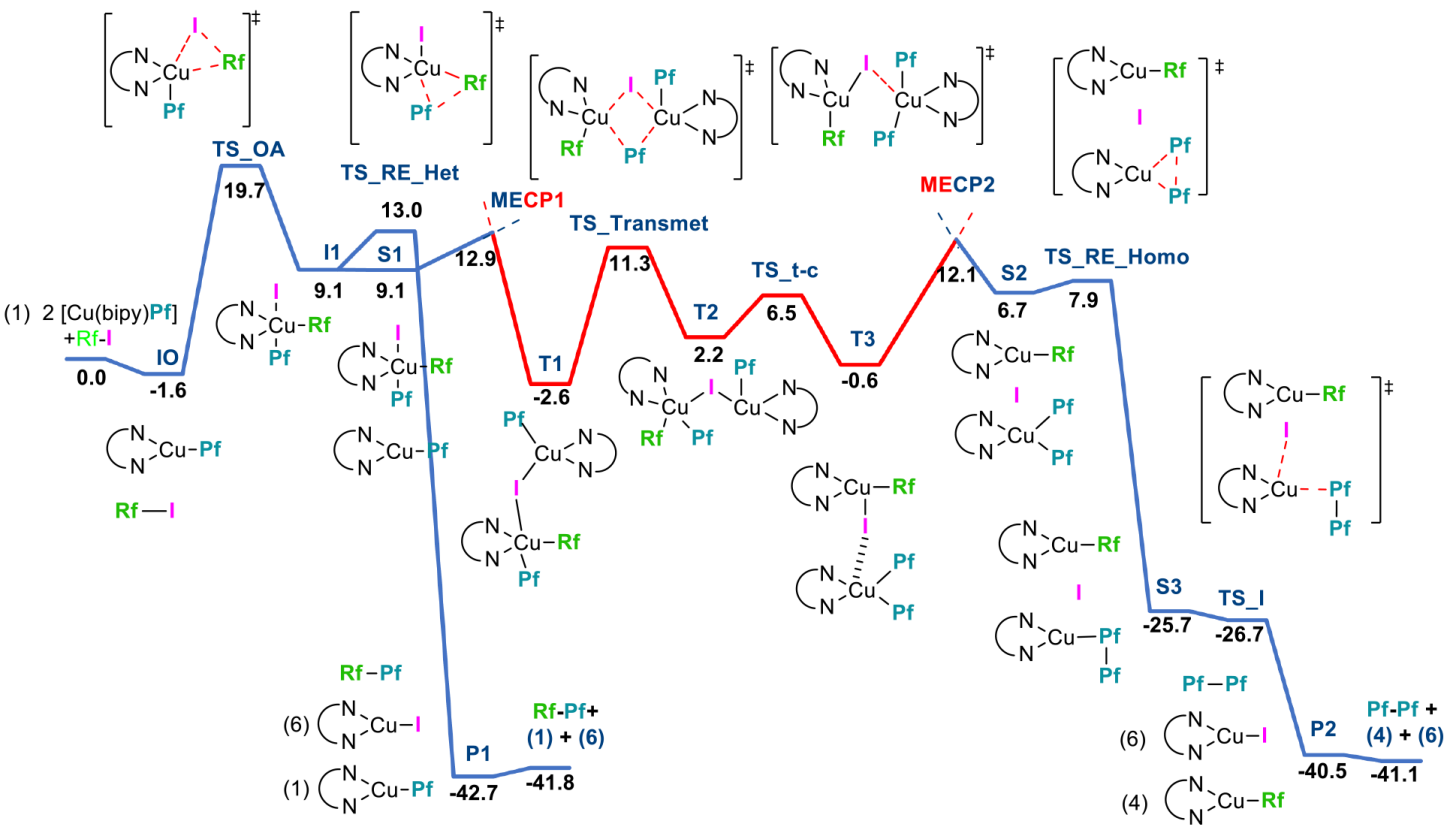
DFT-computed (B3LYP-D3/BS2 functional in THF) pathways for the competitive reactions of the formation of heterocoupling and homocoupling products. Relative Gibbs energies are given in kcal mol^–1^. Blue line: singlet potential energy surface. Red line: triplet potential energy surface. **S1**, **S2**, and **S3** represent intermediates in the singlet state and **T1**, **T2**, and **T3** intermediates in the triplet state. Optimized structures of all of the species in the profile are displayed in the SI.

Thus, from these results, it seems clear that some kind of transmetalation reaction is involved in the formation of homocoupling products, although the intimate mechanism cannot be established with the available experimental data. To disclose the mechanism of such a transformation, we have carried out DFT calculations (B3LYP-D3 functional with a SMD (solvation model based on density)/continuum description of the tetrahydrofuran (THF) solvent (see Computational Details in the SI). The calculated Gibbs energy profile for the reaction between **1** and Rf–I is shown in Figure 4

Heterocoupling follows the expected pathway. The oxidative addition of Rf–I to **1** takes place with an energy barrier of 21.3 kcal mol^–1^ (**TS_OA**), yielding the Cu(III) pentacoordinate intermediate **I1**,^34^ which affords the heterocoupling product Rf–Pf after crossing a low barrier (3.9 kcal mol^–1^; **TS_RE_Het**) for the reductive elimination step. An important point evidenced by the calculation is the instability of the Cu(III) intermediate **I1** that leads to a very low barrier (3.9 kcal mol^–1^) for the reductive elimination step.

**Figure 5:**
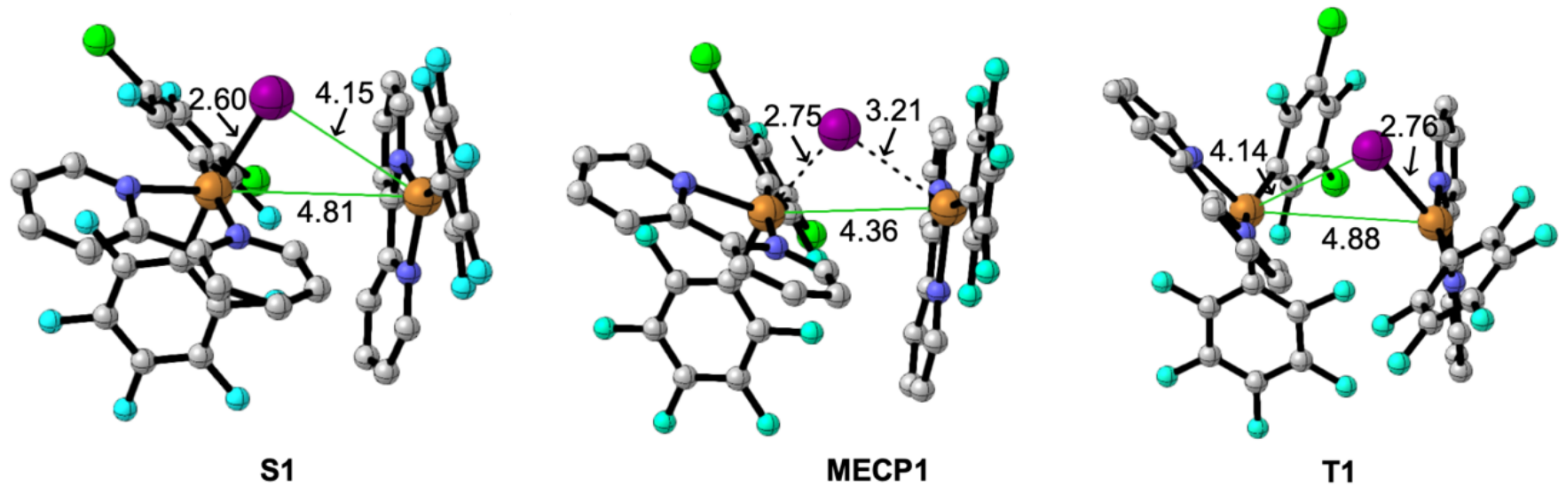
Optimized geometries of the singlet (**S1**) and triplet (**T1**) intermediates and the corresponding minimum-energy crossing point connecting both (**MECP1**). Distances are in angstroms.

Because homocoupling requires the presence of a second copper center, we included in the calculation one molecule of the Cu(I) reagent **1**, present in the medium in a large excess. The presence of **1** has no effect in the stability of **I1** in the singlet state (**S1** in Figure 4 at 9.1 kcal mol^–1^). However, the optimization of **S1** (the couple **I1**·**1**) in the triplet state causes dramatic changes in the system: a much more stable intermediate **T1** (at −2.6 in the Gibbs energy profile; Figure 4) is formed as a consequence of a comproportionation reaction between the Cu(III) and Cu(I) centers. The jump from the singlet **S1** [Cu(IIII)–Cu(I)] to the triplet **T1** [formally a Cu(II)–Cu(II) system, see copper atomic charges in **I0**, **S1**, and **T1** and the spin-density plot for **T1** in the SI] takes place through a minimum-energy crossing point (**MECP1**) at 12.9 kcal mol^–1^. The electron transfer between the two copper complexes happens with the simultaneous exchange of an iodine atom. The optimized geometries of **S1**, **MECP1**, and **T1** are depicted in Figure 5.

To assess the reliability of the singlet–triplet crossing, the relative energies of the singlet **S1** and triplet **T1** intermediates have been computed with 12 functionals containing variable percentages of Hartree–Fock exchange, using an extended basis set (BS2; see the SI). Although this energy difference is very dependent on the functional, all of the functionals agree that **T1** is more stable than **S1**.

**Figure 6:**
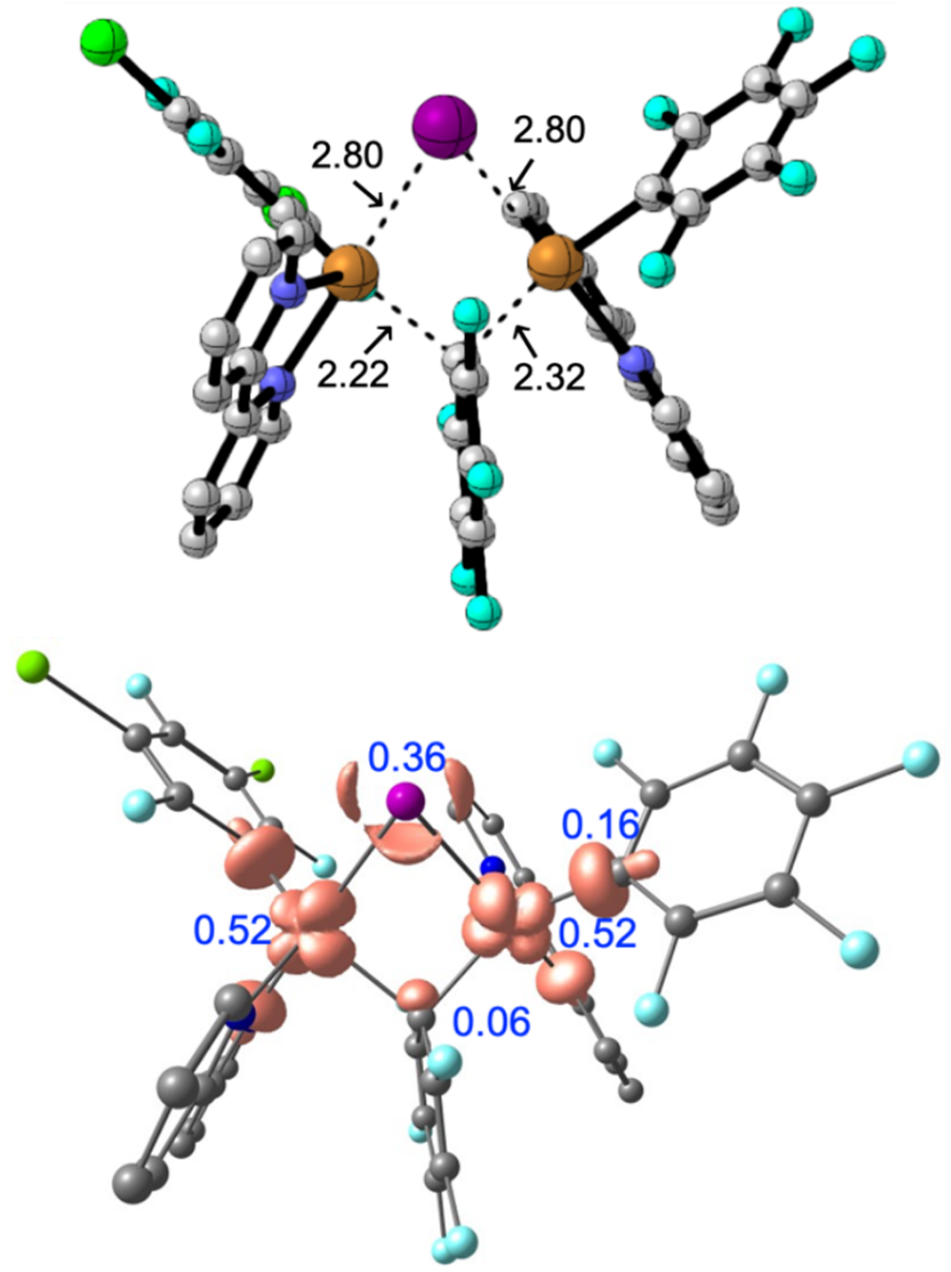
Optimized structure of the transmetalation transition state (**TS_Transmet**) (upper figure) and its spin-density plot (lower figure). In black are the distances (in angstroms), and in blue are the Mulliken spin populations.

From **T1**, an easy transmetalation of Pf happens (through **TS-Transmet**, Figure 6), placing both Pf groups bonded to the same copper center, although mutually trans. Then, through a low barrier isomerization **(TS_t-c**, Figure 4), the *cis*-Pf intermediate is reached (**T3**). Recrossing from the triplet (**T3**) to singlet (**S2**) potential energy surfaces through **MECP2** entails, in addition to electron transfer between the two copper centers, the detachment of an iodide. In **S2**, Cu(I) [Cu(bipy)(Rf)], Cu(III) *cis*-[Cu(bipy)(Pf_2_)]^+^, and I^–^ are present, and Pf–Pf homocoupling occurs in the Cu(III) center with a barrier of only 1.2 kcal mol^–1^ (**TS_RE_Homo**). A practically barrierless replacement of Pf–Pf by I^–^ in the coordination sphere of one copper ion (**TS_I**) yields the homocoupling product (Pf–Pf) and two copper(I) complexes, [Cu(bipy)(Rf)] and [Cu(bipy)I].

The computed profile depicts a situation in which the heterocoupling is a unimolecular process from the intermediate **I1**, which is consistent with a kinetic reaction order of 1 on **1** for this reaction. The selectivity for the formation of homo- or heterocoupling products depends on the relative energies of **MECP1** and **TS_RE_Het** and also on the concentration of **1**, but because the selection takes place after the rate-limiting step (oxidative addition), the overall kinetic order on **1** is 1.

Homocoupling requires the presence of a Cu(III) intermediate that can cross to the triplet potential energy surface by a comproportionation reaction with a Cu(I) center. As mentioned above, heterocoupling Pf–Rf is the only observable reaction for nonfluorinated aryls.^35^ We have computed the energy profiles for a series of nonfluorinated para-substituted aryl iodides (R = H, OMe, Br, NO_2_; see Figure 1). The computed Gibbs energy profiles for all of these nonfluorinated Ar–I (see the SI) show that no metastable Cu(III) [Cu(bipy)I(Pf)(Ph)] intermediate is formed in this reaction, with the Ar–Pf product being reached in only one step. Therefore, the comproportionation reaction with a Cu(I) intermediate cannot occur. Accordingly, transmetalation does not happen either and homocoupling products are not observed. When I–Pf or I–Rf are used, the Cu(III) intermediates benefit from the large stabilization of the C–M bonds that fluoroaryls provide,^31^ and intermediate **I1** is stable enough to be captured by **1**, allowing electron transfer between both copper centers to occur, yielding a biradical Cu(II)–Cu(II) system. A fast bimolecular scrambling of aryls may take place between the Cu(II) centers, leading to a statistical formation of copper complexes with two Pf groups or with one Pf group and one Rf group. From this fast-exchange system, Pf–Pf and Pf–Rf can be formed and also [Cu(bipy)(Rf)] through the reductive elimination of Pf–I.

Finally, it is worth noting the stability of the triplets **T1**, **T2**, and **T3** compared with the singlet Cu(III)/Cu(I) pairs. All of them are neutral species with bridging iodine, readily accessible from Cu(III) intermediates. These Cu(II) intermediates are kinetically labile and open new reaction pathways that cannot be predicted when only one molecule of the catalyst is considered, and they correlate with the reaction mechanisms of single copper species with binuclear complexes and with bioinorganic structures.^36^ In our case study, the pathway available in Cu(II) is transmetalation, which apparently is not accessible if only Cu(I) and Cu(III) species are considered.

## Conclusion

Our study presents a case in which the abundant Cu(I) reagents intercept Cu(III) intermediates in an electron-transfer comproportionation reaction, followed by a fast transmetalation. This comproportionation reaction can also be described as an inner-sphere electron transfer coupled with a spin surface crossing. From a kinetic point of view, the participation of two copper molecules in the reaction is somewhat hidden by the fact that it takes place after the rate-limiting step, the oxidation of Cu(I) to Cu(III), although it has a clear effect on the selectivity. DFT calculations clearly show the easiness of the electron transfer and transmetalation between copper complexes. We think that this model represents well reactive systems that go through Cu(III) intermediates. In this regard, some of us have recently reported comproportionation reactions between Cu(III), Cu(0), and Cu(I) centers, yielding Cu(II) species in oxygen-bridged polynuclear copper complexes.^37^ In these systems, the concentration of Cu(I) species accounts for most of the overall copper, making very probable the event of its reaction with complexes in other oxidation states. In this case, the bimolecular comproportionation has to be taken into consideration in mechanistic proposals.

## Supporting Information Available

Experimental section, control experiments, kinetic data, kinetic simulations and NMR spectra, crystallographic data for (NBu_4_)[Cu(C_6_F_5_)_2_], and computational methods and extended description of the computational results (PDF)

## Accession Codes

CCDC 2072143 contains the supplementary crystallographic data for this paper. These data can be obtained free of charge via www.ccdc.cam.ac.uk/data_request/cif, or by emailing data_request@ccdc.cam.ac.uk, or by contacting The Cambridge Crystallographic Data Centre, 12 Union Road, Cambridge CB2 1EZ, UK; fax: +44 1223 336033.

## References

[ref1] aNelson T. D.; Crouch R. D. Organic Reactions; John Wiley & Sons, Inc., 2004; pp 265–555, Vol. 63.

[ref2] Jones G. O.; Liu P.; Houk K. N.; Buchwald S. L. Computational Explorations of Mechanisms and Ligand-Directed Selectivities of Copper-Catalyzed Ullmann-Type Reactions. J. Am. Chem. Soc. 2010, 132, 6205–6213. 10.1021/ja100739h. 20387898 PMC2908497

[ref3] Beletskaya I. P.; Cheprakov A. V. The Complementary Competitors: Palladium and Copper in C–N Cross-Coupling Reactions. Organometallics 2012, 31, 7753–7808. 10.1021/om300683c.

[ref4] Hirano K.; Miura M. Recent Advances in Copper-Mediated Direct Biaryl Coupling. Chem. Lett. 2015, 44, 868–873. 10.1246/cl.150354.

[ref5] aThapa S.; Shrestha B.; Gurung S. K.; Giri R. Copper-Catalysed Cross-Coupling: An Untapped Potential. Org. Biomol. Chem. 2015, 13, 4816–4827. 10.1039/C5OB00200A. 25829351

[ref6] Hickman A. J.; Sanford M. S. High-valent organometallic copper and palladium in catalysis. Nature 2012, 484, 177–185. 10.1038/nature11008. 22498623 PMC4384170

[ref7] Casitas A.; Ribas X. The Role of Organometallic Copper(Iii) Complexes in Homogeneous Catalysis. Chem. Sci. 2013, 4, 2301–2318. 10.1039/c3sc21818j.

[ref8] Li S.-J.; Lan Y. Is Cu(III) a Necessary Intermediate in Cu-Mediated Coupling Reactions? A Mechanistic Point of View. Chem. Commun. 2020, 56, 6609–6619. 10.1039/D0CC01946A. 32441282

[ref9] Zhang S.-L.; Liu L.; Fu Y.; Guo Q.-X. Theoretical Study on Copper(I)-Catalyzed Cross-Coupling between Aryl Halides and Amides. Organometallics 2007, 26, 4546–4554. 10.1021/om700573h.

[ref10] Giri R.; Brusoe A.; Troshin K.; Wang J. Y.; Font M.; Hartwig J. F. Mechanism of the Ullmann Biaryl Ether Synthesis Catalyzed by Complexes of Anionic Ligands: Evidence for the Reaction of Iodoarenes with Ligated Anionic CuI Intermediates. J. Am. Chem. Soc. 2018, 140, 793–806. 10.1021/jacs.7b11853. 29224350 PMC5810543

[ref11] Yu H.-Z.; Jiang Y.-Y.; Fu Y.; Liu L. Alternative Mechanistic Explanation for Ligand-Dependent Selectivities in Copper-Catalyzed N- and O-Arylation Reactions. J. Am. Chem. Soc. 2010, 132, 18078–18091. 10.1021/ja104264v. 21133430

[ref12] Kalkman E. D.; Mormino M. G.; Hartwig J. F. Unusual Electronic Effects of Ancillary Ligands on the Perfluoroalkylation of Aryl Iodides and Bromides Mediated by Copper(I) Pentafluoroethyl Complexes of Substituted Bipyridines. J. Am. Chem. Soc. 2019, 141, 19458–19465. 10.1021/jacs.9b10540. 31722521 PMC11620760

[ref13] Tye J. W.; Weng Z.; Johns A. M.; Incarvito C. D.; Hartwig J. F. Copper Complexes of Anionic Nitrogen Ligands in the Amidation and Imidation of Aryl Halides. J. Am. Chem. Soc. 2008, 130, 9971–9983. 10.1021/ja076668w. 18597458 PMC2819338

[ref14] Tye J. W.; Weng Z.; Giri R.; Hartwig J. F. Copper(I) Phenoxide Complexes in the Etherification of Aryl Halides. Angew. Chem., Int. Ed. 2010, 49, 2185–2189. 10.1002/anie.200902245. PMC3008396 20198667

[ref15] Huang Z.; Hartwig J. F. Copper(I) Enolate Complexes in α-Arylation Reactions: Synthesis, Reactivity, and Mechanism. Angew. Chem., Int. Ed. 2012, 51, 1028–1032. 10.1002/anie.201106719. PMC3324266 22162321

[ref16] Konovalov A. I.; Lishchynskyi A.; Grushin V. V. Mechanism of Trifluoromethylation of Aryl Halides with CuCF_3_and the Ortho Effect. J. Am. Chem. Soc. 2014, 136, 13410–13425. 10.1021/ja507564p. 25222650

[ref17] Lin X.; Hou C.; Li H.; Weng Z. Decarboxylative Trifluoromethylating Reagent [Cu(O_2_CCF_3_)(Phen)] and Difluorocarbene Precursor [Cu(Phen)_2_][O_2_CCF_2_Cl]. Chem. - Eur. J. 2016, 22, 2075–2084. 10.1002/chem.201504306. 26756573

[ref18] Lu Z.; Liu H.; Liu S.; Leng X.; Lan Y.; Shen Q. A Key Intermediate in Copper-Mediated Arene Trifluoromethylation, [NBu_4_N][Cu(Ar)(CF_3_)_3_]: Synthesis, Characterization, and C(sp^2^)–CF_3_ Reductive Elimination. Angew. Chem., Int. Ed. 2019, 58, 8510–8514. 10.1002/anie.201904041. 31004379

[ref19] Paeth M.; Tyndall S. B.; Chen L. Y.; Hong J. C.; Carson W. P.; Liu X.; Sun X.; Liu J.; Yang K.; Hale E. M.; Tierney D. L.; Liu B.; Cao Z.; Cheng M.-J.; Goddard W. A. III; Liu W. Csp^3^-Csp^3^ Bond-Forming Reductive Elimination from Well-Defined Copper(III) Complexes. J. Am. Chem. Soc. 2019, 141, 3153–3159. 10.1021/jacs.8b12632. 30678456

[ref20] aHoffmann R.; Alvarez S.; Mealli C.; Falceto A.; Cahill T. J.; Zeng T.; Manca G. From Widely Accepted Concepts in Coordination Chemistry to Inverted Ligand Fields. Chem. Rev. 2016, 116, 8173–8192. 10.1021/acs.chemrev.6b00251. 27398715

[ref21] Akatyev N.; Il’in M.; Il’in M. Jr.; Peregudova S.; Peregudov A.; Buyanovskaya A.; Kudryavtsev K.; Dubovik A.; Grinberg V.; Orlov V.; Pavlov A.; Novikov V.; Volkov I.; Belokon Y. Chan-Evans-Lam C-N Coupling Promoted by a Dinuclear Positively Charged Cu(II) Complex. Catalytic Performance and Some Evidence for the Mechanism of CEL Reaction Obviating Cu(III)/Cu(I) Catalytic Cycle. ChemCatChem 2020, 12, 3010–3021. 10.1002/cctc.202000212.

[ref22] delPozo J.; Casares J. A.; Espinet P. In Situ Generation of ArCu from CuF_2_ Makes Coupling of Bulky Aryl Silanes Feasible and Highly Efficient. Chem. - Eur. J. 2016, 22, 4274–4284. 10.1002/chem.201504435. 26895353

[ref23] Kim U. B.; Jung D. J.; Jeon H. J.; Rathwell K.; Lee S. Synergistic Dual Transition Metal Catalysis. Chem. Rev. 2020, 120, 13382–13433. 10.1021/acs.chemrev.0c00245. 33251788

[ref24] aPérez-Temprano M. H.; Casares J. A.; Espinet P. Bimetallic catalysis using transition and group 11 metals: An emerging tool for C-C coupling and other reactions. Chem. - Eur. J. 2012, 18, 1864–1884. 10.1002/chem.201102888. 22267102

[ref25] The DFT-computed Gibbs energy barrier (298.15 K) is 24.1 kcal mol^–1^, in very good agreement with the experimental value.

[ref26] Yoshikai N.; Nakamura E. Mechanisms of Nucleophilic Organocopper(I) Reactions. Chem. Rev. 2012, 112, 2339–2372. 10.1021/cr200241f. 22111574

[ref27] aLeoni P.; Pesquali M.; Ghilardi C. A. Isolation and Crystal and Molecular Structure of a Rare Example of a Mononuclear Organo-Cuprate. J. Chem. Soc., Chem. Commun. 1983, 6, 240–241. 10.1039/c39830000240.

[ref28] The equilibria show higher *K*_eq_ values when measured in more polar solvents. See also refs (13) and (14).

[ref29] aGiri R.; Hartwig J. F. Cu(I)–Amido Complexes in the Ullmann Reaction: Reactions of Cu(I)–Amido Complexes with Iodoarenes with and without Autocatalysis by CuI. J. Am. Chem. Soc. 2010, 132, 15860–15863. 10.1021/ja105695s. 20977264 PMC2990966

[ref30] This agrees with most of the proposals for oxidative addition reactions on trigonal Cu(I) intermediates. See, for instance, refs (5), (10), and (12).

[ref31] Clot E.; Mégret C.; Eisenstein O.; Perutz R. N. Exceptional Sensitivity of Metal-Aryl Bond Energies to Ortho-Fluorine Substituents: Influence of the Metal, the Coordination Sphere, and the Spectator Ligands on M-C/H-C Bond Energy Correlations. J. Am. Chem. Soc. 2009, 131, 7817–7827. 10.1021/ja901640m. 19453181

[ref32] Other possible pathways, such the formation of intermediates of the type [Cu(bipy)(Pf)(Rf)_2_], have been considered and are discussed in the SI.

[ref33] We have shown that C_6_F_5_ and C_6_Cl_2_F_3_ behave as electronically equivalent groups. For instance, see: Pérez-Iglesias M.; Lozano-Lavilla O.; Casares J. A. [Cu(C_6_Cl_2_F_3_)(tht)]_4_: An Extremely Efficient Catalyst for the Aryl Scrambling between Palladium Complexes. Organometallics 2019, 38, 739–742. 10.1021/acs.organomet.8b00885.

[ref34] This value fits nicely with the experimental value Δ*G*^⧧^ = 20.8 kcal mol^–1^ obtained from the kinetic data.

[ref35] Very small amounts of Pf–H and Pf–Pf are always formed when complex **1** is in solution for some hours due to adventitious amounts of water and oxygen in solution. See the SI.

[ref36] Desnoyer A. N.; Nicolay A.; Rios P.; Ziegler M. S.; Tilley T. D. Bimetallics in a Nutshell: Complexes Supported by Chelating Naphthyridine-Based Ligands. Acc. Chem. Res. 2020, 53, 1944–1956. 10.1021/acs.accounts.0c00382. 32878429

[ref37] Álvarez M.; Molina F.; Fructos M. R.; Urbano J.; Álvarez E.; Sodupe M.; Lledós A.; Pérez P. J. Aerobic Intramolecular Carbon–Hydrogen Bond Oxidation Promoted by Cu(I) Complexes. Dalton Trans. 2020, 49, 14647–14655. 10.1039/D0DT03198D. 33057511

