# Supplementary material for: Transmetalation Reactions Triggered by Electron Transfer between Organocopper Complexes
Source: Inorg Chem. 2021 Jul 14;60(15):11633–9. doi: 10.1021/acs.inorgchem.1c01595 (PMC8609523; doi:10.1021/acs.inorgchem.1c01595)
Supplement: Supplementary file 1 — ic1c01595_si_001.pdf [file ic1c01595_si_001.pdf]

Supporting Information

Transmetalation reactions triggered by electron transfer between organo-copper complexes

Olmo Lozano-Lavilla,[&] Pablo Gómez-Orellana,[§] Agustí Lledós,*[§], and Juan A. Casares[&]*

[&] IU CINQUIMA/Química Inorgánica, Facultad de Ciencias, Universidad de Valladolid, 47011-Valladolid (Spain).

[§] Departament de Química, Edifici C.n. Universitat Autònoma de Barcelona, 08193 Cerdanyola del Vallès, Catalonia, Spain.

* Corresponding author, e-mail: juanangel.casares@uva.es (J. A. Casares) and agusti@klingon.uab.es (A. Lledós)

Table of Contents

**S1** General methods and reagent availability	2
**S2** Synthetic procedures	2
**S3** Calculation of the ionization equilibrium constant at 298 K through Van´t Hoff plot	5
**S4** Experiments of pentafluorophenylation of non-perhalogenated aryl iodides	7
**S5** ^{19}F NMR monitoring of the reaction between compound 1 and $C_6Cl_2F_3I$	12
**S6** Control experiments	13
**S7** Kinetic data analysis:	15
**S8** NMR spectra	33
**S9** Concentration/time data from fitted kinetic experiments	37
**S10** X-ray crystallography: Structure of $[Cu(C_6F_5)_2](NBu_4)$	42
**S11** Computational Details	44
**S12** Optimized structures of all the intermediates and transition states in the reaction of [Cu(bipy)Pf] (**1**) with **Rf-I (**Gibbs energy profile in Figure 2 of the main text)	45
**S13** Spin-density plots	49
**S14** Gibbs energy profile for the reaction of [Cu(bipy)Pf] (**1**) with *p*-substituted phenyl iodides (*p*-X-Ar-I; X = H, OMe, Br, NO_2)	50
**S15** Optimized structures of all the intermediates and transition states in the reaction of [Cu(bipy)Pf] (**1**) with **Ph-I** (Gibbs energy profile in Figure S19)	51
**S16** DFT benchmark study of the relative stabilities of Cu^{I}-Cu^{III} (**S1**) and Cu^{II}-Cu^{II} (**T1**) species	52
**S17** Cartesian coordinates and absolute E and G energies in THF of the optimized structures	53
**S18** References	81

S1. General methods and reagent availability

All the manipulations were performed under N_2 atmosphere, using standard Schlenk techniques. Solvents were dried using a solvent purification system SPS-MD5 or distilled from appropriate drying agents under nitrogen according to literature,[1] and storing them over 3 Å zeolites for a week. Prior to its use, solvents were degassed by three freeze-pump-thaw cycles. NMR spectra were recorded on Bruker AV 400 or Varian Inova 500-MR instruments equipped with variable-temperature probes. All glassware was flame dried or dried overnight at 110 ºC and allowed to cool under vacuum. Chemical shifts are reported in ppm from tetramethylsilane (^{1}H and ^{13}C), CCl_3F (^{19}F), with positive shifts downfield, at 298 K unless otherwise stated. The temperature for the NMR probe was calibrated with a methanol standard.[2] For the ^{19}F NMR spectra registered in non-deuterated solvents, an internal coaxial tube containing acetone-d6 was used to maintain the lock ^{2}H signal.

Compounds $[Cu(C_6Cl_2F_3)(tht)]_4$,[3] $C_6Cl_2F_3I$,[4] $C_6Cl_2F_3H$,[5] $C_6Cl_2F_3$-$C_6Cl_2F_3$,[5] $[Cu(bipy)(C_6F_5)]$,[6] $[Cu(bipy)I]$,[7] and $[Cu(bipy)_2]BF_4$[8] were prepared according to reported procedures. The rest of reactants were available from commercial sources and used as received without further purification.

Numbering of the complexes:

$[Cu(bipy)(C_6F_5)]$ (**1**) $[Cu(C_6F_5)_2]^-$ (**2**) $[Cu(bipy)_2]^+$ (**3**)

$[Cu(bipy)(C_6Cl_2F_3)]$ (**4**) $[Cu(bipy)(C_6Cl_2F_3)(C_6F_5)I]$ (**5**) $[Cu(bipy)I]$ (**6**)

$[Cu(bipy)(C_6F_5)_2I]$ (**7**) $[Cu(bipy)(C_6Cl_2F_3)(C_6F_5)_2]$ (**8**)

S2. Synthetic procedures

Synthesis of $(NBu_4)[Cu(C_6F_5)_2]$

C_6F_5Br (555 µL, 4.45 mmol) was charged in a 100 mL Schlenk flask protected from the light. Et_2O (17 mL) was added and the solution was cooled to -60 ºC in a isopropanol bath cooled using a cryostator. Then, a solution of BuLi 1.6M in hexanes (2.72 mL, 4.35 mmol) was added and the mixture was stirred for 45 min. Freshly prepared $(NBu_4)[CuCl_2]$ (800 mg, 2.12 mmol) was then added and the temperature warmed to -40 ºC, the mixture was stirred during 4 h. After that, the bath was allowed to warm during another hour, at that moment the mixture is a light yellowish suspension. Solvents were removed under vacuum and the solid residue was stirred with 18 mL of CH_2Cl_2, then, solids were separated by filtering through Celite using a Schlenk frit and rinsed with

another 2 mL. The filtrates were collected in another 100 mL Schlenk flask and then, vacuum eliminated up to 13 mL. Hexane (24 mL) was slowly added on top of them forming a layer and the flask was put under N_2 pressure and stored at -32 ºC for 24 h. After that time, colourless crystals of $(NBu_4)[Cu(C_6F_5)_2]$ had precipitated, the yellowish liquors were quickly removed at -32 ºC using a syringe. The precipitate was washed with cold Et_2O (3 mL) and vacuum dried. Yield 923 mg, 68%. Calcd. for $C_{28}H_{36}CuF_{10}N$: C, 52.54; H, 5.67; N 2.19; Found: C, 52.52; H, 5.59; N, 2.16.

^1H NMR (500 MHz, $CDCl_3$): δ 3.00 (m, 2H), 1.49 (m, 2H), 1.32 (m, 2H), 0.92 (t, J = 7.3 Hz, 3H). ^{19}F NMR (470 MHz, $CDCl_3$): δ -111.21 (m, $2F^{ortho}$), -161.06 (t, J = 19.8 Hz, $1F^{para}$), -162.87 (m, $2F^{meta}$). $^{13}C\{^1H\}$ NMR (126 MHz, $CDCl_3$): δ 149.3 (dm, J = 219.5 Hz, C^{ortho}), 137.8 (dm, J = 243.1 Hz, C^{para}), 136.1 (dm, J = 253.1 Hz, C^{meta}), 128.3 (tm, J = 76.7 Hz, C^{ipso}) 58.8, 23.7, 19.5, 13.3.

The compound obtained contained crystals suitable for X-ray diffraction that allowed the obtention of the solid state structure of compound $(NBu_4)[Cu(C_6F_5)_2]$ (See S10)

Synthesis of $[Cu(bipy)(C_6Cl_2F_3)]$

$[Cu(C_6Cl_2F_3)(tht)]_4$ (130 mg, 0.10 mmol) and 2,2'-bipyridine (64 mg, 0.41 mmol) were charged in a 100 mL Schlenk flask. CH_2Cl_2 (8 mL) was added and the mixture was stirred for 15 min conforming an orange suspension. Then, hexane (12 ml) was added and the mixture was stirred for another 5 min. The orange precipitate was separated from the solution using a Schlenk frit, washed there with hexane (3 mL) and vacuum dried. Yield 138 mg, 82%. Calcd. for $C_{16}H_8Cl_2CuF_3N_2$: C, 45.79; H, 1.92; N 6.67; Found: C, 45.53; H, 1.90; N, 6.45.

^1H NMR (500 MHz, THF-d8): δ 8.96 (br s, 1H), 8.41 (d, J = 8.1 Hz 1H), 8.13 (dt, J_1 = 7.8, J_2 = 1.1 Hz, 1H), 7.69 (m, 1H). ^{19}F NMR (470 MHz, THF-d8): δ -84.23 (s, $2F^{ortho}$), -122.35 (s, $1F^{para}$). $^{13}C\{^1H\}$ NMR (126 MHz, THF-d8): δ 150.1, 138.9, 126.2, 121.3. Due to the low solubility and the instability of this compound, carbon atoms not linked to a hydrogen atom could not be detected.

Synthesis of $(NBu_4)[CuCl_2]$

This compound was synthesized by a method adapted from the bibliography.[9] CuCl (300 mg, 3.03 mmol) and $(NBu_4)Cl$ (842 mg, 3.03 mmol) were charged in a 100 mL Schlenk flask. CH_2Cl_2 (10 mL) was added and the mixture was stirred for 15 min conforming a light yellowish suspension. Solids were filtered off using a filtering paper plugged cannula and the filtrate was collected in another 50 mL Schlenk flask, where solvent was vacuum removed up to 2 mL. Then, Et_2O (4 ml) was slowly added on top of the filtrate

forming a layer, the flask was closed under N_2 pressure and stored at -32 ºC for 6h. After that time, colorless crystals of $(NBu_4)[CuCl_2]$ had precipitated, the yellowish liquors were quickly removed at -32 ºC using a syringe. The precipitate was washed with cold Et_2O (2x3 mL) and vacuum dried. Yield 920 mg, 79%. Calcd. for $C_{16}H_{36}Cl_2CuN$: C, 50.99; H, 9.63; N 3.72; Found: C, 50.82; H, 9.57; N, 3.69.

^{1}H NMR (500 MHz, $CDCl_3$): δ 3.24 (m, 2H), 1.66 (m, 2H), 1.45 (m, 2H), 1.01 (t, J = 7.3 Hz, 3H). $^{13}C\{^1H\}$ NMR (126 MHz, $CDCl_3$): δ 59.1, 24.1, 19.8, 13.7.

S3. Calculation of the ionization equilibrium constant at 298 K through Van´t Hoff plot

$$2\,[\mathrm{Cu(bipy)(C_6F_5)}] \rightleftharpoons [\mathrm{Cu(bipy)_2}]^+ + [\mathrm{Cu(C_6F_5)_2}]^-$$

$$K_{eq} = \frac{\left[[\mathrm{Cu(C_6F_5)_2}]^-\right]\cdot\left[[\mathrm{Cu(bipy)_2}]^+\right]}{\left[[\mathrm{Cu(bipy)(C_6F_5)}]\right]^2}$$

Scheme S1: Ionization equilibrium of complex **1** and its equilibrium constant.

A solution $5.94{\cdot}10^{-2}$ M of **1** was prepared by adding **1** (5.05 mg, 0.0131 mmol) and THF (2.20 mL) inside a screw cap NMR tube with the aid of a Schlenk NMR tube adaptor, a flame sealed coaxial capillary containing acetone-d_6 was added to keep the lock signal. After that, the tube was transferred to the NMR spectrometer previously set to 170 K. ^{19}F NMR spectra were recorded at 170, 176, 182, 185, 193 and 198 by raising the temperature of the spectrometer. Integration of the broad signals corresponding to the -*ortho* fluorine nuclei of the compounds lead to relative ratios of neutral and ionic compounds used to calculate K_{ion} at each temperature through van´t Hoff’s equation (eq. S1). Signals belonging to neutral and ionic complexes were told apart by comparison with the signals of compound $(NBu_4)[Cu(C_6F_5)_2]$ at that temperature.

$$\ln(K_{eq}) = -\frac{\Delta H}{RT} + \frac{\Delta S}{R} \qquad \text{eq. S1}$$

Rate at 170C	Rate at 176 K	Rate at 182 K	Rate at 185 K	Rate at 193 K	Rate at 198 K	Calcd. Rate at 298 K	K_{ion} at 298 K
1 : 0.32	1: 0.31	1 : 0.27	1 : 0.24	1 : 0.20	1 : 0.19	1 : 0.030	$8.73 \cdot 10^{-4}$

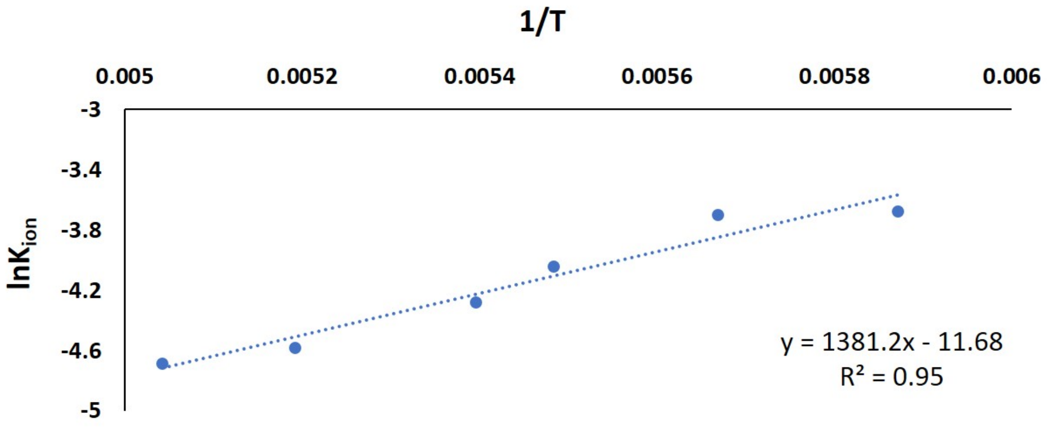

Figure S1: van´t Hoff plot for the ionic disproportion equilibrium of $[\mathrm{Cu(bipy)(C_6F_5)}]$.

DFT calculations give a ΔG of 4.4 kcal mol^{-1} for the ionization equilibrium in THF at 25ºC, that corresponds to a K_{eq} of $5.8x10^{-4}$, in very good agreement with the experimental measurement.

S4. Experiments of pentafluorophenylation of non-perhalogenated aryl halides

The reaction of complex **1** with an excess of iodobenzene in THF at 50 °C was monitored by ^{19}F NMR. The main product of this reaction is the coupling product C_6F_5-Ph. Also, significant amounts of the hydrolysis product C_6F_5H and trace amounts of the homocoupling product C_6F_5-C_6F_5 (~2% conversion) attributed to the reaction of compound **1** with O_2 (see below) could be detected. Non-linear fitting of the concentration/time data by non-linear least squares (NLLS) using the software COPASI[10] for the products C_6F_5-Ph and C_6F_5H lead to the obtainment of the kinetic constant for aryl heterocoupling. $k_{Ph\text{-}I}$ = 2.14·10^{-4} s^{-1}·M^{-1}.

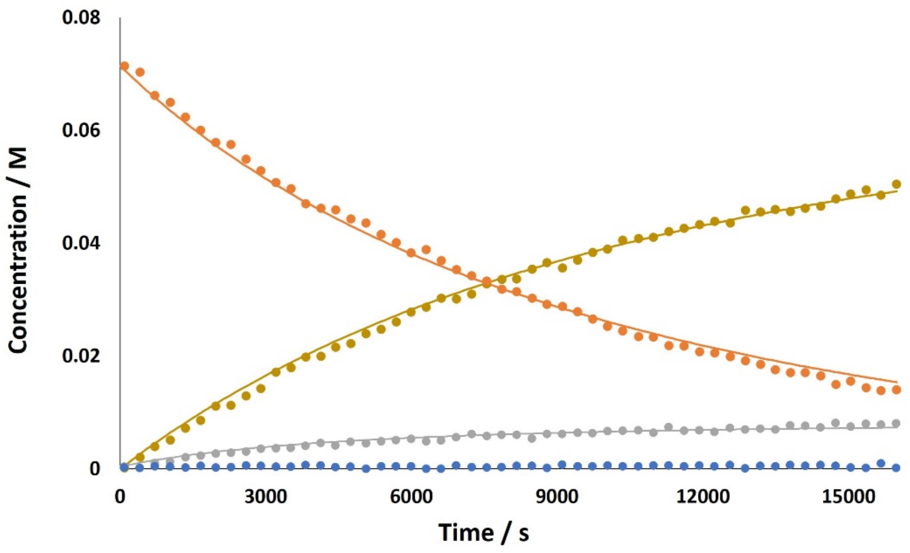

Figure S2: Experimental (dots) and best least square fitted (continuous line) concentration vs time plot of the formed species in the reaction of **1** (2.4·10^{-2}M) with PhI (4.3·10^{-1}M) in THF at 50 ºC. •**1**; •C_6F_5-Ph; •C_6F_5H; •C_6F_5-C_6F_5.

To study the effect of the electronic properties of the aryl iodides in the oxidative addition reaction to compound **1**, several competitive experiments containing *para*-substituted aryl iodides along with iodobenzene were carried out.

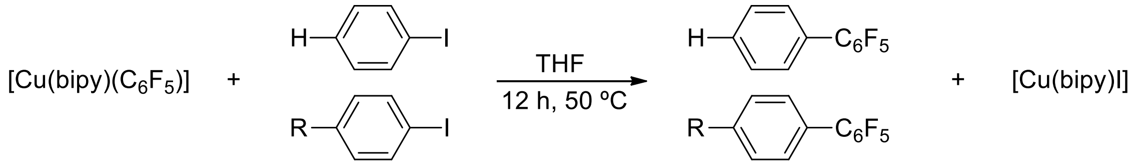

R = NO_2 CN CHO Br F OMe Me

9a **9b** **9c** **9d** **9e** **9f** **9g**

Scheme S2: Competitive reactions between complex **1**, iodobenzene and aryl iodides **9a-9g** in THF at 50 ºC.

Yields for the pentafluorophenylation reaction of each aryl iodide were derived from ^{19}F NMR spectra performed after the heating time, taking into account the amount of each aryl iodide added and the loss of reagent **1** due to the hydrolysis reaction with water traces. Relative reaction rates for each aryl iodide respect to the one of iodobenzene could be calculated and plotted against the Hammet parameters. (Figure 1 in main text)

Kinetic independent constants for each aryl iodide were calculated by comparison with the measured value for iodobenzene.

Table S1: Values of coupling constants for the reaction of aryl iodides **9a-9g** with complex **1** in THF at 50 ºC.

Ar-I	**9a**	**9b**	**9c**	**9d**	**9e**	**9f**	**9g**
k / $s^{-1}\cdot M^{-1}$	$2.23\cdot10^{-3}$	$1.36\cdot10^{-3}$	$1.32\cdot10^{-3}$	$4.04\cdot10^{-4}$	$2.10\cdot10^{-4}$	$1.59\cdot10^{-4}$	$1.50\cdot10^{-4}$

It has been reported that pentafluorophenylated Cu(I) complexes react with residual O_2 traces present in the media to yield the homocoupling product C_6F_5-C_6F_5.[12] However, the formation of C_6F_5-C_6F_5 could also be attributed to some other reactivity desrived from the interaction of complex **1** with the aryl halides. To rule out this possibility for the non-perhalogenated aryl halides, compound **1** was let react in the absence of aryl iodides and in the same conditions as in the competitive experiments.

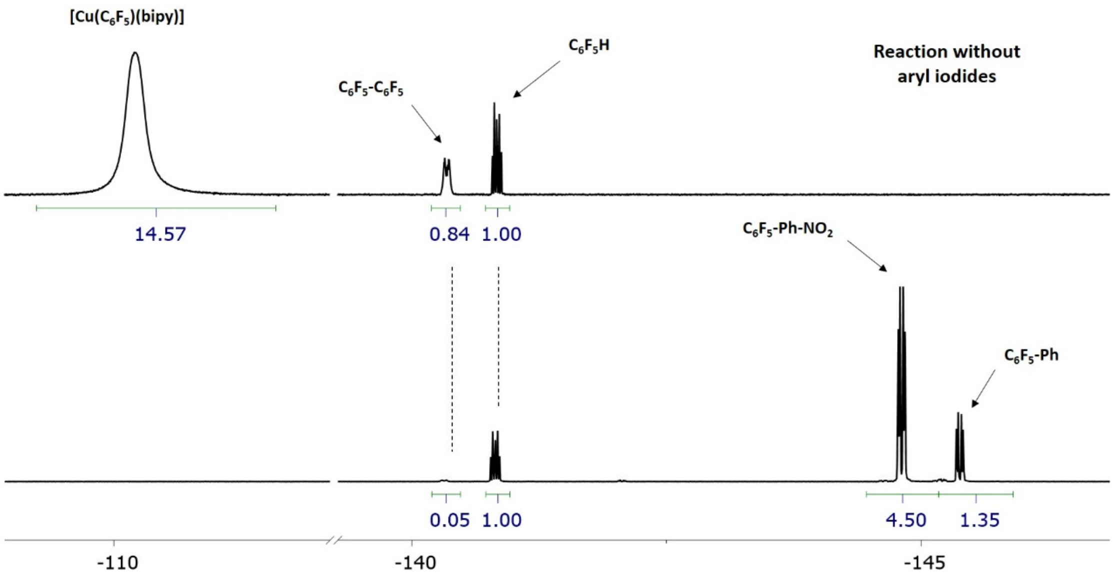

Figure S3: Comparation of ^{19}F NMR spectra in the *orto*- fluorine region of the standard reaction without aryl halides and the competitive reaction of iodobenzene and **9a** with complex **1** after 12 h at 50 ºC in THF.

This reaction yielded a much higher amount of the coupling product C_6F_5-C_6F_5 that the observed in the competitive experiments. These results imply that, in absence of aryl iodides, complex **1** reacts with both H_2O and O_2 impurities present in the medium being the latter reaction much slower. Once the water has been consumed, the remaining concentration of complex **1** is still high compared to the one in competitive experiments and O_2 can keep reacting with **1** to yield compound C_6F_5-C_6F_5.

Experimental procedure for monitorization and competitive experiments

Monitoring of the reaction of compound 1 with PhI

Complex **1** (35.74 mg, 0.092 mmol) was added inside a screw cap NMR tube with the aid of a Schlenk NMR tube adaptor along with a flame sealed coaxial capillary containing acetone-d_6 to keep the lock signal. The tube was cooled to -75 ºC in an isopropanol bath and 4,4'difluorobiphenyl (0.60 mg, 0.003 mmol), PhI (62 μL, 0.555 mmol) and THF (1.28 mL) were added. The tube was closed inside the adaptor and then, taken out of the cool bath, manually shaken until total dissolution of solids and transferred to the NMR probe, which had been preheated to the monitoring temperature (50 ºC).

Recording started after about 2 min required for the setup of the experiment, time zero for the measurements is taken at that moment. ^{19}F NMR spectra parameters are 64 scans, relaxation delay of 1 s, pulse angle of 30º, spectral width of 48076.9 Hz, and size of 32768 points. Spectra were collected every 300 s.

Preparation of the stock solution for competitive experiments

Compound **1** (369 mg, 0.955 mmol) was added into a Schlenk flask and successive cycles of vacuum/N_2 were applied, then, 11 mg (0.059 mmol) of 4,4'-difluorobiphenyl and 13.32 mL of THF were added. Manual stirring of the mixture lead to a clear red solution

Competitive experiments of aryl iodides 9a - 9e

A weighted amount of the aryl halide (0.105 mmol) and a volume of iodobenzene (35.5 μL, 0.317 mmol) were placed inside a screwed cap NMR tube with the aid of a Schlenk NMR tube adaptor. Then, 1.47 mL of a freshly prepared stock solution containing **1** (0.072 M) and 4,4'-difluorobiphenyl (0.0044 M) in THF were added. Finally, a flame sealed coaxial capillary containing acetone-d_6 was added and the tube was closed and manually shaken until dissolution of solids. The tubes were heated at 50 ºC in an oil bath for 12 h. In the case of aryl iodide **9b**, instead of a weighted amount of the compound, a volume of the compound was added using a 25 μL microsyringe.

Competitive experiments of aryl iodides 9f and 9g

A weighted amount of the aryl halide (0.317 mmol) and a volume of iodobenzene (11,8

μL, 0.105 mmol) were placed inside a screwed cap NMR tube with the aid of a Schlenk NMR tube adaptor, then, 1.47 mL of a freshly prepared stock solution containing **1** (0.072 M) and 4,4'-difluorobiphenyl (0.0044 M) in THF were added. Finally, a flame sealed coaxial capillary containing acetone-d_6 was added and the tube was closed and manually shaken until dissolution of solids. The tubes were heated at 50 ^oC for 12h in an oil bath.

S5. ^{19}F NMR monitoring of the reaction between compound 1 and $C_6Cl_2F_3I$

Monitoring of the reactions of compound 1 with $C_6Cl_2F_3I$

Weighted amounts of complex **1** were added inside a screw cap NMR tube with the aid of a Schlenk NMR tube adaptor along with a flame sealed coaxial capillary containing acetone-d_6 to keep the lock signal. The tube was cooled to -75 ºC in an isopropanol bath and a weighted amount of 4,4'difluorobiphenyl, THF and a weighted amount of $C_6Cl_2F_3I$ were added. The tube was closed inside the adaptor and then, taken out of the cool bath, manually shaken until total dissolution of solids and transferred to the NMR probe, which had been preheated to the monitoring temperature (25 ºC).

Table S2: Amounts of reagents used in the monitoring experiments of the reaction of compound **1** and $C_6Cl_2F_3I$

Exp.	Compound **1**	$C_6Cl_2F_3I$	4,4'-difluorobiphenyl	THF
1	15.34 mg<br>$7.1\cdot10^{-2}$ M	13.01 mg<br>$7.1\cdot10^{-2}$ M	0.88 mg<br>$1.3\cdot10^{-2}$ M	0.56 mL
2	23.25 mg<br>$1.07\cdot10^{-1}$ M	13.08 mg<br>$7.1\cdot10^{-2}$ M	0.96 mg<br>$9.0\cdot10^{-3}$ M	0.56 mL
3	5.15 mg<br>$2.4\cdot10^{-2}$ M	13.78 mg<br>$7.1\cdot10^{-2}$ M	0.96 mg<br>$8.6\cdot10^{-3}$ M	0.59 mL

Recording started after about 2 min required for the setup of the experiment, time zero for the measurements is taken at that moment. ^{19}F NMR spectra parameters are 64 scans, relaxation delay of 1 s, pulse angle of 30º, spectral width of 48076.9 Hz, and size of 32768 points. Spectra were collected every 300 s.

Values of concentration vs time were obtained by integration of ^{19}F NMR signals relative to the internal standard, 4,4'difluorobiphenyl. These values had to be corrected to compensate the different relaxation times of nuclei in different substances by applying a correction factor. Correction factors were obtained by measuring the integral of ^{19}F NMR experiments performed in the exact same conditions of the monitoring of samples containing mixtures of 4,4'difluorobiphenyl as internal standard and accurately weighted amounts of $(NBu_4)[Cu(C_6F_5)_2]$, $C_6Cl_2F_3I$, C_6F_5I, $C_6Cl_2F_3H$, C_6F_5H, $C_6Cl_2F_3$-$C_6Cl_2F_3$, C_6F_5-C_6F_5, $[Cu(bipy)(C_6F_5)]$ and $[Cu(bipy)(C_6Cl_2F_3)]$. The correction factor for the product $C_6Cl_2F_3$- C_6F_5 was estimated as the average of the homocoupling products.

S6. Control experiments

To test the capability of species $[Cu(C_6F_5)_2]^-$ to undergo oxidative addition reaction with aryl iodides, the reaction of compound $(NBu_4)[Cu(C_6F_5)_2]$ **2** with PhI was monitorized in the same conditions as in the reaction of compound **1** (See S4). After 6.7 h, coupling product C_6F_5-Ph was formed only in trace amounts >1%.

Also, the potential of species [Cu(bipy)I] and $[Cu(bipy)_2]^+$ to undergo oxidative addition reaction with aryl iodides was evaluated by letting the complexes [Cu(bipy)I] and $[Cu(bipy)_2]BF_4$ react with C_6F_5I in the same reaction conditions as in the monitoring experiments of the reaction between **1** and $C_6Cl_2F_3I$ (See S5) for 4 h. No coupling products containing the moiety C_6F_5 were detected.

To assess that the behavior of the system does not depend on the electronic properties of the complexes containing similar yet different aryl moieties, complex $[Cu(bipy)(C_6Cl_2F_3)]$ **4** was synthesized and its reaction with C_6F_5I in THF at 25 ^oC was monitored by ^{19}F NMR. Initial concentration of the reactants for this experiment differ from the used in the monitorization of the reaction of **1** and $C_6Cl_2F_3I$ due to solubility problems (See S5). Again, the formation of the homocoupling product $C_6Cl_2F_3$-$C_6Cl_2F_3$ containing the group that initially forms the organometallic reactant, shows a much higher rate of formation at the beginning of the reaction course than C_6F_5-C_6F_5.

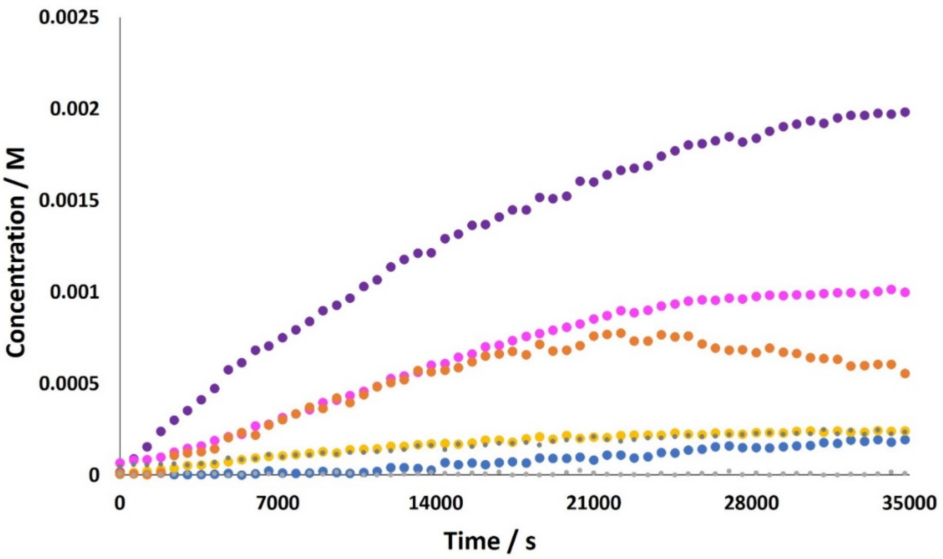

Figure S4: Concentration vs time plot of the formed species in the reaction of **4** ($5.8\cdot10^{-3}$ M) with C_6F_5I ($1.2\cdot10^{-2}$ M) in THF at 25 ºC.
•**1**; •$C_6Cl_2F_3I$; • $C_6Cl_2F_3$-C_6F_5; •$C_6Cl_2F_3$-$C_6Cl_2F_3$; •C_6F_5-C_6F_5; •$C_6Cl_2F_3H$; •C_6F_5H. Data belonging to species **4** and C_6F_5I were omitted for clarity

Experimental procedures for control experiments

Monitoring of the reaction of compound 4 with C_6F_5I

Complex **4** (2.35 mg, 0.006 mmol) was added inside a screw cap NMR tube with the aid of a Schlenk NMR tube adaptor along with a flame sealed coaxial capillary containing acetone-d_6 to keep the lock signal. The tube was cooled to -75 ºC in an isopropanol bath and a weighted amount of 4,4'difluorobiphenyl (0.45 mg, 0.002 mmol), THF (0.96 mL) and C_6F_5I (1.5 µL, 0.011 mmol) were added. The tube was closed inside the adaptor and then, taken out of the cool bath, manually shaken until total dissolution of solids and transferred to the NMR probe, which had been preheated to the monitoring temperature (25 ºC). The ^{19}F NMR recording started after about 2 min used for the setup of the experiment, time zero for the measurements is taken at that moment.

Monitoring of the reaction of Compound 2 with PhI

Complex **2** (25.60 mg, 0.040 mmol) was added inside a screw cap NMR tube with the aid of a Schlenk NMR tube adaptor along with a flame sealed coaxial capillary containing acetone-d_6 to keep the lock signal. The tube was cooled to -75 ºC in an isopropanol bath and a weighted amount of 4,4'difluorobiphenyl (2.50 mg, 0.013 mmol), THF (0.54 mL) and PhI (27 µL, 0.241 mmol) were added. The tube was closed inside the adaptor and then, taken out of the cool bath, manually shaken until total dissolution of solids and transferred to the NMR probe, which had been preheated to the monitoring temperature (50 ºC). The ^{19}F NMR recording started after about 2 min used for the setup of the experiment. After 6.6 h the conversion of complex **2** in C_6F_5-Ph was lower than 2%.

Reaction of [Cu(bipy)I] and $[Cu(bipy)_2]BF_4$ with C_6F_5I

Compounds [Cu(bipy)I] (50 mg, 0.142 mmol) or $[Cu(bipy)_2]BF_4$ (65 mg, 0.142 mmol) were added inside a 5 mL Schlenk flask along with a magnetic stir bar. Three vacuum/N_2 cycles were performed, then, C_6F_5I (18.9 µL, 0.142 mmol) and THF (2.00 mL) were added under nitrogen countercurrent. The flask was closed under nitrogen pressure and the red suspension was stirred for 4 h at room temperature. After that, the suspension was decanted and an aliquot of 0.5 mL was transferred inside an NMR with the aid of a Schlenk NMR tube adaptor along with a flame sealed coaxial capillary containing acetone-d_6 to keep the lock signal. The tube was closed inside the adaptor and transferred to the spectrometer to acquire a ^{19}F NMR spectrum.

S7. Kinetic data analysis

S7-1. Determination of the kinetic order of reaction of compound 1 in oxidative addition reactions with perhaloaryl iodides

Monitoring of the reactions of compound 1 with C_6F_5I

Weighted amounts of complex **1** were added inside a screw cap NMR tube with the aid of a Schlenk NMR tube adaptor along with a flame sealed coaxial capillary containing acetone-d_6 to keep the lock deuterium signal. The tube was cooled to -75 ºC in an isopropanol bath and a weighted amount of 4,4'difluorobiphenyl, THF and a volume of C_6F_5I taken with a microsyringe. The tube was closed inside the adaptor and then, taken out of the cool bath, manually shaken until total dissolution of solids and transferred to the NMR probe, which had been preheated to the monitoring temperature (25 ºC).

Table S3: Amounts of reagents used in the monitoring experiments of the reaction of compound **1** and C_6F_5I

Exp.	Compound **1**	C_6F_5I	4,4'-difluorobiphenyl	THF
1	23.13 mg<br>$7.1 \cdot 10^{-2}$ M	39.9 µL<br>$3.6 \cdot 10^{-1}$ M	0.77 mg<br>$4.8 \cdot 10^{-3}$ M	0.84 mL
2	27.44 mg<br>$5.1 \cdot 10^{-2}$ M	66.4 µL<br>$3.6 \cdot 10^{-1}$ M	1.42 mg<br>$5.3 \cdot 10^{-3}$ M	1.40 mL
3	12.88 mg<br>$2.5 \cdot 10^{-2}$ M	61.7 µL<br>$3.6 \cdot 10^{-1}$ M	1.23 mg<br>$5.0 \cdot 10^{-3}$ M	1.30 mL
4	6.60 mg<br>$1.7 \cdot 10^{-2}$ M	47.8 µL<br>$3.6 \cdot 10^{-1}$ M	2.25 mg<br>$1.2 \cdot 10^{-2}$ M	1.01 mL
5	6.60 mg<br>$1.3 \cdot 10^{-2}$ M	70.3 µL<br>$3.6 \cdot 10^{-1}$ M	0.95 mg<br>$3.7 \cdot 10^{-3}$ M	1.34 mL

Recording started after about 2 min used for the setup of the experiment, time zero for the measurements is taken at that moment. ^{19}F NMR spectra parameters are 64 scans, relaxation delay of 1 s, pulse angle of 30º, spectral width of 48076 Hz, and size of 32768 points. Spectra were collected every 300 s.

Order of reaction for compound **1** in the reaction of **1** and C_6F_5I was determined by initial rates method, measuring the linear rates for the formation of C_6F_5-C_6F_5 up to 33% of total conversion.

Exp.	[**1**]$_0$ / M	[C_6F_5I]$_0$ / M	r_0 / s·M^{-1}
1	$7.1\cdot10^{-2}$	$3.6\cdot10^{-1}$	$4.5\cdot10^{-5}$
2	$5.1\cdot10^{-2}$	$3.6\cdot10^{-1}$	$3.5\cdot10^{-5}$
3	$2.6\cdot10^{-2}$	$3.6\cdot10^{-1}$	$2.0\cdot10^{-5}$
4	$1.7\cdot10^{-2}$	$3.6\cdot10^{-1}$	$9.6\cdot10^{-5}$
5	$1.3\cdot10^{-2}$	$3.6\cdot10^{-1}$	$8.1\cdot10^{-5}$

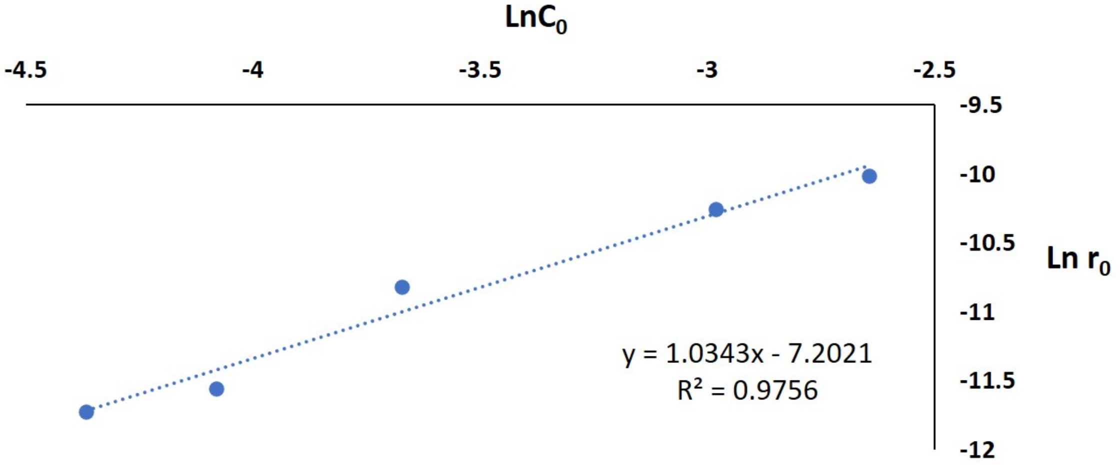

Figure S5: Experimental values of initial reaction rates and initial concentrations of compound **1** and plot of Ln(r_0) versus Ln(C_0)**.** The slope of the straight line is the kinetic order of the reaction on complex **1.**

S7-2. Kinetic models used for non-linear fitting of the concentration / time data of the reaction between compounds 1 and $C_6Cl_2F_3I$

Complex **1** reacts with $C_6Cl_2F_3I$ in THF at 25 °C producing the cross-coupling product $C_6Cl_2F_3$-C_6F_5, the homocoupling biaryls C_6F_5-C_6F_5 and $C_6Cl_2F_3$-$C_6Cl_2F_3$ and residual amounts of the hydrolysis products C_6F_5H and $C_6Cl_2F_3H$ (Scheme S3).

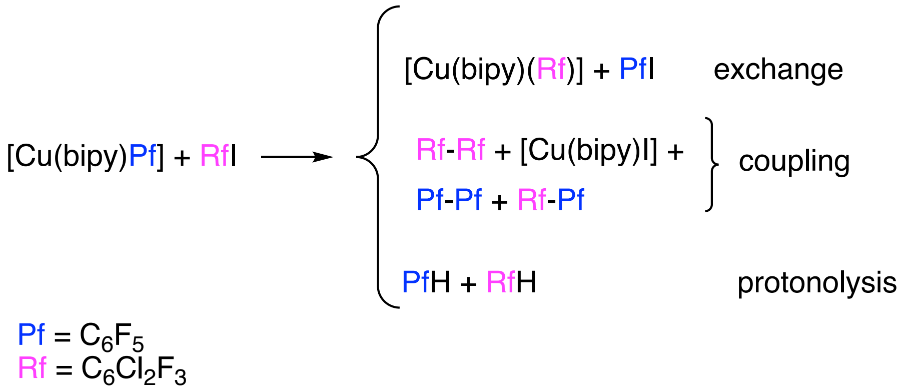

Scheme S3: Detected products in the reaction of **1** and $C_6Cl_2F_3I$ in THF at 25 ºC.

We have hypothesized two possible routes for the formation of the homocoupling products, in both of them the equilibrium of formation of the cuprate and the hydrolysis reactions have been taken into account. The model I includes consecutive oxidative-addition / reductive elimination equilibria leading to the aryl exchange process (Scheme S4). In this mechanism the formation of C_6F_5I and the complex [Cu(bipy)($C_6Cl_2F_3$)] (**4**) are merely the consequence of the reversibility of the oxidative addition step. The accumulation in solution of C_6F_5I enables the formation of the homocoupling product C_6F_5-C_6F_5 by reacting with the abundant complex **1** and the accumulation of [Cu(bipy)($C_6Cl_2F_3$)] (**4**) accounts for the formation of the homocoupling product $C_6Cl_2F_3$-$C_6Cl_2F_3$ by reacting with the aryl halide $C_6Cl_2F_3I$ which is used in large excess.

Model I

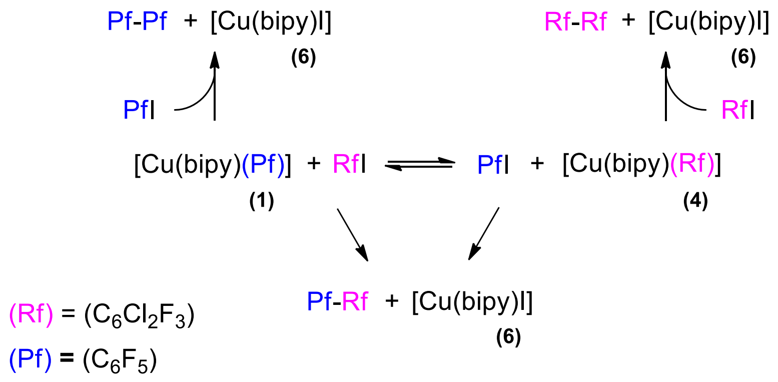

Scheme S4: Diagram of some of the reactions considered for model I explaining the formation of C_6F_5-C_6F_5

The second mechanism (model II) assumes as possible transmetalation reactions involving copper(I) and copper(III) complexes. Scheme S5 shows two possible pathways for the formation of C_6F_5-C_6F_5. In pathway "a" the transmetalation produces the exchange of aryls between Cu(I) and Cu(III) producing the observed Cu(I) complex **4** and [Cu(bipy)(C_6F_5)$_2$] (**7**), from which C_6F_5-C_6F_5 would be formed. Pathway "b" proposes the transmetalation of the aryl by the iodine atom, forming the triarylic intermediate [Cu(bipy)($C_6Cl_2F_3$)(C_6F_5)$_2$] (**8**) and [Cu(bipy)I] (**6**). From **8** the homocoupling or the cross-coupling product could be formed. For simplicity Scheme S5 does not show the pathways for the formation of $C_6Cl_2F_3$-$C_6Cl_2F_3$, but, since the concentration of **5** is small, it requires a double oxidative addition process. For this study we have assumed that the polifluorated groups ($C_6Cl_2F_3$) and (C_6F_5) behave as electronically equivalent groups, in agreement with the control experiment described above (See S6).[13]

Model II

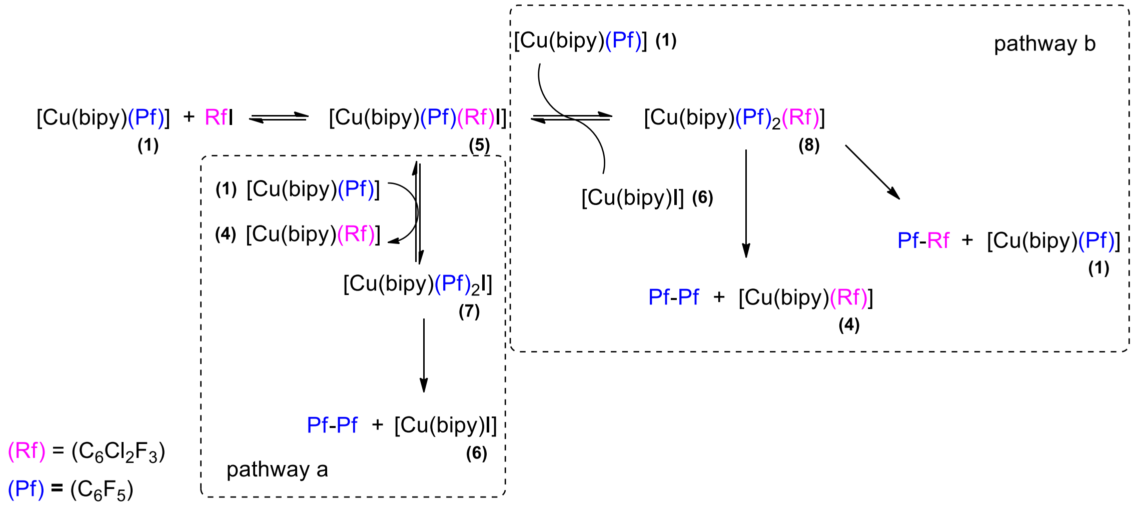

Scheme S5: Diagram of some of the reactions considered for model II explaining the formation of C_6F_5-C_6F_5

Figure 1 in the main text shows the experimental concentration time plot of the formation of $C_6Cl_2F_3$-$C_6Cl_2F_3$ and C_6F_5-C_6F_5 and the best fitting for both models. In both models the fitting of the formation of $C_6Cl_2F_3$-$C_6Cl_2F_3$ is quite good although model II fits better at long reaction times. That is the expected result because in both models $C_6Cl_2F_3$-$C_6Cl_2F_3$ requires the accumulation of **4** to take place. However, the models and the fitting differ substantially about the formation of C_6F_5-C_6F_5 (in blue in figure 1 main text). In this case the experimental results do not support the requirement of the accumulation of C_6F_5I which is implicit on model I. On the contrary, the experimental line and the fitting indicates that C_6F_5-C_6F_5 is formed independently of [C_6F_5I], and at high rate since the beginning of the reaction.

The kinetic models were fit to the final concentration / time data by non-linear least squares (NLLS) using the software COPASI.[10]

Used models in the non-linear fit with program COPASI

In all the models rate constants k_{ion} and k_{neutro} were forced to be related based on the measured value of K_{ion}.

$$K_{ion} = \frac{k_{ion}}{k_{neutro}} = 8.73 \cdot 10^{-4} \ ; \ k_{ion} = 8.73 \cdot 10^{-4} \cdot k_{neutro}$$

Also, values for the initial H_2O concentration and hydrolysis kinetic constants k_1 and k_2 were fitted by the program without restrictions.

Table S4: Model I containing ionic disproportionation equilibrium, an aryl methatesis mechanism, aryl coupling reactions and hydrolysis reactions.

Reaction
$[Cu(bipy)(C_6F_5)] + C_6Cl_2F_3I \underset{k_{met-1}}{\overset{k_{met}}{\rightleftharpoons}} [Cu(bipy)(C_6Cl_2F_3)] + C_6F_5I$
$[Cu(bipy)(C_6F_5)] + [Cu(bipy)(C_6Cl_2F_3)] \underset{k_{neutro}}{\overset{k_{ion}}{\rightleftharpoons}} [Cu(C_6Cl_2F_3)(C_6F_5)]^- + [Cu(bipy)_2]^+$
$2\ [Cu(bipy)(C_6Cl_2F_3)] \underset{k_{neutro}}{\overset{k_{ion}}{\rightleftharpoons}} [Cu(C_6Cl_2F_3)_2]^- + [Cu(bipy)_2]^+$
$2\ [Cu(bipy)(C_6F_5)] \underset{k_{neutro}}{\overset{k_{ion}}{\rightleftharpoons}} [Cu(C_6F_5)_2]^- + [Cu(bipy)_2]^+$
$[Cu(bipy)(C_6F_5)] + C_6Cl_2F_3I \xrightarrow{k_{coup1}} [Cu(bipy)I] + C_6Cl_2F_3\text{-}C_6F_5$
$[Cu(bipy)(C_6F_5)] + C_6F_5I \xrightarrow{k_{coup2}} [Cu(bipy)I] + C_6F_5\text{-}C_6F_5$
$[Cu(bipy)(C_6Cl_2F_3)] + C_6Cl_2F_3I \xrightarrow{k_{coup3}} [Cu(bipy)I] + C_6Cl_2F_3\text{-}C_6Cl_2F_3$
$[Cu(bipy)(C_6Cl_2F_3)] + C_6F_5I \xrightarrow{k_{coup4}} [Cu(bipy)I] + C_6Cl_2F_3\text{-}C_6F_5$
$[Cu(bipy)(C_6Cl_2F_3)] + H_2O \xrightarrow{k_2} [Cu(bipy)OH] + C_6Cl_2F_3H$
$[Cu(bipy)(C_6F_5)] + H_2O \xrightarrow{k_1} [Cu(bipy)OH] + C_6F_5H$

No restrictions were applied to the kinetic constants for the coupling reactions (Table S4 k_{coup} 1-4) involving C_6F_5 or $C_6Cl_2F_3$ groups in order to get the best fitting. When restrictions due to the similar behavior of C_6F_5 and $C_6Cl_2F_3$ groups were imposed ($k_{met} = k_{met-1}$; $k_{coup1} = k_{coup2} = k_{coup3} = k_{coup4}$) no fitting minimum could be reached.

Table S5: Model II pathway (a). Best fit reaction model containing ionic disproportionation equilibrium, reversible oxidative addition reactions leading to Cu(III) intermediates, a Cu(I)/Cu(III) transmetalation mechanism, biaryl reductive elimination reactions and hydrolysis reactions.

Reaction
$[Cu(bipy)(C_6Cl_2F_3)(C_6F_5)I] + [Cu(bipy)(C_6F_5)] \underset{k_{retro}}{\overset{k_{trans}}{\rightleftharpoons}} [Cu(bipy)(C_6F_5)_2I] + [Cu(bipy)(C_6Cl_2F_3)]$
$[Cu(bipy)(C_6Cl_2F_3)(C_6F_5)I] + [Cu(bipy)(C_6Cl_2F_3)] \underset{k_{retro}}{\overset{k_{trans}}{\rightleftharpoons}} [Cu(bipy)(C_6Cl_2F_3)_2I] + [Cu(bipy)(C_6F_5)]$
$[Cu(bipy)(C_6Cl_2F_3)] + C_6F_5I \underset{k_{x\text{-}i}}{\overset{k_{ad\text{-}ox}}{\rightleftharpoons}} [Cu(bipy)(C_6Cl_2F_3)(C_6F_5)I]$
$[Cu(bipy)(C_6F_5)] + C_6Cl_2F_3I \underset{k_{x\text{-}i}}{\overset{k_{ad\text{-}ox}}{\rightleftharpoons}} [Cu(bipy)(C_6Cl_2F_3)(C_6F_5)I]$
$[Cu(bipy)(C_6Cl_2F_3)] + C_6Cl_2F_3I \underset{k_{x\text{-}i}}{\overset{k_{ad\text{-}ox}}{\rightleftharpoons}} [Cu(bipy)(C_6Cl_2F_3)_2I]$
$[Cu(bipy)(C_6F_5)] + C_6F_5I \underset{k_{x\text{-}i}}{\overset{k_{ad\text{-}ox}}{\rightleftharpoons}} [Cu(bipy)(C_6F_5)_2I]$
$[Cu(bipy)(C_6F_5)] + [Cu(bipy)(C_6Cl_2F_3)] \underset{k_{neutro}}{\overset{k_{ion}}{\rightleftharpoons}} [Cu(C_6Cl_2F_3)(C_6F_5)]^- + [Cu(bipy)_2]^+$
$2\ [Cu(bipy)(C_6Cl_2F_3)] \underset{k_{neutro}}{\overset{k_{ion}}{\rightleftharpoons}} [Cu(C_6Cl_2F_3)_2]^- + [Cu(bipy)_2]^+$
$2\ [Cu(bipy)(C_6F_5)] \underset{k_{neutro}}{\overset{k_{ion}}{\rightleftharpoons}} [Cu(C_6F_5)_2]^- + [Cu(bipy)_2]^+$
$[Cu(bipy)(C_6Cl_2F_3)(C_6F_5)I] \xrightarrow{k_{elim}} [Cu(bipy)I] + C_6Cl_2F_3\text{-}C_6F_5$
$[Cu(bipy)(C_6F_5)_2I] \xrightarrow{k_{elim}} [Cu(bipy)I] + C_6F_5\text{-}C_6F_5$
$[Cu(bipy)(C_6Cl_2F_3)_2I] \xrightarrow{k_{elim}} [Cu(bipy)I] + C_6Cl_2F_3\text{-}C_6Cl_2F_3$
$[Cu(bipy)(C_6Cl_2F_3)] + H_2O \xrightarrow{k_2} [Cu(bipy)OH] + C_6Cl_2F_3H$
$[Cu(bipy)(C_6F_5)] + H_2O \xrightarrow{k_1} [Cu(bipy)OH] + C_6F_5H$

Table S6: Model II pathway (b). Alternative reaction model proposed containing ionic disproportionation equilibrium, reversible oxidative addition reactions leading to Cu(III) halogenated intermediates, a Cu(I)/Cu(III) transmetalation mechanism leading to triarylic Cu(III) species, biaryl reductive elimination reactions and hydrolysis reactions.

Reaction
$[Cu(bipy)(C_6Cl_2F_3)(C_6F_5)I] + [Cu(bipy)(C_6Cl_2F_3)] \underset{k_{trans}}{\overset{k_{retro}}{\rightleftharpoons}} [Cu(bipy)(C_6Cl_2F_3)_2(C_6F_5)] + [Cu(bipy)I]$
$[Cu(bipy)(C_6Cl_2F_3)(C_6F_5)I] + [Cu(bipy)(C_6F_5)] \underset{k_{trans}}{\overset{k_{retro}}{\rightleftharpoons}} [Cu(bipy)(C_6Cl_2F_3)(C_6F_5)_2] + [Cu(bipy)I]$
$[Cu(bipy)(C_6Cl_2F_3)_2I] + [Cu(bipy)(C_6Cl_2F_3)] \underset{k_{trans}}{\overset{k_{retro}}{\rightleftharpoons}} [Cu(bipy)(C_6Cl_2F_3)_3] + [Cu(bipy)I]$
$[Cu(bipy)(C_6Cl_2F_3)_2I] + [Cu(bipy)(C_6F_5)] \underset{k_{trans}}{\overset{k_{retro}}{\rightleftharpoons}} [Cu(bipy)(C_6Cl_2F_3)_2(C_6F_5)] + [Cu(bipy)I]$
$[Cu(bipy)(C_6F_5)_2I] + [Cu(bipy)(C_6Cl_2F_3)] \underset{k_{trans}}{\overset{k_{retro}}{\rightleftharpoons}} [Cu(bipy)(C_6Cl_2F_3)(C_6F_5)_2] + [Cu(bipy)I]$
$[Cu(bipy)(C_6F_5)_2I] + [Cu(bipy)(C_6F_5)] \underset{k_{trans}}{\overset{k_{retro}}{\rightleftharpoons}} [Cu(bipy)(C_6F_5)_3] + [Cu(bipy)I]$
$[Cu(bipy)(C_6Cl_2F_3)] + C_6F_5I \underset{k_{x\text{-}i}}{\overset{k_{ad\text{-}ox}}{\rightleftharpoons}} [Cu(bipy)(C_6Cl_2F_3)(C_6F_5)I]$
$[Cu(bipy)(C_6F_5)] + C_6Cl_2F_3I \underset{k_{x\text{-}i}}{\overset{k_{ad\text{-}ox}}{\rightleftharpoons}} [Cu(bipy)(C_6Cl_2F_3)(C_6F_5)I]$
$[Cu(bipy)(C_6Cl_2F_3)] + C_6Cl_2F_3I \underset{k_{x\text{-}i}}{\overset{k_{ad\text{-}ox}}{\rightleftharpoons}} [Cu(bipy)(C_6Cl_2F_3)_2I]$
$[Cu(bipy)(C_6F_5)] + C_6F_5I \underset{k_{x\text{-}i}}{\overset{k_{ad\text{-}ox}}{\rightleftharpoons}} [Cu(bipy)(C_6F_5)_2I]$
$[Cu(bipy)(C_6F_5)] + [Cu(bipy)(C_6Cl_2F_3)] \underset{k_{neutro}}{\overset{k_{ion}}{\rightleftharpoons}} [Cu(C_6Cl_2F_3)(C_6F_5)]^- + [Cu(bipy)_2]^+$
$2\ [Cu(bipy)(C_6Cl_2F_3)] \underset{k_{neutro}}{\overset{k_{ion}}{\rightleftharpoons}} [Cu(C_6Cl_2F_3)_2]^- + [Cu(bipy)_2]^+$
$2\ [Cu(bipy)(C_6F_5)] \underset{k_{neutro}}{\overset{k_{ion}}{\rightleftharpoons}} [Cu(C_6F_5)_2]^- + [Cu(bipy)_2]^+$
$[Cu(bipy)(C_6Cl_2F_3)_2(C_6F_5)] \xrightarrow{k_{elim}} [Cu(bipy)(C_6Cl_2F_3)] + C_6Cl_2F_3\text{-}C_6F_5$
$[Cu(bipy)(C_6Cl_2F_3)_2(C_6F_5)] \xrightarrow{2k_{elim}} [Cu(bipy)(C_6F_5)] + C_6Cl_2F_3\text{-}C_6Cl_2F_3$
$[Cu(bipy)(C_6Cl_2F_3)(C_6F_5)_2] \xrightarrow{k_{elim}} [Cu(bipy)(C_6F_5)] + C_6Cl_2F_3\text{-}C_6F_5$
$[Cu(bipy)(C_6Cl_2F_3)(C_6F_5)_2] \xrightarrow{2k_{elim}} [Cu(bipy)(C_6Cl_2F_3)] + C_6F_5\text{-}C_6F_5$
$[Cu(bipy)(C_6Cl_2F_3)_3] \xrightarrow{3k_{elim}} [Cu(bipy)(C_6Cl_2F_3)] + C_6Cl_2F_3\text{-}C_6Cl_2F_3$
$[Cu(bipy)(C_6F_5)_3] \xrightarrow{3k_{elim}} [Cu(bipy)(C_6F_5)] + C_6F_5\text{-}C_6F_5$
$[Cu(bipy)(C_6Cl_2F_3)] + H_2O \xrightarrow{k_2} [Cu(bipy)OH] + C_6Cl_2F_3H$
$[Cu(bipy)(C_6F_5)] + H_2O \xrightarrow{k_1} [Cu(bipy)OH] + C_6F_5H$

For the non-linear fitting using both pathways of model II, different complexes bearing groups (C_6F_5) and ($C_6Cl_2F_3$) were considered similar in terms of reactivity,[13] and therefore, reactions involving them are computed with the same kinetic constants:

k_{elim} for the reductive elimination irreversible reactions from Cu(III) species.

$k_{ad\text{-}ox}$ and $k_{x\text{-}I}$ for the oxidative addition equilibria between aryl iodides and Cu(I) arylated complexes.

k_{trans} and k_{retro} for the transmetalation equilibria between Cu(I) and Cu(III) complexes.

For model II Pathway b, in the reductive elimination reactions, constant k_{elim} is multiplied for a number representing the statistical probability for that reductive elimination to take place from the starting Cu(III) complex.

Even applying these restrictions, the fitting of model II pathway a is more appropriate than that obtained using model I (see below graphics of the models).

Data fitting using <u>model I</u>

- Exp. 1. Compound **1** / $C_6Cl_2F_3I$ 1 : 1

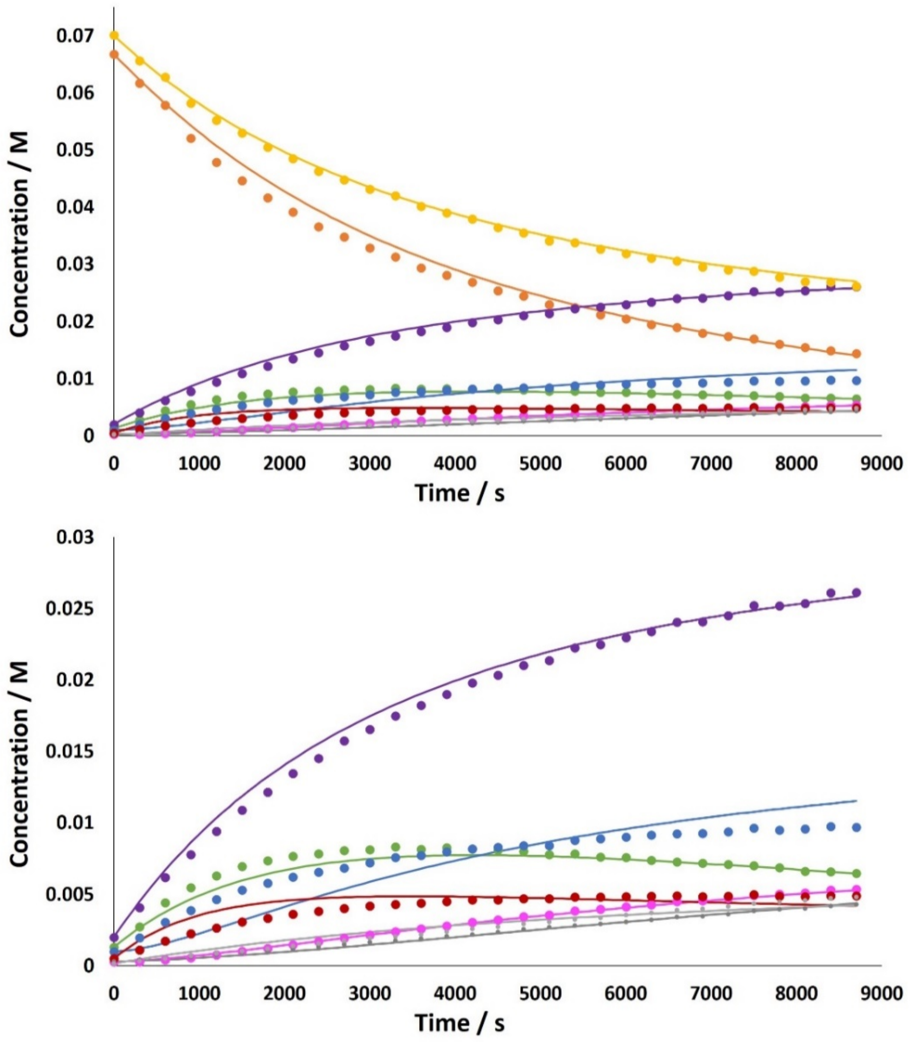

	k_{met}	k_{met-1}	k_{coup1}	k_{coup2}	k_{coup3}	k_{coup4}
Value / s^{-1}; $M^{-1}\cdot s^{-1}$	$1.1\cdot10^{-3}$	0	$1.9\cdot10^{-3}$	$9.7\cdot10^{-3}$	$2.2\cdot10^{-3}$	0
Std. deviation	$2.4\cdot10^{-5}$	$5.0\cdot10^{-3}$	$8.6\cdot10^{-5}$	$2.1\cdot10^{-4}$	$4.0\cdot10^{-4}$	$4.8\cdot10^{-3}$

Figure S6: Experimental (dots) and least square fit using <u>model I</u> (continuous line) concentration vs time plot of the formed species in the reaction of **1** ($7.1\cdot10^{-2}$ M) with $C_6Cl_2F_3I$ ($7.1\cdot10^{-2}$ M) in THF at 25 ºC. Both graphics represent the same experiment, in the lower one, the concentration vs time lines of the reagents have been omitted to show more clearly the fitting of the products. •**1**; •$C_6Cl_2F_3I$; •$C_6Cl_2F_3$-C_6F_5; •$C_6Cl_2F_3$-$C_6Cl_2F_3$; •C_6F_5-C_6F_5; •**4**; •C_6F_5I; •$C_6Cl_2F_3H$; •C_6F_5H.

- Exp. 2. Compound **1** / $C_6Cl_2F_3I$ 1.5 : 1

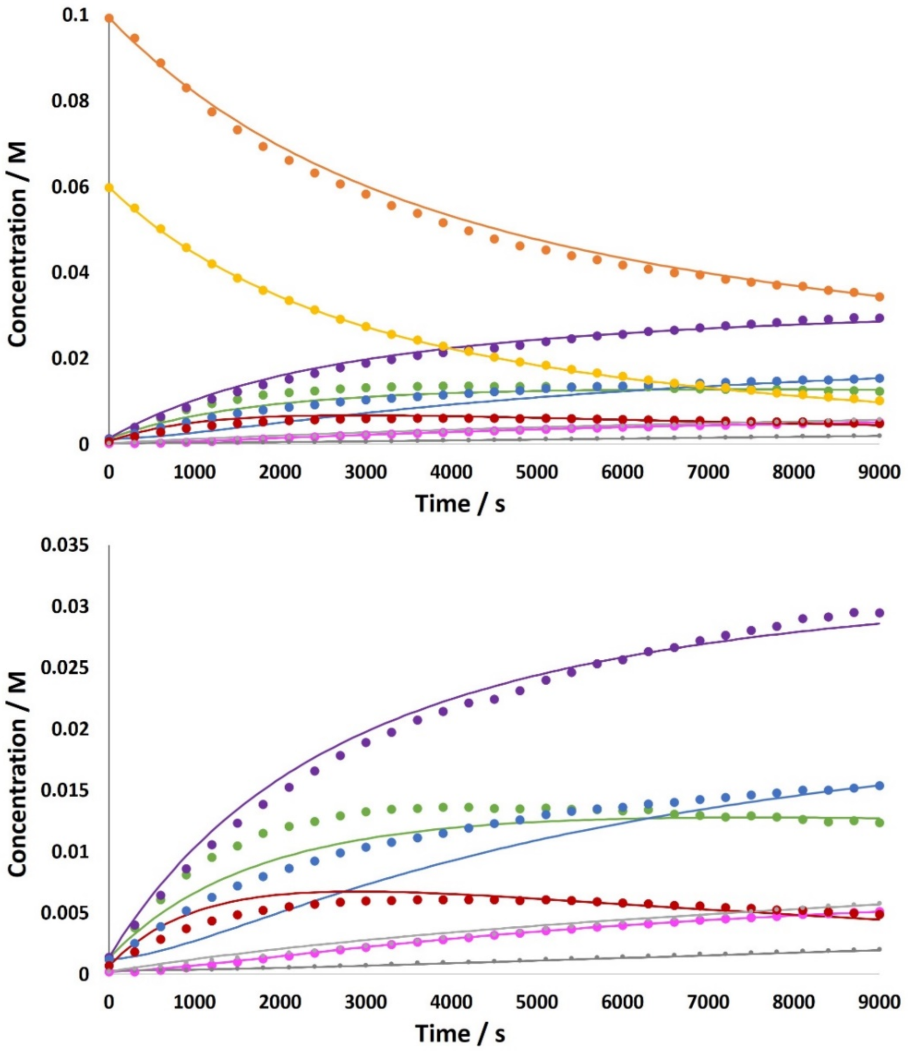

	k_{met}	k_{met-1}	k_{coup1}	k_{coup2}	k_{coup3}	k_{coup4}
Value / s^{-1}; $M^{-1}\cdot s^{-1}$	$1.3\cdot10^{-3}$	$4.6\cdot10^{-8}$	$1.9\cdot10^{-3}$	$5.2\cdot10^{-3}$	$2.4\cdot10^{-3}$	$2.3\cdot10^{-7}$
Std. deviation	$1.6\cdot10^{-5}$	$1.0\cdot10^{-3}$	$4.6\cdot10^{-5}$	$6.3\cdot10^{-5}$	$3.1\cdot10^{-5}$	$9.5\cdot10^{-4}$

Figure S7: Experimental (dots) and least square fit using model I (continuous line) concentration vs time plot of the formed species in the reaction of **1** ($1.07\cdot10^{-1}$ M) with $C_6Cl_2F_3I$ ($7.1\cdot10^{-2}$ M) in THF at 25 ºC. Both graphics represent the same experiment, in the lower one, the concentration vs time lines of the reagents have been omitted to show more clearly the fitting of the products. •**1**; •$C_6Cl_2F_3I$; •$C_6Cl_2F_3$-C_6F_5; •$C_6Cl_2F_3$-$C_6Cl_2F_3$; •C_6F_5-C_6F_5; •**4**; •C_6F_5I; •$C_6Cl_2F_3H$; •C_6F_5H.

- Compound **1** / $C_6Cl_2F_3I$ 0.33 : 1

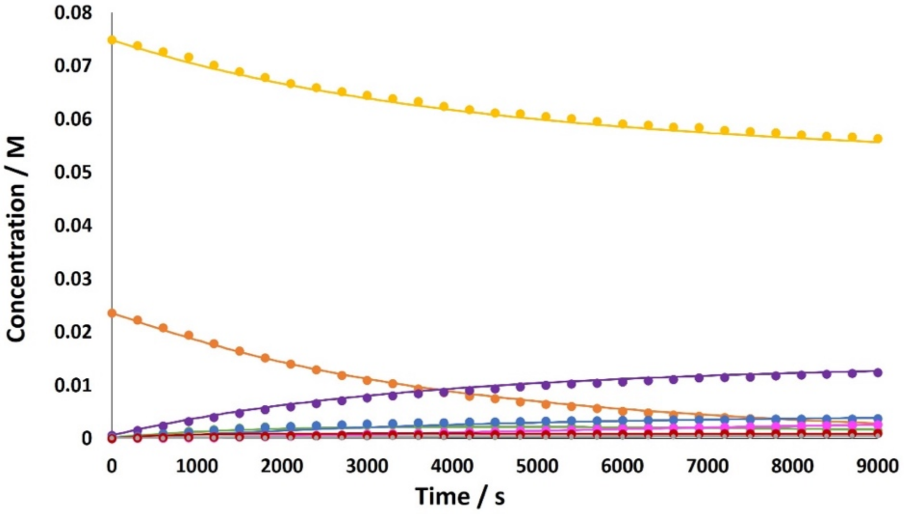

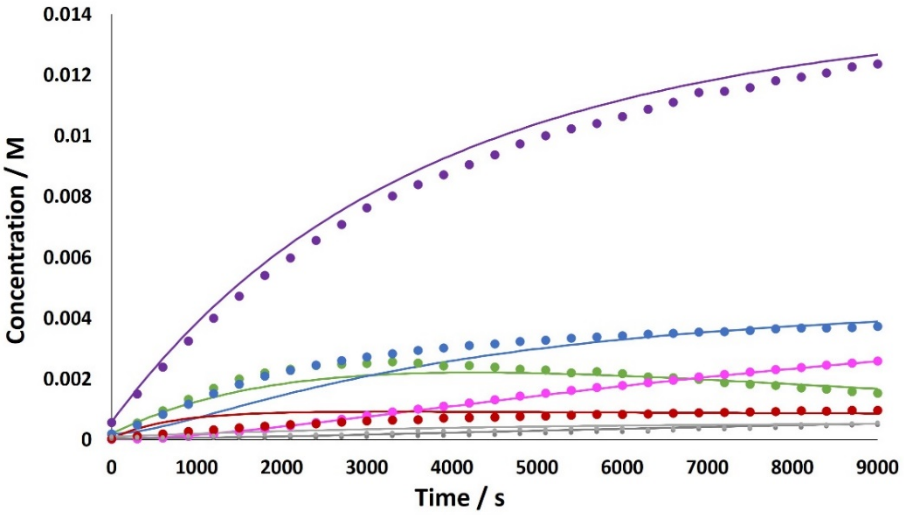

	k_{met}	k_{met-1}	k_{coup1}	k_{coup2}	k_{coup3}	k_{coup4}
Value / s^{-1}; $M^{-1}\cdot s^{-1}$	$8.0\cdot10^{-4}$	$4.5\cdot10^{-8}$	$2.1\cdot10^{-3}$	$5.5\cdot10^{-2}$	$2.5\cdot10^{-3}$	0
Std. deviation	$1.5\cdot10^{-5}$	$2.0\cdot10^{-2}$	$7.6\cdot10^{-5}$	$1.5\cdot10^{-3}$	$4.6\cdot10^{-5}$	$2.2\cdot10^{-2}$

Figure S8: Experimental (dots) and least square fit using model I (continuous line) concentration vs time plot of the formed species in the reaction of **1** ($2.4\cdot10^{-2}$ M) with $C_6Cl_2F_3I$ ($7.1\cdot10^{-2}$ M) in THF at 25 ºC. Both graphics represent the same experiment, in the lower one, the concentration vs time lines of the reagents have been omitted to show more clearly the fitting of the products. •**1**; •$C_6Cl_2F_3I$; •$C_6Cl_2F_3$-C_6F_5; •$C_6Cl_2F_3$-$C_6Cl_2F_3$; •C_6F_5-C_6F_5; •**4**; •C_6F_5I; •$C_6Cl_2F_3H$; •C_6F_5H.

Inconsistencies of the model I:
The best fitting leads to rate constants values that are very different for analogous reactions with C_6F_5 and $C_6Cl_2F_3$, contrarily to the experimental observations.[13] Note also the very poor fitting of the points due to [C_6F_5-C_6F_5] in all the graphics.

Best data fitting using model II pathway a

- Exp. 1. Compound **1** / $C_6Cl_2F_3I$ 1 : 1

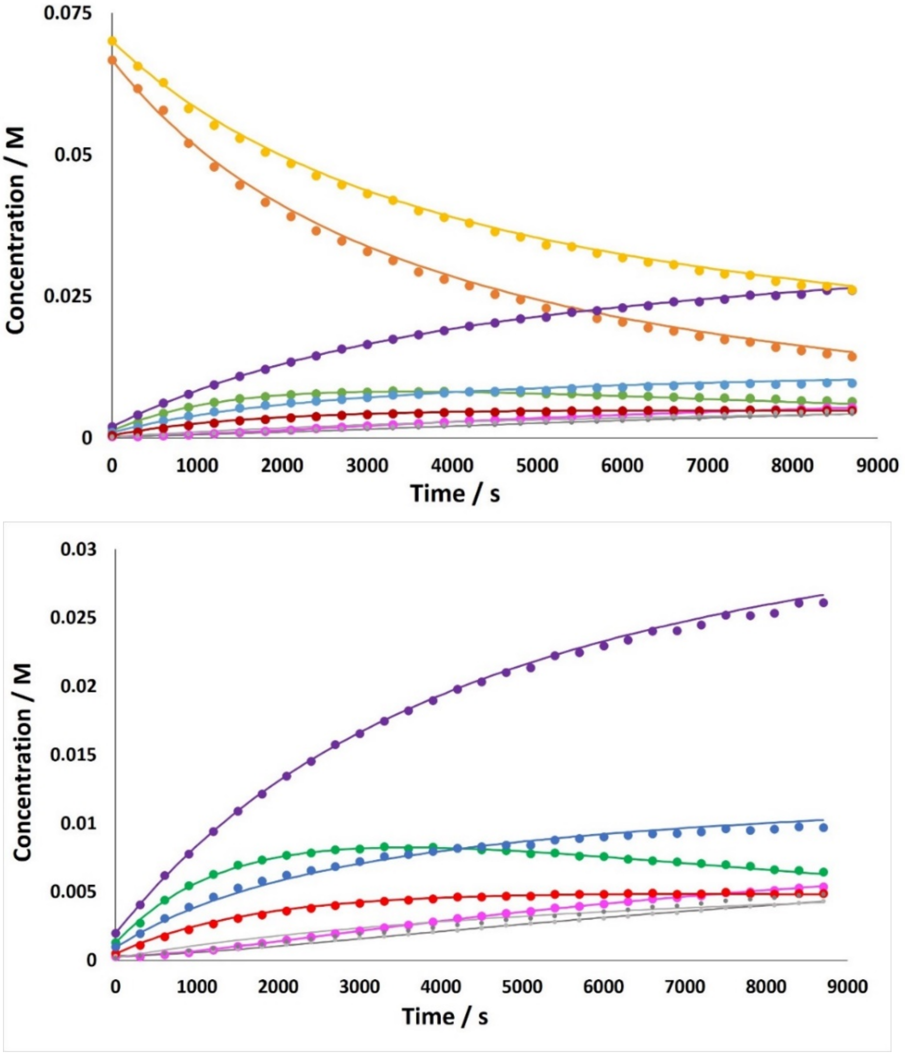

	k_{trans}	k_{retro}	$k_{ad\text{-}ox}$	$k_{x\text{-}i}$	k_{elim}
Value / s^{-1}; $M^{-1}\cdot s^{-1}$	1.7	3.6	$3.4\cdot10^{-3}$	$4.3\cdot10^{-2}$	$1.7\cdot10^{-1}$
Std. deviation	6.0	$1.2\cdot10^{-2}$	$2.5\cdot10^{-5}$	$3.0\cdot10^{-1}$	$6.0\cdot10^{-1}$

Figure S9: Experimental (dots) and least square fit using model II pathway a (continuous line) concentration vs time plot of the formed species in the reaction of **1** ($7.1\cdot10^{-2}$ M) with $C_6Cl_2F_3I$ ($7.1\cdot10^{-2}$ M) in THF at 25 ºC. Both graphics represent the same experiment, in the lower one, the concentration vs time lines of the reagents have been omitted to show more clearly the fitting of the products. •**1**; •$C_6Cl_2F_3I$; •$C_6Cl_2F_3$-C_6F_5; •$C_6Cl_2F_3$-$C_6Cl_2F_3$; •C_6F_5-C_6F_5; •**4**; •C_6F_5I; •$C_6Cl_2F_3H$; •C_6F_5H.

- Exp. 2. Compound **1** / $C_6Cl_2F_3I$ 1.5 : 1

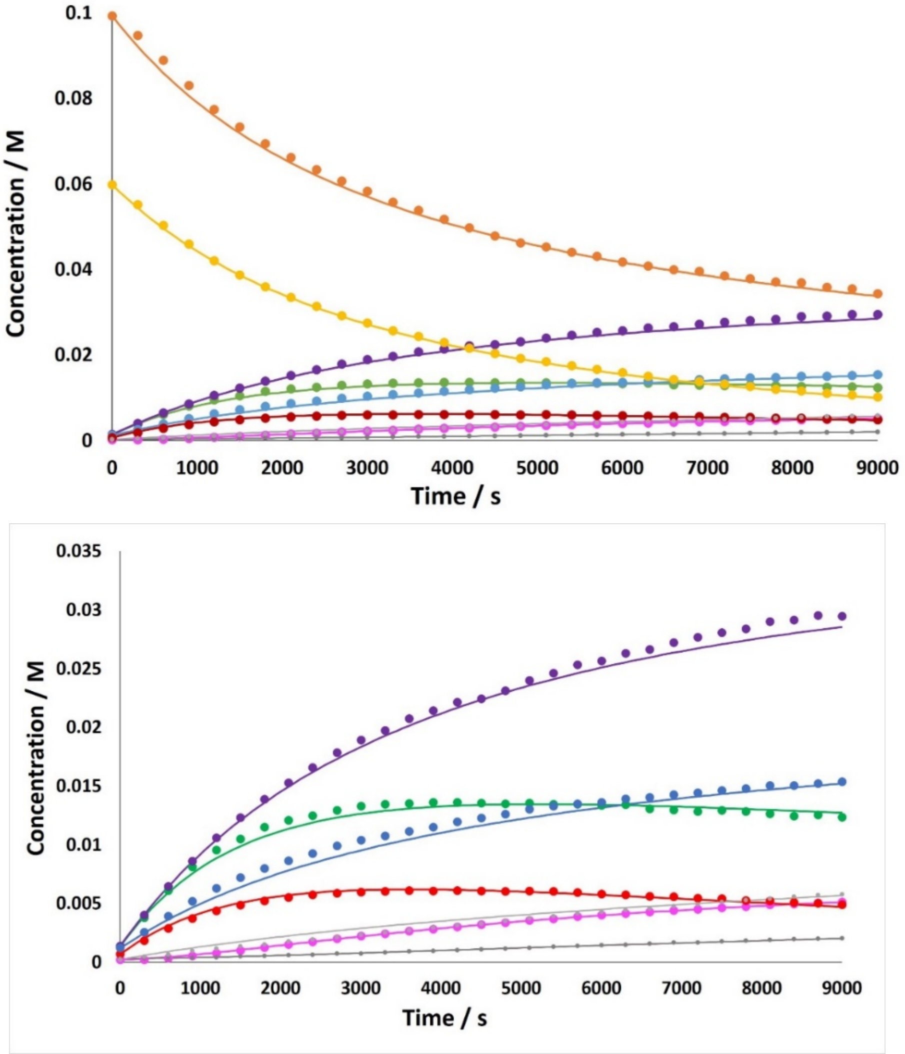

	k_{trans}	k_{retro}	$k_{ad\text{-}ox}$	$k_{x\text{-}i}$	k_{elim}
Value / s^{-1}; $M^{-1}\cdot s^{-1}$	$4.6\cdot10^{-1}$	1.4	$3.9\cdot10^{-3}$	$3.6\cdot10^{-2}$	$9.5\cdot10^{-2}$
Std. deviation	$9.0\cdot10^{-1}$	$5.6\cdot10^{-3}$	$1.7\cdot10^{-5}$	$1.5\cdot10^{-1}$	$1.3\cdot10^{-1}$

Figure S10: Experimental (dots) and least square fit using model II pathway a (continuous line) concentration vs time plot of the formed species in the reaction of **1** ($1.07\cdot10^{-1}$ M) with $C_6Cl_2F_3I$ ($7.1\cdot10^{-2}$ M) in THF at 25 ºC. Both graphics represent the same experiment, in the lower one, the concentration vs time lines of the reagents have been omitted to show more clearly the fitting of the products. •**1**; •$C_6Cl_2F_3I$; •$C_6Cl_2F_3$-C_6F_5; •$C_6Cl_2F_3$-$C_6Cl_2F_3$; •C_6F_5-C_6F_5; •**4**; •C_6F_5I; •$C_6Cl_2F_3H$; •C_6F_5H.

- Compound **1** / $C_6Cl_2F_3I$ 0.33 : 1

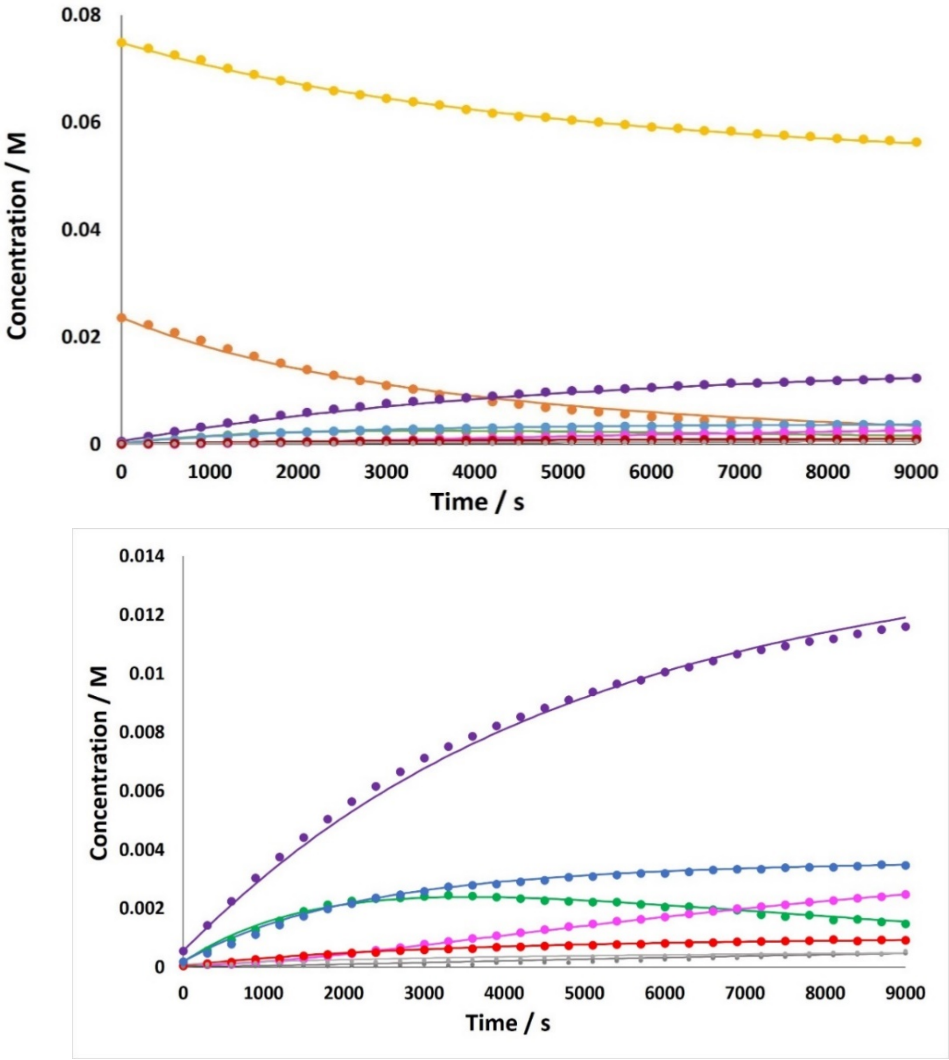

	k_{trans}	k_{retro}	$k_{ad\text{-}ox}$	$k_{x\text{-}i}$	k_{elim}
Value / s^{-1}; $M^{-1}\cdot s^{-1}$	1.2	2.1	$2.9\cdot10^{-3}$	$3.2\cdot10^{-3}$	$4.4\cdot10^{-2}$
Std. deviation	1.7	1.2	$2.8\cdot10^{-5}$	1.3	$6.3\cdot10^{-2}$

Figure S11: Experimental (dots) and least square fit using model II pathway a (continuous line) concentration vs time plot of the formed species in the reaction of **1** ($2.4\cdot10^{-2}$ M) with $C_6Cl_2F_3I$ ($7.1\cdot10^{-2}$ M) in THF at 25 ºC. Both graphics represent the same experiment, in the lower one, the concentration vs time lines of the reagents have been omitted to show more clearly the fitting of the products. •**1**; •$C_6Cl_2F_3I$; •$C_6Cl_2F_3$-C_6F_5; •$C_6Cl_2F_3$-$C_6Cl_2F_3$; •C_6F_5-C_6F_5; •**4**; •C_6F_5I; •$C_6Cl_2F_3H$; •C_6F_5H.

Data fitting using <u>model II pathway b</u>

- Exp. 1. Compound **1** / $C_6Cl_2F_3I$ 1 : 1

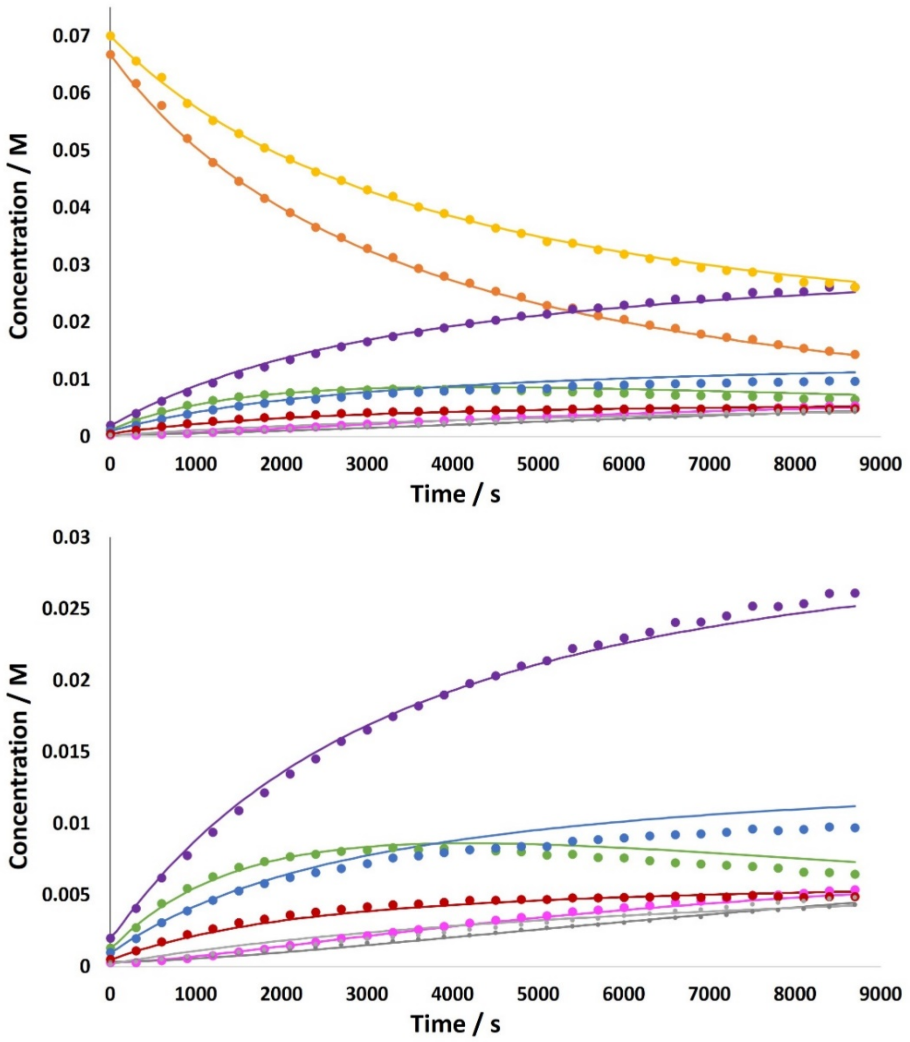

	k_{trans}	k_{retro}	$k_{ad\text{-}ox}$	$k_{x\text{-}i}$	k_{elim}
Value / s^{-1}; $M^{-1}\cdot s^{-1}$	$4.7\cdot10^{-1}$	$4.2\cdot10^{-6}$	$3.7\cdot10^{-3}$	$5.9\cdot10^{-3}$	$1.4\cdot10^{15}$

Figure S12: Experimental (dots) and least square fit using <u>model II pathway b</u> (continuous line) concentration vs time plot of the formed species in the reaction of **1** ($7.1\cdot10^{-2}$ M) with $C_6Cl_2F_3I$ ($7.1\cdot10^{-2}$ M) in THF at 25 ºC. Both graphics represent the same experiment, in the lower one, the concentration vs time lines of the reagents have been omitted to show more clearly the fitting of the products. •**1**; •$C_6Cl_2F_3I$; •$C_6Cl_2F_3$-C_6F_5; •$C_6Cl_2F_3$-$C_6Cl_2F_3$; •C_6F_5-C_6F_5; •**4**; •C_6F_5I; •$C_6Cl_2F_3H$; •C_6F_5H.

- Exp. 2. Compound **1** / $C_6Cl_2F_3I$ 1.5 : 1

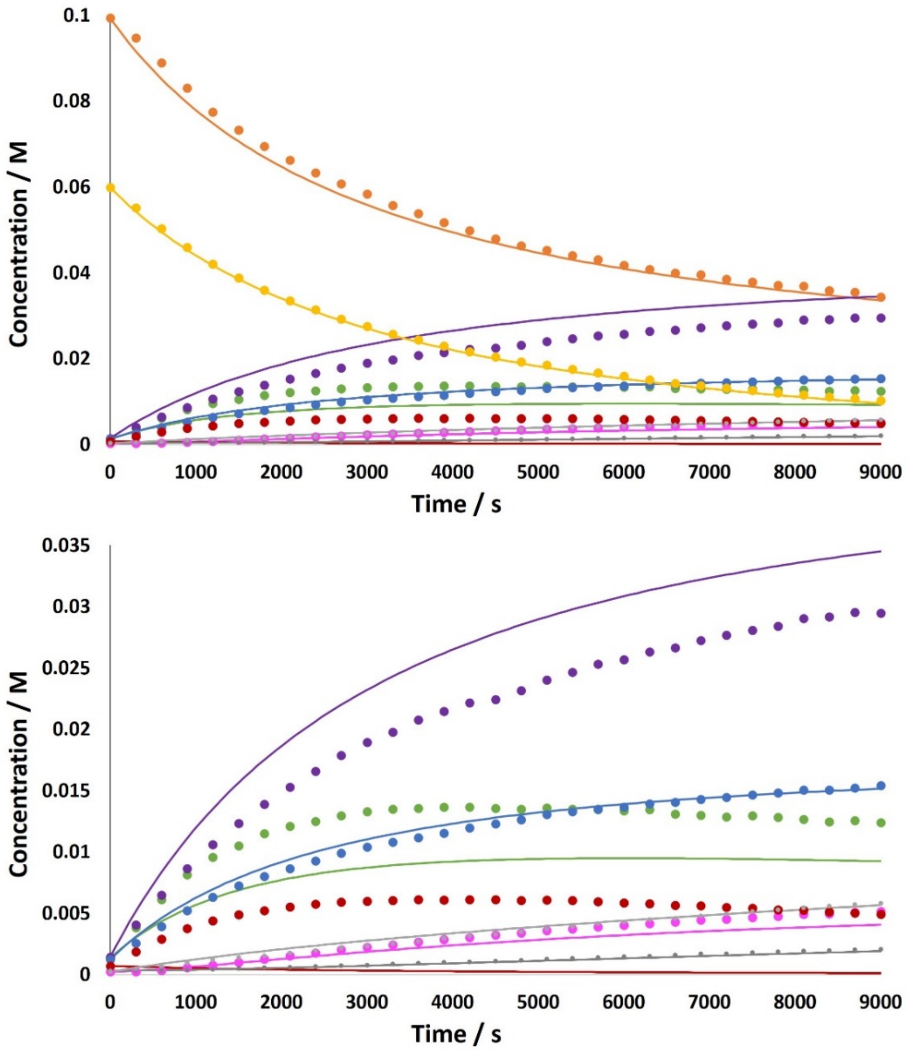

	k_{trans}	k_{retro}	$k_{ad\text{-}ox}$	$k_{x\text{-}i}$	k_{elim}
Value / s^{-1}; $M^{-1}\cdot s^{-1}$	$3.4\cdot10^{4}$	$2.0\cdot10^{-9}$	$3.4\cdot10^{-3}$	$1.4\cdot10^{-15}$	$2.8\cdot10^{4}$

Figure S13: Experimental (dots) and least square fit using model II pathway b (continuous line) concentration vs time plot of the formed species in the reaction of **1** ($1.07\cdot10^{-1}$ M) with $C_6Cl_2F_3I$ ($7.1\cdot10^{-2}$ M) in THF at 25 ºC. Both graphics represent the same experiment, in the lower one, the concentration vs time lines of the reagents have been omitted to show more clearly the fitting of the products. •**1**; •$C_6Cl_2F_3I$; •$C_6Cl_2F_3$-C_6F_5; •$C_6Cl_2F_3$-$C_6Cl_2F_3$; •C_6F_5-C_6F_5; •**4**; •C_6F_5I; •$C_6Cl_2F_3H$; •C_6F_5H.

Compound **1** / $C_6Cl_2F_3I$ 0.33 : 1

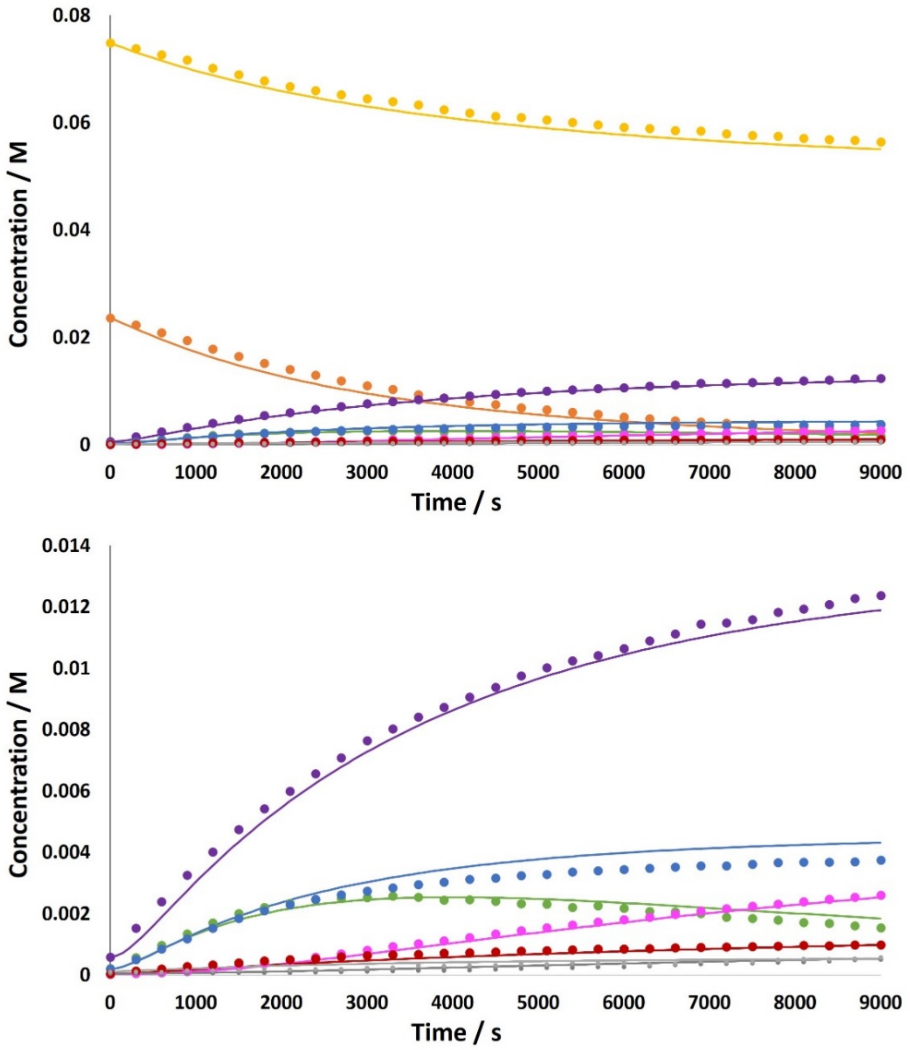

	k_{trans}	k_{retro}	$k_{ad\text{-}ox}$	$k_{x\text{-}i}$	k_{elim}
Value / s^{-1}; $M^{-1}\cdot s^{-1}$	$1.6\cdot10^{-1}$	$1.7\cdot10^{-5}$	$3.5\cdot10^{-3}$	$1.4\cdot10^{-4}$	$6.2\cdot10^{16}$

Figure S14: Experimental (dots) and least square fit using model II pathway b (continuous line) concentration vs time plot of the formed species in the reaction of **1** ($2.4\cdot10^{-2}$ M) with $C_6Cl_2F_3I$ ($7.1\cdot10^{-2}$ M) in THF at 25 ºC. Both graphics represent the same experiment, in the lower one, the concentration vs time lines of the reagents have been omitted to show more clearly the fitting of the products. •**1**; •$C_6Cl_2F_3I$; •$C_6Cl_2F_3$-C_6F_5; •$C_6Cl_2F_3$-$C_6Cl_2F_3$; •C_6F_5-C_6F_5; •**4**; •C_6F_5I; •$C_6Cl_2F_3H$; •C_6F_5H.

Inconsistencies of the model II pathway b:
Using this model, the correlation matrix could not be calculated. Also note that kinetic constant values between different experiments differ significatively

S8. NMR spectra

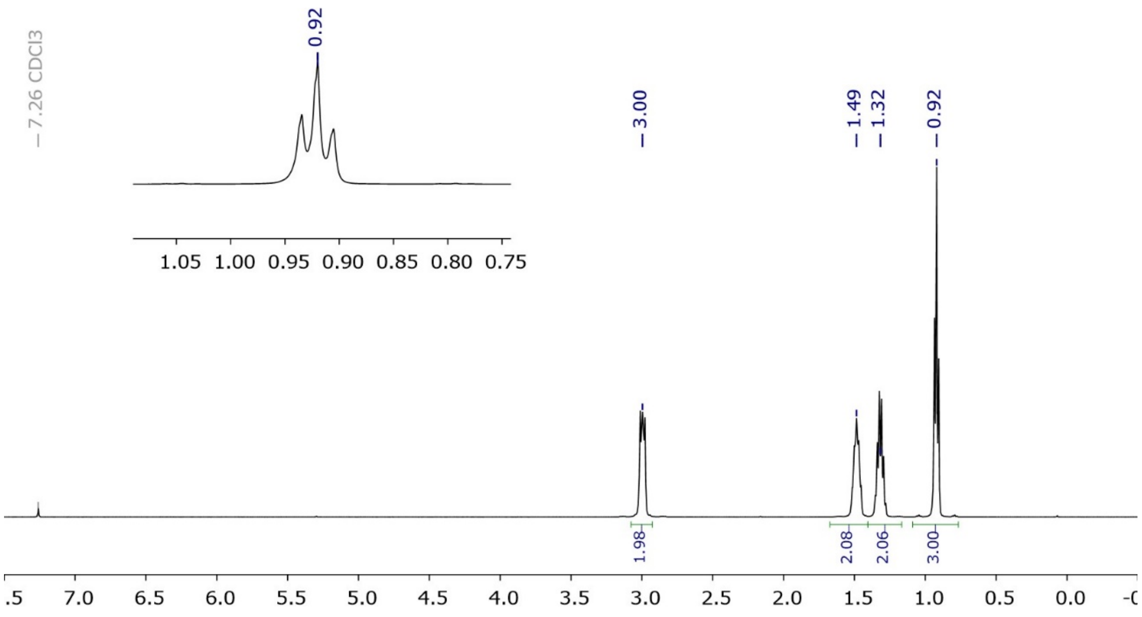

Spectrum S1: ^{1}H NMR of $(NBu_4)[Cu(C_6F_5)_2]$.

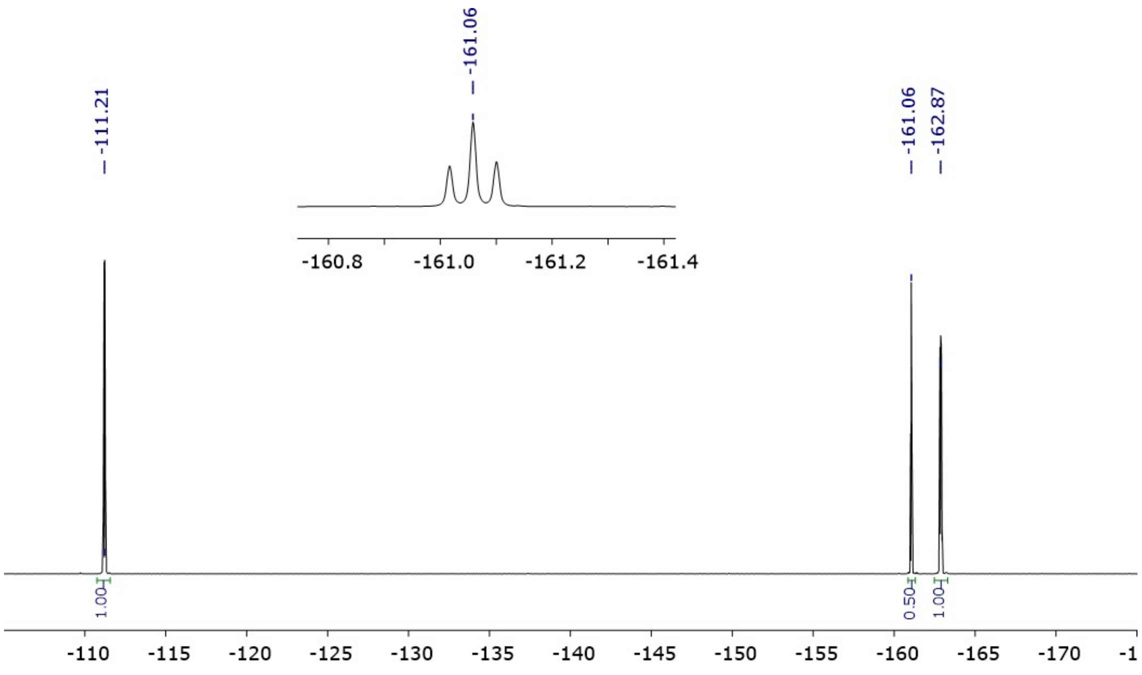

Spectrum S2: ^{19}F NMR of $(NBu_4)[Cu(C_6F_5)_2]$.

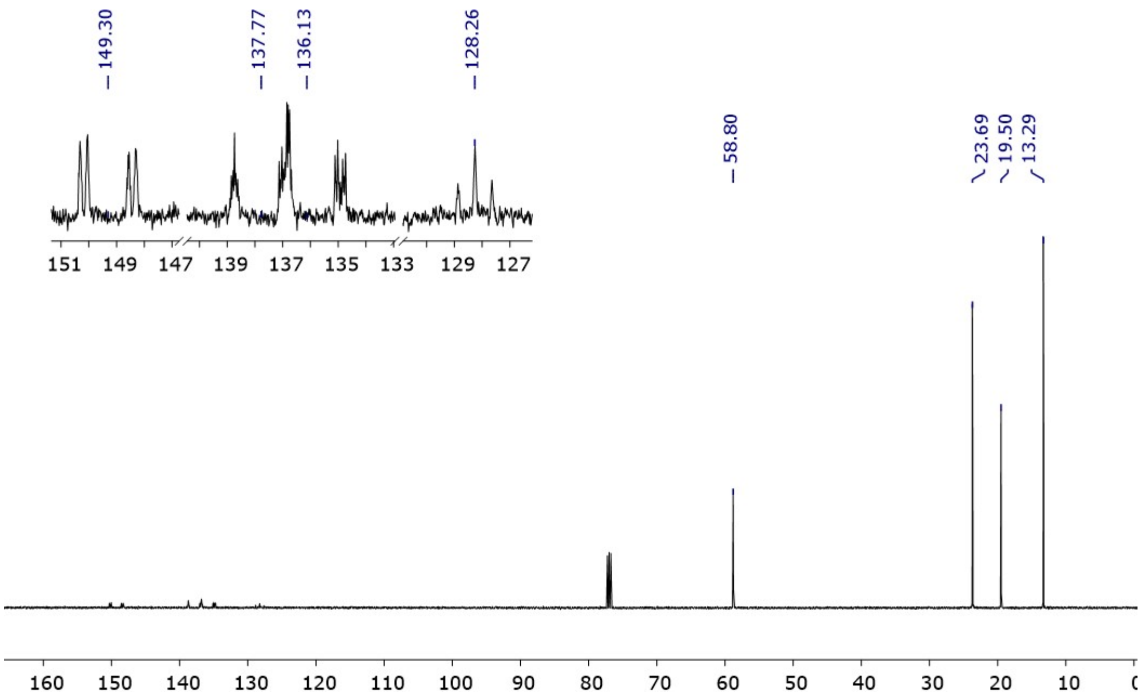

Spectrum S3: $^{13}C\{^{1}H\}$ NMR of $(NBu_4)[Cu(C_6F_5)_2]$.

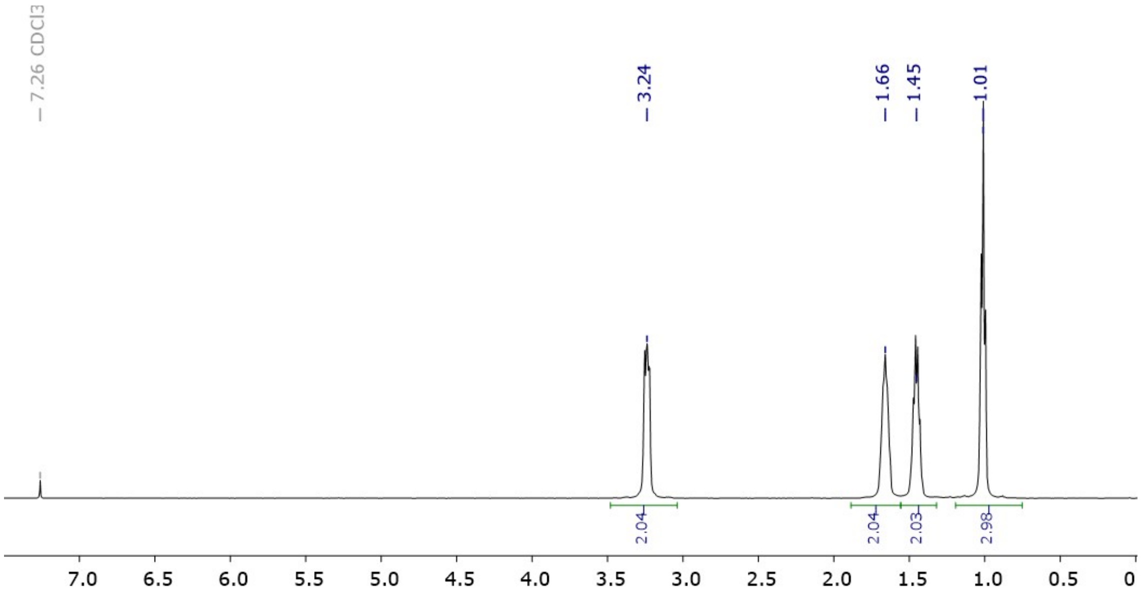

Spectrum S4: ^{1}H NMR of $(NBu_4)[CuCl_2]$.

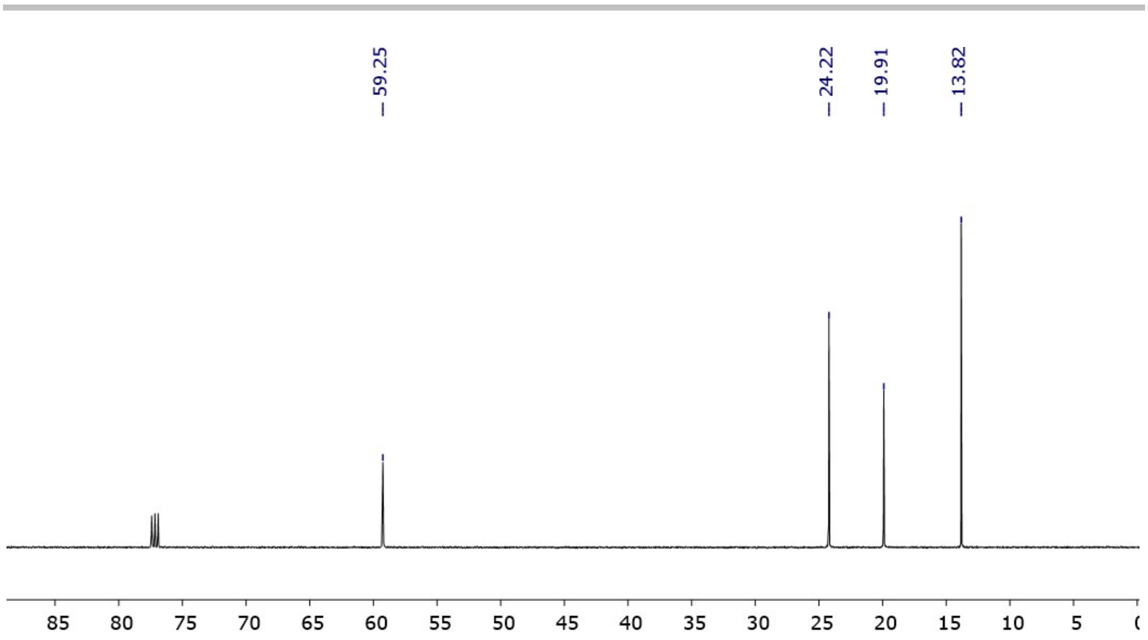

Spectrum S5: $^{13}C\{^{1}H\}$ NMR of $(NBu_4)[CuCl_2]$.

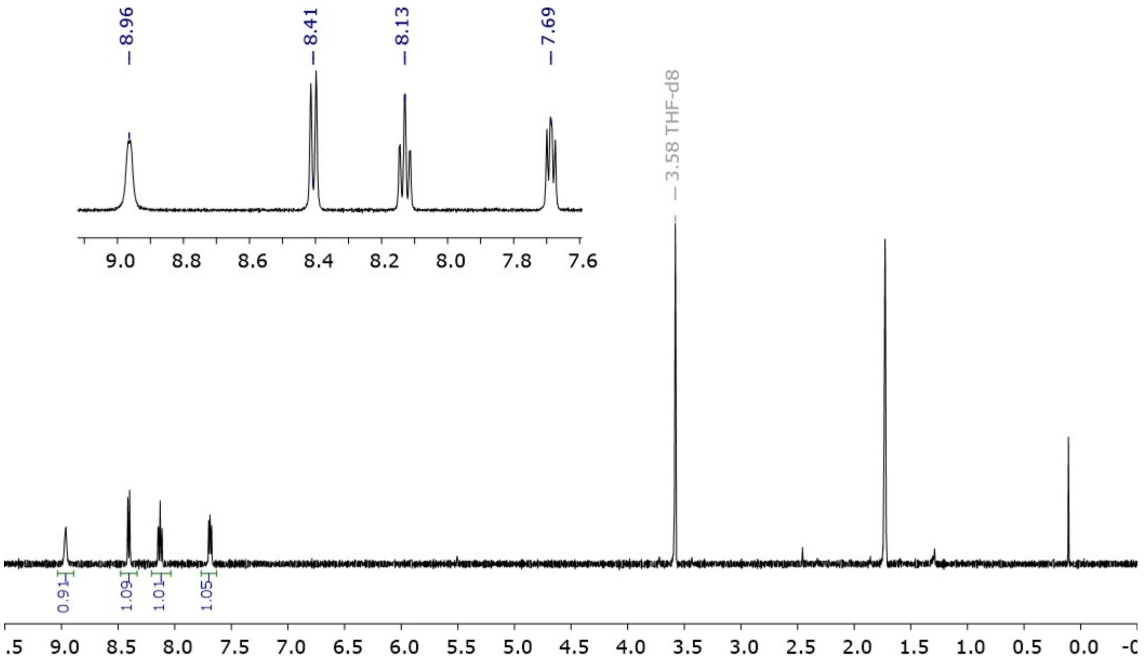

Spectrum S6: ^{1}H NMR of $[Cu(bipy)(C_6Cl_2F_3)]$.

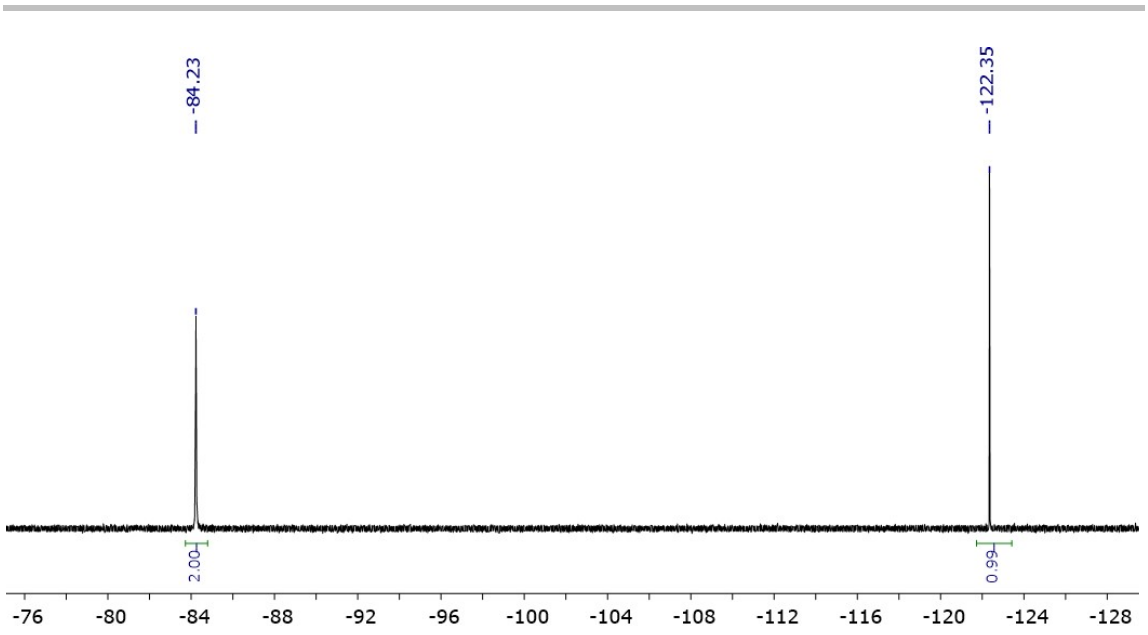

Spectrum S7: ^{19}F NMR of [Cu(bipy)($C_6Cl_2F_3$)].

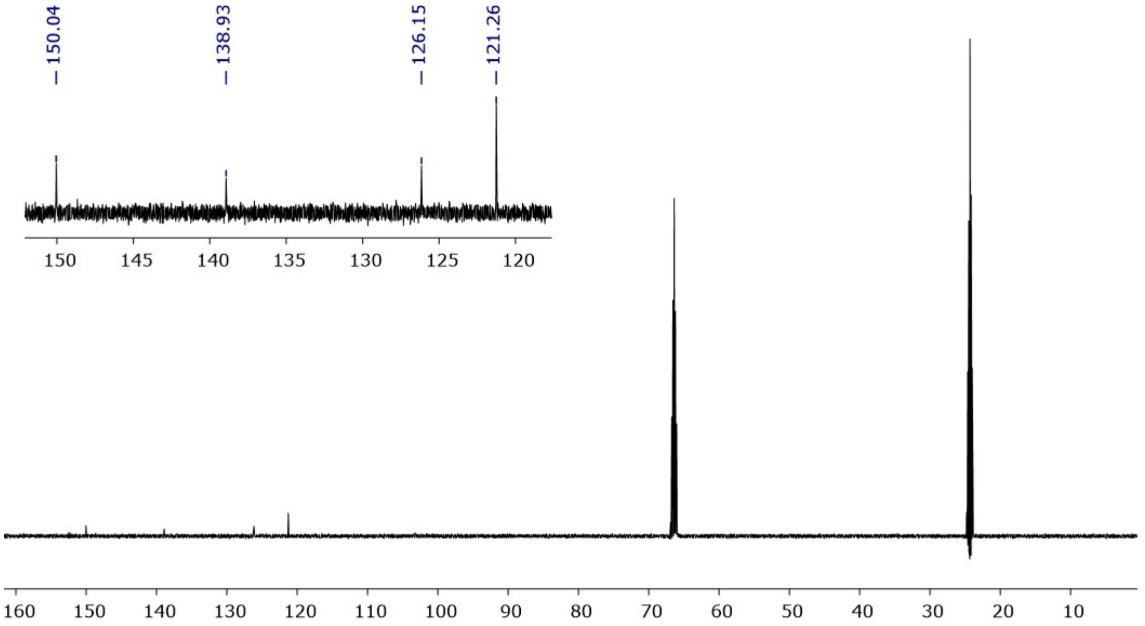

Spectrum S8: $^{13}C\{^1H\}$ NMR of [Cu(bipy)($C_6Cl_2F_3$)].

S9. Concentration/time data from fitted kinetic experiments

Reactions of compound 1 and aryl iodide $C_6Cl_2F_3I$

- Compound **1** / $C_6Cl_2F_3I$ 1 : 1

Table S7: Concentration vs time data fed to program COPASI to obtain kinetic parameters of the reaction of **1** ($7.1 \cdot 10^{-2}$M) with $C_6Cl_2F_3I$ ($7.1 \cdot 10^{-2}$M) in THF at 25 ºC.

Time / s	[**4**] / 10^{-2} M	[**1**] / 10^{-2} M	[$C_6Cl_2F_3$-$C_6Cl_2F_3$] / 10^{-2} M	[C_6F_5-C_6F_5] / 10^{-2} M	[$C_6Cl_2F_3$-$C_6Cl_2F_3$] / 10^{-2} M	[$C_6Cl_2F_3I$] / 10^{-2} M	[C_6F_5I] / 10^{-2} M	[$C_6Cl_2F_3H$] / 10^{-2} M	[C_6F_5H] / 10^{-2} M
0	0.130	6.674	0.200	0.098	0.027	7.006	0.049	0.031	0.016
300	0.273	6.170	0.407	0.195	0.028	6.562	0.110	0.040	0.034
600	0.441	5.784	0.619	0.306	0.042	6.275	0.173	0.049	0.050
900	0.546	5.208	0.777	0.388	0.057	5.819	0.223	0.059	0.071
1200	0.629	4.787	0.940	0.464	0.076	5.520	0.265	0.074	0.087
1500	0.695	4.465	1.089	0.528	0.102	5.294	0.306	0.087	0.102
1800	0.734	4.163	1.215	0.579	0.123	5.044	0.333	0.100	0.118
2100	0.767	3.913	1.345	0.622	0.146	4.846	0.360	0.115	0.138
2400	0.785	3.659	1.453	0.656	0.169	4.627	0.381	0.132	0.156
2700	0.806	3.480	1.574	0.685	0.194	4.475	0.400	0.150	0.174
3000	0.813	3.289	1.654	0.721	0.216	4.313	0.417	0.164	0.193
3300	0.831	3.132	1.747	0.759	0.239	4.196	0.431	0.176	0.203
3600	0.816	2.936	1.822	0.773	0.258	4.010	0.439	0.190	0.219
3900	0.826	2.805	1.898	0.797	0.280	3.899	0.450	0.207	0.238
4200	0.818	2.685	1.979	0.818	0.305	3.793	0.462	0.219	0.258
4500	0.808	2.536	2.033	0.828	0.323	3.643	0.462	0.236	0.272
4800	0.801	2.440	2.102	0.841	0.343	3.549	0.469	0.256	0.294
5100	0.779	2.294	2.137	0.840	0.356	3.407	0.469	0.269	0.308
5400	0.784	2.247	2.224	0.877	0.383	3.379	0.482	0.282	0.323
5700	0.762	2.113	2.248	0.888	0.395	3.265	0.482	0.292	0.335
6000	0.759	2.045	2.297	0.900	0.412	3.188	0.483	0.307	0.355
6300	0.738	1.947	2.339	0.913	0.428	3.107	0.487	0.320	0.369
6600	0.726	1.892	2.404	0.923	0.449	3.058	0.491	0.342	0.389
6900	0.718	1.793	2.408	0.927	0.457	2.953	0.483	0.346	0.398
7200	0.709	1.737	2.450	0.939	0.471	2.900	0.487	0.362	0.415
7500	0.700	1.696	2.521	0.962	0.494	2.876	0.498	0.383	0.434
7800	0.687	1.604	2.518	0.950	0.500	2.769	0.488	0.394	0.447
8100	0.661	1.542	2.535	0.958	0.511	2.696	0.484	0.402	0.457
8400	0.657	1.491	2.609	0.975	0.529	2.684	0.488	0.417	0.474
8700	0.645	1.436	2.611	0.970	0.537	2.614	0.490	0.431	0.482

Compound **1** / $C_6Cl_2F_3I$ 1.5 : 1

Table S8: Concentration vs time data fed to program COPASI to obtain kinetic parameters of the reaction of **1** ($1.07\cdot10^{-1}$M) with $C_6Cl_2F_3I$ ($7.1\cdot10^{-2}$M) in THF at 25 ºC.

Time / s	[**4**] / 10^{-2} M	[**1**] / 10^{-2} M	[$C_6Cl_2F_3$-$C_6Cl_2F_3$] / 10^{-2} M	[C_6F_5-C_6F_5] / 10^{-2} M	[$C_6Cl_2F_3$-$C_6Cl_2F_3$] / 10^{-2} M	[$C_6Cl_2F_3I$] / 10^{-2} M	[C_6F_5I] / 10^{-2} M	[$C_6Cl_2F_3H$] / 10^{-2} M	[C_6F_5H] / 10^{-2} M
0	0.132	9.935	0.140	0.122	0.021	5.987	0.070	0.031	0.022
300	0.380	9.471	0.403	0.255	0.023	5.513	0.185	0.031	0.048
600	0.611	8.894	0.647	0.392	0.034	5.030	0.289	0.034	0.068
900	0.813	8.306	0.862	0.519	0.054	4.590	0.373	0.038	0.097
1200	0.956	7.746	1.059	0.630	0.076	4.207	0.437	0.041	0.117
1500	1.050	7.330	1.233	0.722	0.100	3.877	0.486	0.049	0.137
1800	1.150	6.944	1.387	0.799	0.126	3.593	0.522	0.055	0.150
2100	1.209	6.620	1.527	0.864	0.152	3.351	0.552	0.059	0.177
2400	1.247	6.331	1.658	0.926	0.174	3.141	0.574	0.065	0.194
2700	1.296	6.070	1.785	0.990	0.200	2.921	0.589	0.073	0.213
3000	1.329	5.831	1.893	1.038	0.222	2.746	0.598	0.075	0.232
3300	1.347	5.572	1.975	1.077	0.243	2.565	0.603	0.084	0.252
3600	1.353	5.382	2.075	1.115	0.265	2.434	0.609	0.093	0.273
3900	1.363	5.170	2.145	1.150	0.283	2.292	0.608	0.097	0.296
4200	1.363	4.978	2.215	1.195	0.302	2.164	0.610	0.103	0.313
4500	1.355	4.787	2.243	1.229	0.320	2.041	0.609	0.109	0.328
4800	1.349	4.626	2.313	1.261	0.337	1.922	0.604	0.114	0.349
5100	1.357	4.528	2.401	1.303	0.357	1.848	0.608	0.124	0.372
5400	1.347	4.400	2.464	1.329	0.371	1.749	0.603	0.131	0.391
5700	1.345	4.305	2.532	1.349	0.388	1.664	0.594	0.138	0.415
6000	1.335	4.178	2.566	1.364	0.401	1.590	0.583	0.144	0.428
6300	1.343	4.082	2.632	1.390	0.415	1.504	0.575	0.150	0.446
6600	1.308	3.997	2.665	1.403	0.427	1.426	0.563	0.159	0.457
6900	1.298	3.955	2.724	1.428	0.443	1.378	0.559	0.167	0.477
7200	1.285	3.852	2.768	1.443	0.452	1.319	0.546	0.168	0.494
7500	1.295	3.783	2.807	1.463	0.464	1.263	0.541	0.178	0.513
7800	1.283	3.713	2.838	1.479	0.474	1.203	0.527	0.184	0.529
8100	1.265	3.691	2.902	1.504	0.490	1.159	0.523	0.192	0.544
8400	1.244	3.589	2.915	1.505	0.495	1.105	0.509	0.196	0.559
8700	1.255	3.546	2.952	1.521	0.506	1.071	0.502	0.200	0.571
9000	1.237	3.436	2.948	1.540	0.514	1.023	0.490	0.205	0.579
9300	1.212	3.372	2.968	1.568	0.523	0.986	0.481	0.207	0.583
9600	1.214	3.322	3.008	1.570	0.528	0.949	0.471	0.214	0.602
9900	1.197	3.290	3.028	1.575	0.539	0.911	0.461	0.220	0.619
10200	1.179	3.240	3.062	1.584	0.547	0.876	0.449	0.226	0.629
10500	1.175	3.211	3.101	1.603	0.554	0.842	0.444	0.233	0.646
10800	1.162	3.116	3.059	1.608	0.557	0.813	0.433	0.234	0.642
11100	1.164	3.063	3.072	1.636	0.563	0.783	0.422	0.237	0.647
11400	1.145	3.041	3.112	1.635	0.572	0.757	0.415	0.243	0.666
11700	1.134	3.012	3.154	1.646	0.579	0.730	0.408	0.249	0.680
12000	1.119	2.965	3.160	1.659	0.584	0.704	0.398	0.253	0.686

12300	1.104	2.906	3.147	1.672	0.588	0.683	0.390	0.254	0.683
12600	1.119	2.861	3.161	1.692	0.594	0.664	0.386	0.257	0.694
12900	1.100	2.848	3.190	1.686	0.598	0.641	0.372	0.267	0.712
13200	1.095	2.830	3.231	1.696	0.605	0.622	0.369	0.270	0.726
13500	1.075	2.778	3.211	1.708	0.607	0.600	0.357	0.273	0.718
13800	1.072	2.724	3.213	1.727	0.609	0.579	0.349	0.273	0.722
14100	1.069	2.693	3.228	1.730	0.614	0.561	0.343	0.279	0.732

Compound **1** / $C_6Cl_2F_3I$ 0.33 : 1

Table S9: Concentration vs time data fed to program COPASI to obtain kinetic parameters of the reaction of **1** ($2.4 \cdot 10^{-2}$M) with $C_6Cl_2F_3I$ ($7.1 \cdot 10^{-2}$M) in THF at 25 ºC.

Time / s	[**4**] / 10^{-2} M	[**1**] / 10^{-2} M	[$C_6Cl_2F_3$-$C_6Cl_2F_3$] / 10^{-2} M	[C_6F_5-C_6F_5] / 10^{-2} M	[$C_6Cl_2F_3$-$C_6Cl_2F_3$] / 10^{-2} M	[$C_6Cl_2F_3I$] / 10^{-2} M	[C_6F_5I] / 10^{-2} M	[$C_6Cl_2F_3H$] / 10^{-2} M	[C_6F_5H] / 10^{-2} M
0	0.020	2.359	0.058	0.020	0.002	7.486	0.004	0.005	0.010
300	0.056	2.230	0.152	0.050	0.004	7.383	0.013	0.004	0.010
600	0.097	2.086	0.239	0.084	0.007	7.265	0.020	0.004	0.009
900	0.134	1.944	0.325	0.118	0.012	7.167	0.028	0.005	0.014
1200	0.170	1.784	0.401	0.153	0.019	7.011	0.033	0.006	0.014
1500	0.201	1.644	0.474	0.184	0.027	6.895	0.040	0.007	0.016
1800	0.221	1.519	0.541	0.210	0.038	6.781	0.046	0.007	0.019
2100	0.234	1.399	0.599	0.229	0.047	6.669	0.051	0.008	0.017
2400	0.245	1.293	0.656	0.247	0.058	6.596	0.053	0.009	0.018
2700	0.249	1.188	0.708	0.261	0.069	6.520	0.059	0.010	0.018
3000	0.252	1.099	0.763	0.274	0.081	6.446	0.063	0.011	0.022
3300	0.257	1.032	0.803	0.284	0.093	6.389	0.066	0.013	0.023
3600	0.253	0.932	0.840	0.295	0.102	6.329	0.067	0.014	0.030
3900	0.244	0.864	0.872	0.302	0.111	6.240	0.072	0.015	0.029
4200	0.246	0.794	0.906	0.311	0.122	6.179	0.073	0.016	0.030
4500	0.240	0.748	0.938	0.316	0.133	6.118	0.075	0.018	0.031
4800	0.233	0.691	0.975	0.324	0.144	6.099	0.078	0.020	0.035
5100	0.231	0.647	1.001	0.328	0.154	6.048	0.080	0.023	0.034
5400	0.221	0.603	1.024	0.335	0.163	6.008	0.082	0.024	0.038
5700	0.225	0.570	1.042	0.339	0.173	5.960	0.084	0.026	0.037
6000	0.218	0.514	1.064	0.343	0.180	5.915	0.085	0.027	0.038
6300	0.207	0.483	1.089	0.348	0.189	5.891	0.086	0.029	0.034
6600	0.205	0.445	1.111	0.351	0.197	5.850	0.088	0.033	0.037
6900	0.200	0.425	1.144	0.355	0.208	5.842	0.090	0.036	0.044
7200	0.188	0.401	1.148	0.356	0.216	5.789	0.091	0.037	0.042
7500	0.184	0.376	1.159	0.361	0.224	5.764	0.093	0.038	0.042
7800	0.179	0.356	1.182	0.366	0.231	5.744	0.094	0.040	0.048
8100	0.171	0.330	1.194	0.368	0.238	5.706	0.097	0.042	0.051
8400	0.168	0.302	1.207	0.368	0.246	5.686	0.095	0.045	0.050
8700	0.160	0.295	1.227	0.370	0.253	5.668	0.098	0.049	0.053
9000	0.154	0.271	1.237	0.374	0.260	5.638	0.098	0.050	0.057
9300	0.146	0.251	1.245	0.376	0.266	5.638	0.101	0.051	0.055

9600	0.140	0.231	1.246	0.376	0.270	5.589	0.100	0.053	0.056
9900	0.138	0.219	1.258	0.379	0.276	5.588	0.101	0.056	0.058
10200	0.136	0.186	1.274	0.378	0.280	5.564	0.102	0.059	0.061
10500	0.129	0.190	1.290	0.380	0.290	5.589	0.102	0.062	0.063
10800	0.123	0.162	1.291	0.380	0.294	5.558	0.103	0.063	0.061
11100	0.118	0.159	1.297	0.381	0.298	5.560	0.104	0.063	0.063
11400	0.113	0.152	1.299	0.385	0.302	5.518	0.104	0.066	0.065
11700	0.106	0.133	1.305	0.385	0.306	5.525	0.106	0.068	0.065
12000	0.103	0.126	1.319	0.388	0.312	5.523	0.107	0.071	0.068
12300	0.101	0.134	1.329	0.388	0.320	5.506	0.107	0.073	0.065
12600	0.096	0.127	1.332	0.385	0.324	5.493	0.108	0.076	0.060
12900	0.086	0.103	1.336	0.388	0.325	5.477	0.108	0.076	0.069
13200	0.083	0.093	1.343	0.389	0.328	5.448	0.108	0.078	0.069
13500	0.084	0.084	1.340	0.391	0.331	5.459	0.110	0.081	0.069
13800	0.079	0.076	1.344	0.389	0.334	5.436	0.111	0.082	0.070
14100	0.077	0.079	1.354	0.392	0.339	5.425	0.111	0.083	0.071

Compound **1** / PhI 1 : 6

Table S10: Concentration vs time data used as input in program COPASI to obtain kinetic parameters of the reaction of **1** ($2.4\cdot10^{-2}$M) with PhI ($4.3\cdot10^{-1}$M) in THF at 50 ºC.

Time / s	[**1**] / 10^{-2} M	[C_6F_5-C_6H_5] / 10^{-2} M	[C_6F_5H] / 10^{-2} M
86	7.145	0.019	0.042
404	7.037	0.210	0.019
712	6.620	0.395	0.106
1030	6.497	0.518	0.128
1346	6.236	0.726	0.208
1656	6.008	0.865	0.233
1968	5.790	1.115	0.273
2282	5.750	1.130	0.284
2590	5.488	1.297	0.305
2897	5.291	1.425	0.356
3206	5.078	1.720	0.364
3513	4.969	1.794	0.377
3820	4.699	1.987	0.409
4128	4.619	2.002	0.457
4435	4.593	2.161	0.419
4745	4.434	2.226	0.484
5061	4.363	2.402	0.454
5372	4.155	2.477	0.488
5679	4.010	2.608	0.508
5987	3.832	2.784	0.532
6300	3.894	2.871	0.489
6608	3.692	3.026	0.505
6919	3.535	3.015	0.562

7237	3.428	3.104	0.621
7548	3.331	3.281	0.586
7855	3.185	3.360	0.606
8163	3.146	3.369	0.603
8480	3.029	3.540	0.544
8788	2.919	3.658	0.616
9101	2.880	3.565	0.617
9418	2.790	3.701	0.644
9728	2.658	3.842	0.633
10035	2.529	3.895	0.671
10348	2.445	4.055	0.678
10672	2.346	4.086	0.682
10980	2.338	4.110	0.644
11294	2.186	4.209	0.744
11610	2.181	4.269	0.680
11926	2.080	4.329	0.686
12239	2.059	4.393	0.658
12560	1.990	4.359	0.729
12872	1.917	4.584	0.702
13181	1.852	4.560	0.716
13489	1.759	4.603	0.698
13797	1.711	4.564	0.773
14105	1.708	4.624	0.762
14421	1.652	4.658	0.733
14735	1.502	4.787	0.818
15043	1.558	4.876	0.751
15357	1.443	4.945	0.804
15665	1.388	4.855	0.790
15979	1.408	5.050	0.808
16296	1.356	5.026	0.776
16604	1.308	5.052	0.777
16920	1.244	5.036	0.804
17237	1.286	5.098	0.730
17550	1.104	5.171	0.834
17865	1.093	5.286	0.777
18177	1.100	5.211	0.771
18493	1.033	5.113	0.820
18800	0.985	5.136	0.867

S10. X-ray crystallography: Structure of $[Cu(C_6F_5)_2](NBu_4)$

For the compound $C_{28}H_{36}CuF_{10}N$, $(NBu_4)[Cu(C_6F_5)_2]$ **2**, suitable single crystals were obtained by layering hexane in a CH_2Cl_2 solution of the compound at -32 ºC under nitrogen, the crystal was submerged in immersion oil, attached to a loop and transferred to the diffractometer.

Diffraction data for the crystal were recorded in an Oxford Diffraction Super Nova diffractometer with an Atlas CCD area detector. The crystal was kept at 210 K during data collection. Data collection was performed with Mo-Kα radiation (λ = 0.71073 Å). Data integration, scaling and empirical absorption correction was carried out using the CrysAlis Pro program package.[14] The structure was solved using the programs Olex2.[15] The non-hydrogen atoms were refined anisotropically and hydrogen atoms were placed at idealized positions and refined using the riding model. Refinement proceeded smoothly to give the residuals shown in Table S11. CCDC 2072143 contains the supporting crystallographic data for this paper. These data can be obtained free of charge at www.ccdc.cam.ac.uk/conts/retrieving.html [or from the Cambridge Crystallographic Data Centre, 12, Union Road, Cambridge CB2 1EZ, UK; fax: (internat.) +44-1223/336-033; E-mail: deposit@ccdc.cam.ac.uk].

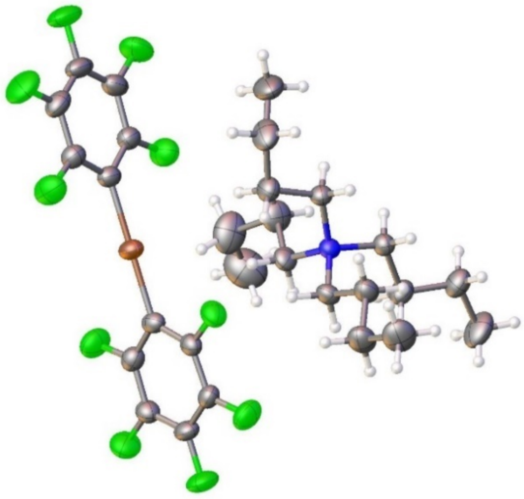

Figure S15: Molecular structure obtained from X-ray diffraction of complex **2** showing 50% probability ellipsoids. Hydrogen atoms have been set in ideal positions. Blue: Nitrogen; Green: Fluorine; Brown: Copper; Grey: Carbon; White: Hydrogen.

Table S11: Crystal data and estructure refinement for $C_{28}H_{36}NF_{10}Cu$

Identification code	CuPf2NBu4 Gabriel A
Empirical formula	$C_{28}H_{36}NF_{10}Cu$
Formula weight	640.12
Temperature/K	210.00(14)
Crystal system	monoclinic
Space group	$P2_1/n$
a/Å	9.3714(2)
b/Å	16.6405(3)
c/Å	18.7821(4)
α/°	90
β/°	90.341(2)
γ/°	90
Volume/Å^3	2928.92(10)
Z	4
ρ_{calc}g/cm^3	1.452
μ/mm^{-1}	0.827
F(000)	1320.0
Crystal size/mm^3	0.937 × 0.619 × 0.414
Radiation	Mo Kα (λ = 0.71073)
2Θ range for data collection/°	6.542 to 60.376
Index ranges	$-12 \le h \le 12$, $-21 \le k \le 22$, $-26 \le l \le 23$
Reflections collected	50296
Independent reflections	7755 [R_{int} = 0.0394, R_{sigma} = 0.0312]
Data/restraints/parameters	7755/0/365
Goodness-of-fit on F^2	1.016
Final R indexes [I>=2σ (I)]	R_1 = 0.0457, wR_2 = 0.1084
Final R indexes [all data]	R_1 = 0.0749, wR_2 = 0.1261
Largest diff. peak/hole / e Å^{-3}	1.01/-0.55

S11. Computational Details

Theoretical calculations were performed at DFT level of theory using Gaussian16 software.[16] The structures of all the intermediates and transition states were optimized in tetrahydrofuran solvent (THF, ε= 7.4257) with the SMD continuum model[17] using the B3LYP functional[18] combined with the Grimme's D3 correction for dispersion.[19] Additional calibration calculations employing a set of functionals were carried out for certain structures (see section S16 in the Supporting Information). Basis set BS1 was used for the optimizations. BS1 includes the 6-31G(d,p) basis set for the main group elements,[20] excluding iodine, and the scalar relativistic Stuttgart-Dresden SDD pseudopotential and its associated double-ζ basis set,[21] complemented with a set polarization functions, for the copper (*f* polarization functions)[22] and iodine (*d* polarization functions)[23] atoms. Frequency calculations were carried out for all the optimized geometries in order to characterize the stationary points as either minima or transition states.

Gibbs energies in tetrahydrofuran were calculated at 298.15 K adding to the potential energies in tetrahydrofuran, obtained with single point calculations using an extended basis set (BS2) at the BS1 optimized geometries, the thermal and entropic corrections obtained with BS1. BS2 consists in the def2-TZVP basis set for the main group elements, and the quadruple-ζ def2-QZVP basis set for Cu.[24] A correction of 1.9 kcal mol^{-1} was applied to all Gibbs values to change the standard state from the gas phase (1 atm) to solution (1 M) at 298.15 K.[25] In this way, all the energy values in the energy profiles are Gibbs energies in THF solution calculated using the formula:

$$G = E(BS2) + G(BS1) - E(BS1) + \Delta G^{1atm \rightarrow 1M}$$

where $\Delta G^{1atm \rightarrow 1M}$ = 1.9 kcal mol^{-1} is the Gibbs energy change for compression of 1 mol of an ideal gas from 1 atm to the 1 M solution phase standard state.

To locate the minimum energy crossing points (MECP) between singlet and triplet potential energy surfaces, the program developed by the group of Harvey was employed.[26] To confirm that the MECP connects the two intermediates located in the two energy surfaces, the MECP structure was optimized in the different spin states involved in the crossing. The Gibbs energies in solution of the MECP was estimated by adding to the calculated potential energy of the MECP thermal and entropic corrections calculated with the option freq = projected of the Gaussian 09 program.[27] 3D-structures were generated using CYLview.[28]

S12. Optimized structures of all the intermediates and transition states in the reaction of [Cu(bipy)Pf] (1) with Rf-I

(Gibbs energy profile in Figure 2 of the main text)

Heterocoupling pathway

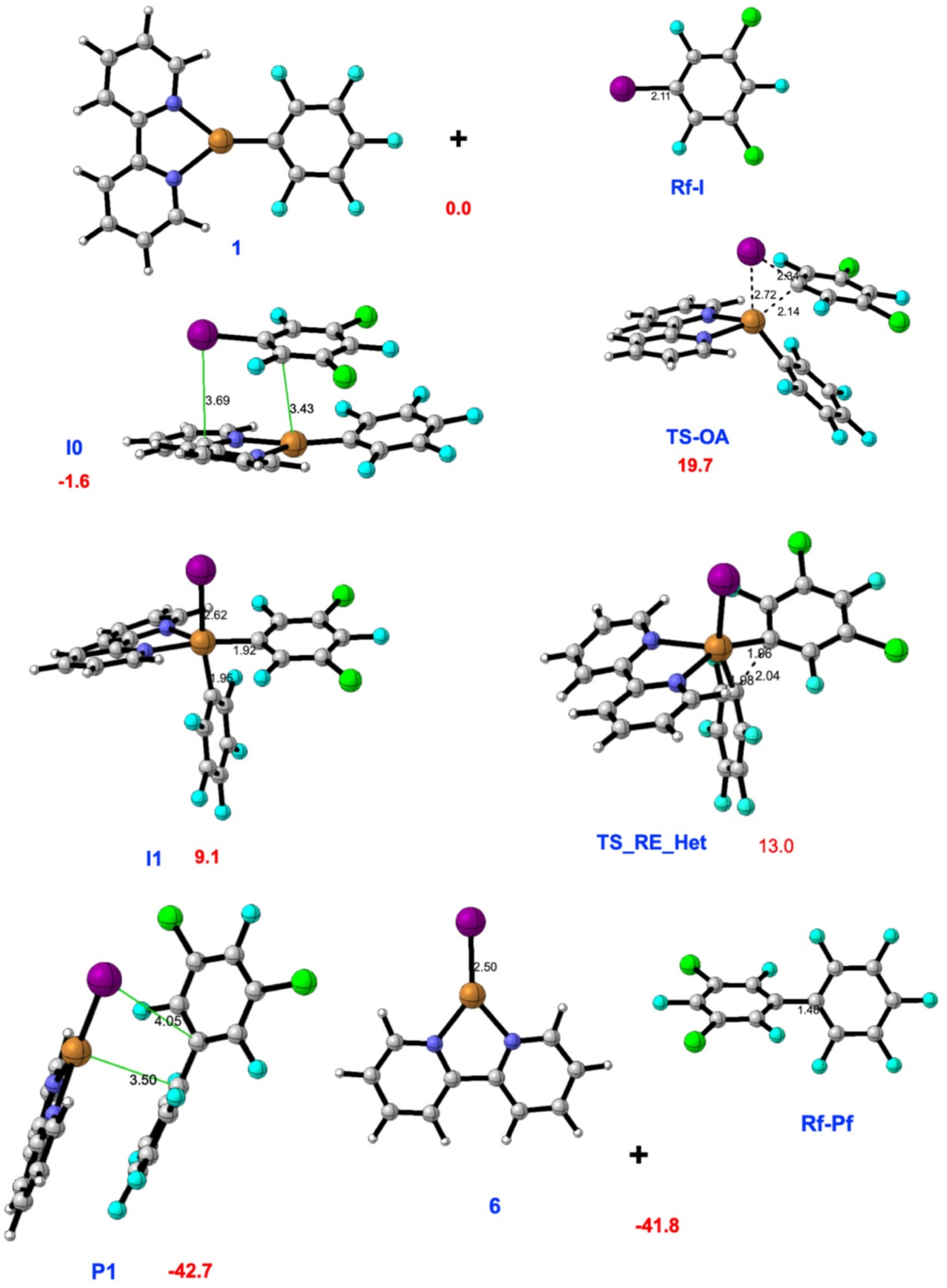

Figure S16. Optimized structures of all the intermediates and transition states in the pathway for the formation of the heterocoupling product, **Rf-Pf**. In red, relative Gibbs energies in THF, in kcal mol^{-1}.

Homocoupling pathway

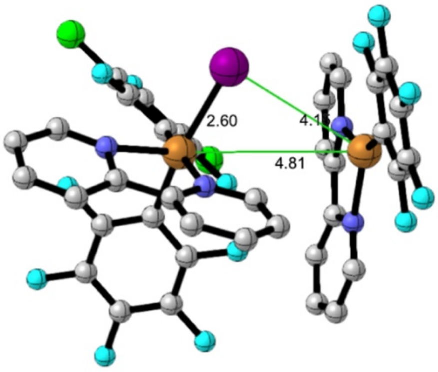

S1 **9.1**

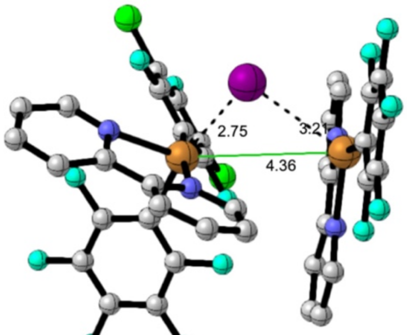

MECP1 **12.9**

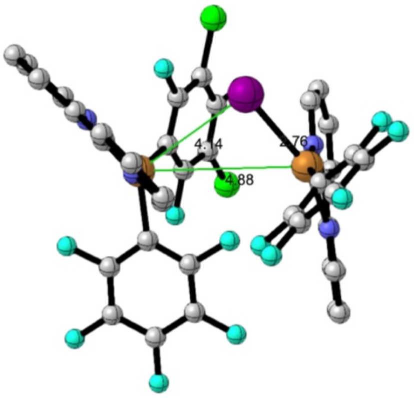

-2.6 **T1**

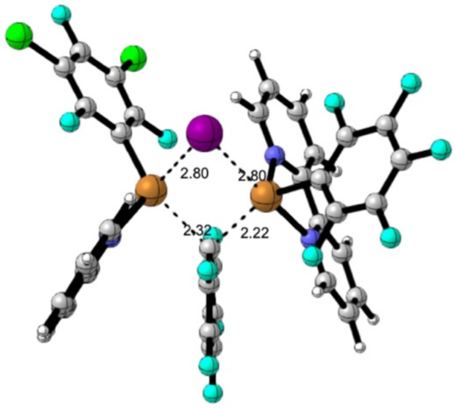

TS_Transmet **11.3**

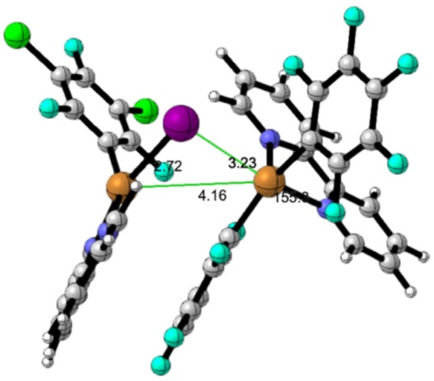

2.2 **T2**

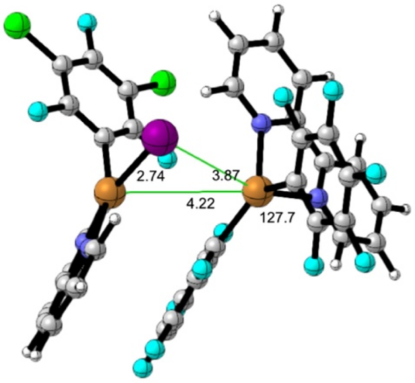

TS_t-c **6.5**

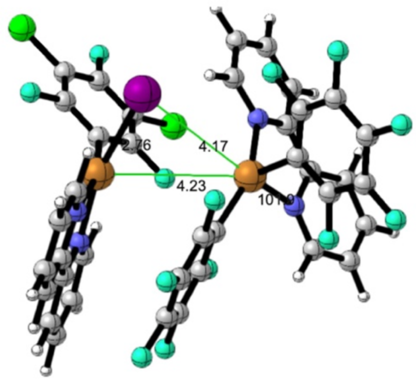

-0.6 T3

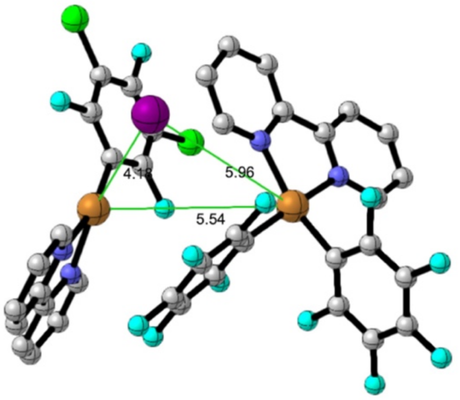

12.1 MECP2

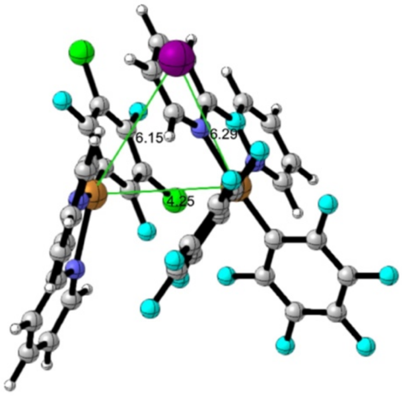

6.7 S2

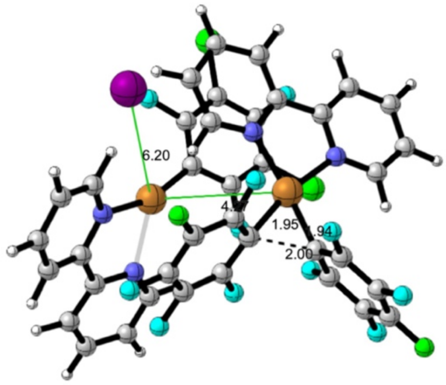

TS_RE_Homo 7.9

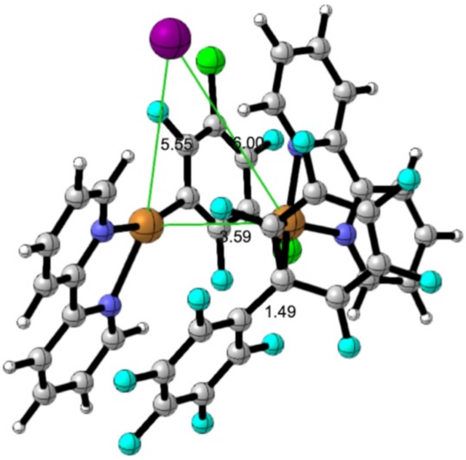

S3 -25.7

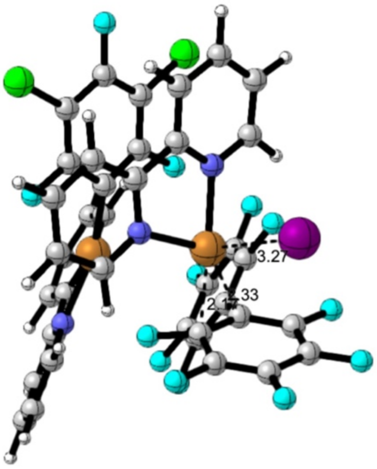

TS_I -26.7

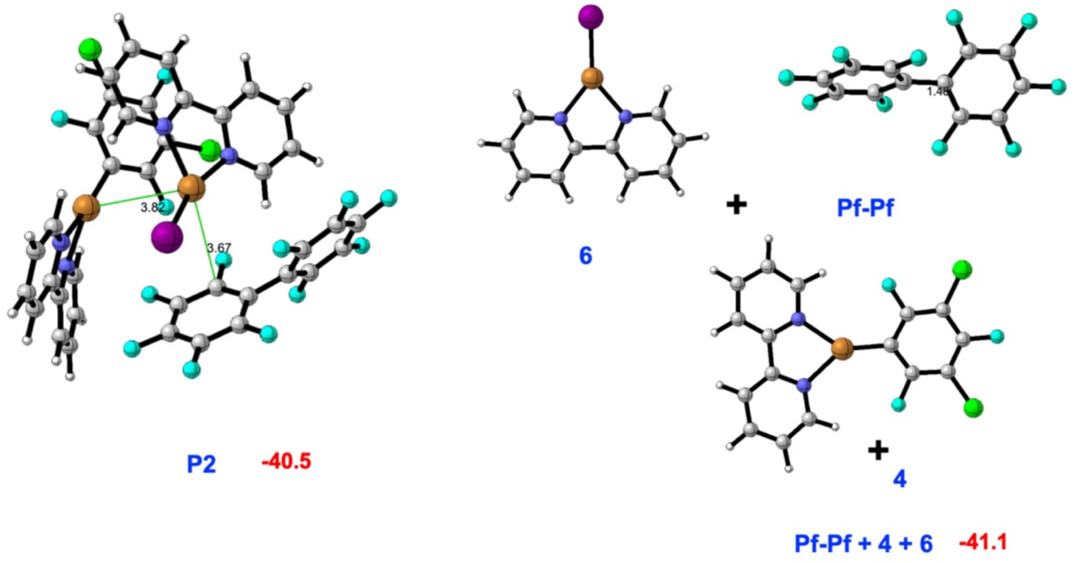

Figure S17. Optimized structures of all the intermediates and transition states in the pathway for the formation of the homocoupling product, **Pf-Pf**. In red, relative Gibbs energies in THF, in kcal mol^{-1}.

S13. Spin-density plots

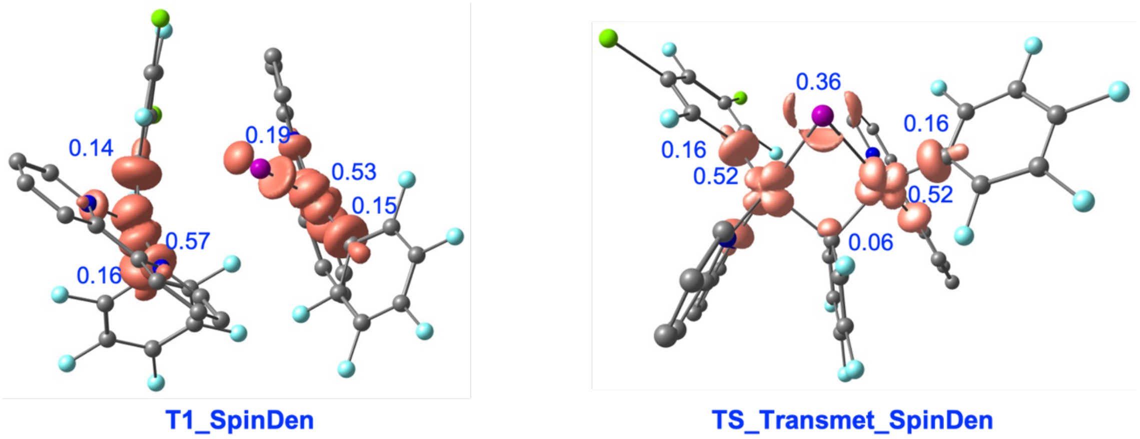

Figure S18. Spin density plots of intermediate **T1** (left) and transmetalation transition state (right). In blue Mulliken atomic spin populations

Table S12: Atomic charges[a] at the copper centers of intermediates **I0**, **S1** and **T1**.

intermediate	Cu oxidation state	Spin state	Atomic charge
**I0**	Cu(I)	singlet	0.340
**S1**	Cu(III)	singlet	0.632
	Cu(I)		0.349
**T1**	Cu(II)	triplet	0.617 (0.566)[b]
	Cu(II)		0.642 (0.532)[b]

[a] Computed with the CM5 model.[29]
[b] Mulliken spin populations.

S14. Gibbs energy profile for the reaction of [Cu(bipy)Pf] (1) with *p*-substituted phenyl iodides (*p*-X-Ar-I; X = H, OMe, Br, NO2)

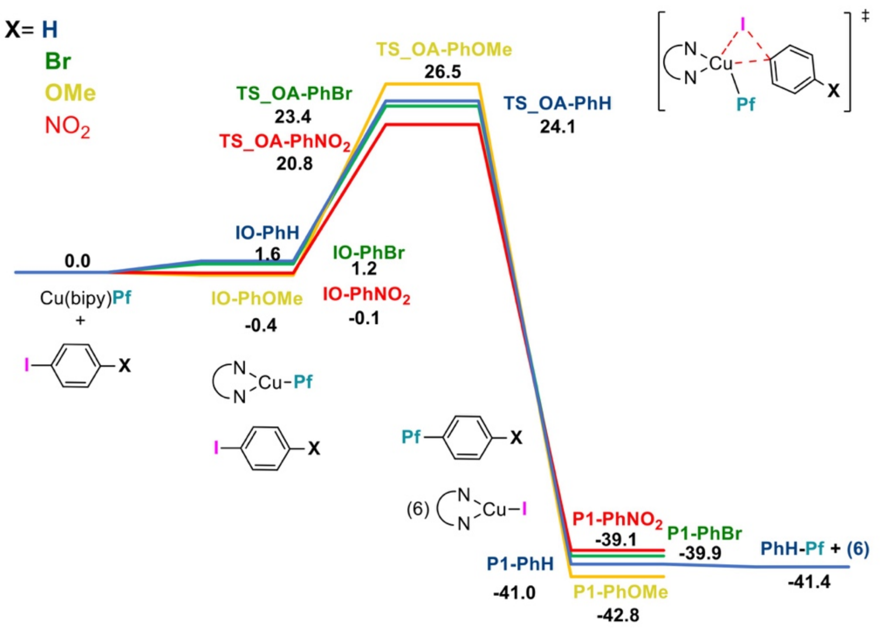

Figure S19. DFT-computed (B3LYP-D3/BS2 in THF) pathway for the formation of the heterocoupling product ***p*-X-Ar-Pf** in the reaction of **1** with *p*-substituted phenyl iodides. Relative Gibbs energies are given in kcal mol^{-1}.

Table S13: Atomic charges[a] at ipso-carbon of para-substituted aryl iodides in the intermediates preceding oxidative addition.

substituent	Atomic charge
**OMe**	-0.038
**H**	-0.022
**Br**	-0.019
**NO_2**	-0.003

[a] Computed with the CM5 model.[29]

S15. Optimized structures of all the intermediates and transition states in the reaction of [Cu(bipy)Pf] (1) with Ph-I (Gibbs energy profile in Figure S19)

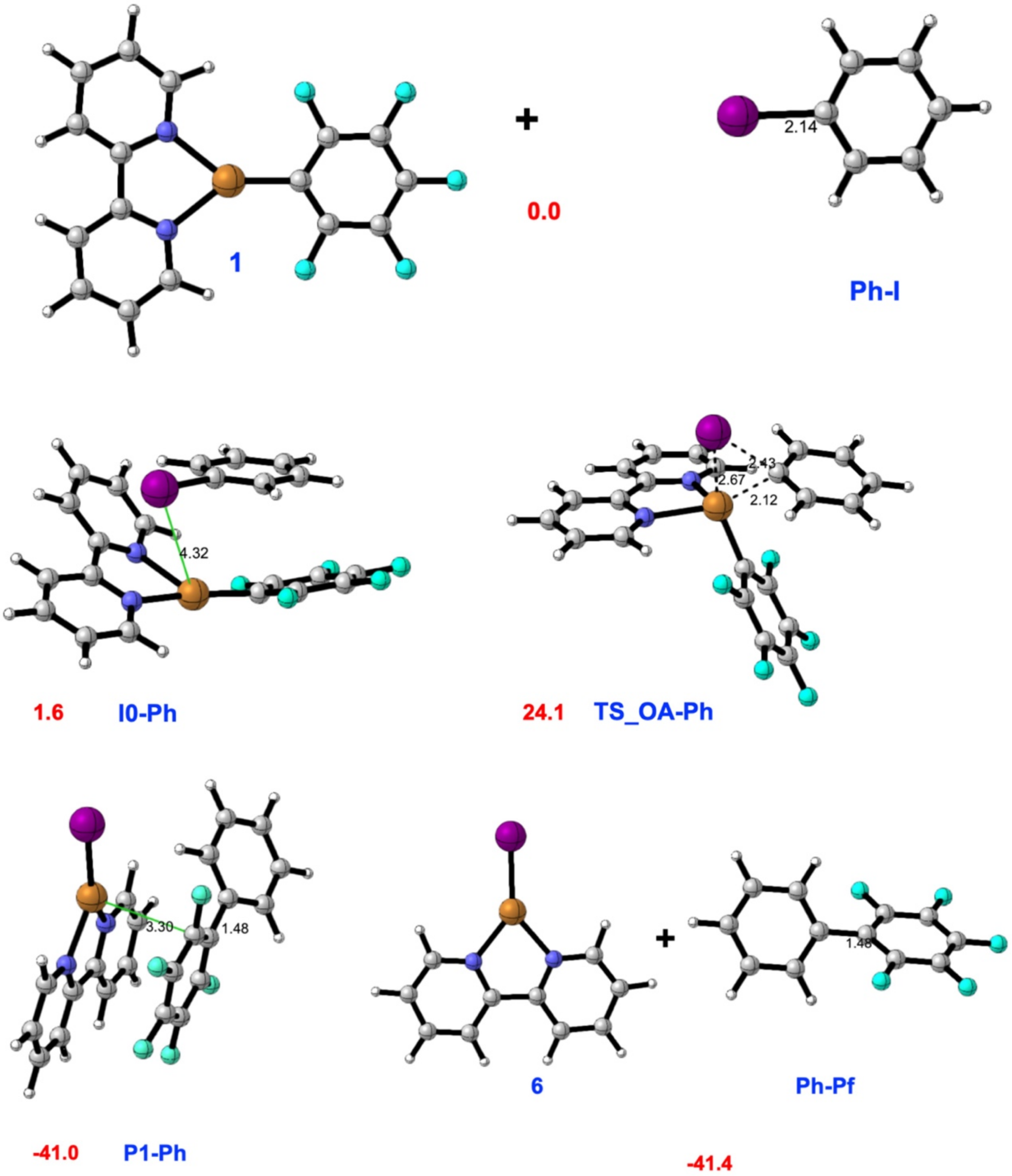

Figure S20. Optimized structures of all the intermediates and transition states in the pathway for the formation of the heterocoupling product, **Ph-Pf**. In red, relative Gibbs energies in THF, in kcal mol^{-1}.

S16. DFT benchmark study of the relative stabilities of Cu^{I}-Cu^{III} (S1) and Cu^{II}-Cu^{II} (T1) species

Table S14. Relative energies in THF of **S1** (Cu(III)-Cu(I), singlet) and **T1** (Cu(II)-Cu(II), triplet) intermediates with respect 2 **1** + **Rf-I** and **T1-S1** energy difference (ΔE(T1-S1), calculated with different functionals . Energies in kcal mol^{-1}. All single-point calculations are done using BS2 on B3LYP-D3/BS1 singlet and triplet optimized geometries in THF.

Method	%HF exchange	S1/singlet	T1/triplet	ΔE(T1-S1)
PBE-D3/BS2	0	-21,3	-23,1	-1,8
M11-L/BS2	0	-14,6	-30,3	-15,7
TPSSh-D3/BS2	10	-22,9	-32,0	-9,1
B3LYP-D3/BS2	20	-19,0	-29,6	-10,5
PBE0-D3/BS2	25	-15,6	-29,2	-13,7
M06/BS2	27	-2,3	-6,1	-3,8
M06-D3/BS2	27	-13,6	-16,9	-3,3
MN15/BS2	44	-6,5	-9,8	-3,3
M06-2X-D3/BS2	54	-3,3	-38,2	-38,2
CAM-B3LYP-D3/BS2	19(SR)65(LR)[a]	-13,0	-27,1	-14,1
wB97XD/BS2	22(SR)/100(LR)[a]	-9,7	-25,2	-15,5
M11/BS2	42.8(SR)/100(LR)[a]	-5,3	-28,1	-22,7

[a] SR=short range, LR=long range

S17. Cartesian coordinates and absolute E and G energies in THF of the optimized structures

Reaction between [Cu(bipy)Pf] and Rf-I (Gibbs energy profile in Figure 2 of the main text)

1 [Cu(bipy)Pf]

Charge	0
Spin State	Singlet
Electronic Energy, BS1 (a.u.)	-1420.627334
Thermal and entropic correction, BS1 (a.u.)	0.155681
Electronic Energy, BS2 (a.u.)	-2864.399240
Gibbs Energy, BS2 (a.u.)	-2864.243559
Number of Imaginary Frequencies	0

Molecular Geometry in Cartesian Coordinates

C	-4.400490	-1.514032	-0.049028	H	-5.177712	3.527585	0.089065
C	-3.234283	-0.742748	-0.007870	H	-2.889377	4.572520	0.033581
C	-1.915118	-2.657293	0.007582	N	-2.013703	-1.321513	0.017279
C	-3.030299	-3.492355	-0.028083	N	-2.009552	1.321652	-0.017323
C	-4.293791	-2.903964	-0.058203	Cu	-0.356540	-0.004710	0.000286
H	-5.377165	-1.046101	-0.077693	C	1.564900	-0.002051	0.000238
H	-0.904505	-3.053126	0.029115	C	2.316418	1.166716	-0.022196
H	-2.904347	-4.569485	-0.033586	C	2.318763	-1.169337	0.022454
H	-5.189208	-3.516976	-0.089410	C	3.708282	1.202356	-0.023211
C	-3.231945	0.746954	0.007676	C	3.710694	-1.202187	0.023049
C	-4.395668	1.522010	0.048720	C	4.410846	0.000783	-0.000195
C	-1.906482	2.657001	-0.007548	F	1.694765	-2.386425	0.045392
C	-4.284327	2.911600	0.057946	F	1.690033	2.382644	-0.044991
H	-5.373941	1.057401	0.077262	F	4.388353	2.367380	-0.045636
C	-3.018895	3.495805	0.028013	F	5.756139	0.002124	-0.000408
H	-0.894534	3.049409	-0.028937	F	4.393110	-2.365851	0.045258

R-I

Charge	0
Spin State	Singlet
Electronic Energy, BS1 (a.u.)	-1459.932205
Thermal and entropic correction, BS1 (a.u.)	0.007392
Electronic Energy, BS2 (a.u.)	-1746.619319
Gibbs Energy, BS2 (a.u.)	-1746.611927
Number of Imaginary Frequencies	0

Molecular Geometry in Cartesian Coordinates

C	-0.190474	1.197664	-0.000009	F	0.461535	-2.362088	0.000003
C	-1.587109	1.214343	-0.000010	F	-3.606665	-0.000012	-0.000004
C	-2.274207	0.000004	-0.000005	F	0.461515	2.362098	0.000001
C	-1.587096	-1.214351	-0.000009	I	2.632768	-0.000000	0.000007
C	-0.190480	-1.197667	-0.000013	Cl	-2.457413	-2.716658	-0.000002
C	0.523998	0.000007	-0.000007	Cl	-2.457410	2.716660	-0.00000

I0

Charge	0
Spin State	Singlet
Electronic Energy, BS1 (a.u.)	-2880.589496
Thermal and entropic correction, BS1 (a.u.)	0.184712
Electronic Energy, BS2 (a.u.)	-4611.039706
Gibbs Energy, BS2 (a.u.)	-4610.854994
Number of Imaginary Frequencies	0

Molecular Geometry in Cartesian Coordinates

Cu	-0.061147	-0.797375	1.418603
F	-2.266934	-2.836598	0.429119
F	-1.912168	1.634504	2.044519
F	-6.042184	-0.020844	0.495309
C	2.861418	-0.907284	1.612398
N	1.270242	-2.371866	0.579609
C	2.574210	-2.053446	0.708970
C	3.567959	-2.735562	-0.000665
H	4.609574	-2.450375	0.086655
C	4.145849	-0.592420	2.066520
H	4.991772	-1.217597	1.806451
C	-1.966214	-0.619441	1.266789
N	1.792103	-0.154101	1.958862
C	-3.967822	0.808792	1.296204
C	-2.785735	-1.608897	0.737683
C	-2.617218	0.577564	1.539585
C	1.846394	-4.107951	-0.970064
H	1.514718	-4.908766	-1.622169
C	3.224468	1.318290	3.198965
H	3.322236	2.206516	3.813237
C	-4.736297	-0.215796	0.751510
C	0.917102	-3.372055	-0.235386
H	-0.147133	-3.570783	-0.299948
C	-4.141315	-1.441652	0.469008
C	1.972915	0.931061	2.727780
H	1.077569	1.498217	2.959520
C	3.194859	-3.776608	-0.849842
H	3.950154	-4.314831	-1.414197
C	4.327973	0.533123	2.866935
H	5.319314	0.791014	3.226365
C	0.098347	2.187172	-0.998760
C	1.058131	1.227975	-1.322176
C	0.624733	0.009882	-1.846550
C	-0.730852	-0.258043	-2.046579
C	-1.664189	0.724616	-1.715762
C	-1.263533	1.957225	-1.200573
F	0.478220	3.360410	-0.485191
F	-2.962910	0.476146	-1.883103
F	1.517372	-0.933959	-2.161276
I	3.108511	1.631174	-1.054324
F	-4.884707	-2.432760	-0.063787
F	-4.540829	2.001574	1.554229
Cl	-1.255229	-1.786174	-2.676239
Cl	-2.437258	3.172322	-0.81139

TS_OA

Charge	0
Spin State	Singlet
Electronic Energy, BS1 (a.u.)	-2880.556950
Thermal and entropic correction, BS1 (a.u.)	0.185326
Electronic Energy, BS2 (a.u.)	-4611.006402
Gibbs Energy, BS2 (a.u.)	-4610.821076
Number of Imaginary Frequencies	1
Imaginary Frequencies (cm^{-1})	-134.1i

Molecular Geometry in Cartesian Coordinates

Cu	-0.910718	0.192393	-0.052244
F	1.062543	0.672760	-2.520909
F	0.088771	-2.894239	0.416132
F	4.560886	-1.624071	-0.356982
C	-3.511431	-0.097017	1.490565
N	-2.695271	1.357703	-0.246855
C	-3.763703	0.967851	0.480013
C	-5.020907	1.550511	0.277514
H	-5.883721	1.222390	0.844199
C	-4.457780	-0.468539	2.453751
H	-5.427963	0.011162	2.496398
C	0.508243	-1.076432	-1.035746
N	-2.292469	-0.675620	1.447486
C	2.330849	-2.289722	0.045450
C	1.470466	-0.314158	-1.710094
C	0.974310	-2.101473	-0.208862
C	-4.043998	2.960400	-1.409393
H	-4.107910	3.742416	-2.158159
C	-2.868321	-2.043470	3.331628
H	-2.570014	-2.810646	4.037848
C	3.256416	-1.462563	-0.591273
C	-2.828509	2.329034	-1.158755
H	-1.924143	2.600236	-1.692163
C	2.831614	-0.469195	-1.479591
C	-1.979001	-1.614807	2.348207
H	-0.980860	-2.027640	2.270890
C	-5.159795	2.554370	-0.678220
H	-6.129415	3.012222	-0.847812
C	-4.131658	-1.456997	3.379770
H	-4.853802	-1.756166	4.133054
C	0.980717	2.470134	0.240269
C	0.558466	1.257706	0.758810
C	1.374656	0.717049	1.738776
C	2.554975	1.309938	2.178449
C	2.940768	2.526266	1.621149
C	2.148785	3.115251	0.638955
F	0.259911	3.081411	-0.746857
F	4.072836	3.127423	2.027183

F	1.057008	-0.489215	2.300883	Cl	2.865689	-3.515288	1.155622
I	-1.527415	-1.447913	-2.129508	F	2.527922	4.290290	0.098433
Cl	3.990760	0.579400	-2.241104	F	3.326257	0.737496	3.123814

I1

Charge	0
Spin State	Singlet
Electronic Energy, BS1 (a.u.)	-2880.585650
Thermal and entropic correction, BS1 (a.u.)	0.189874
Electronic Energy, BS2 (a.u.)	-4611.027897
Gibbs Energy, BS2 (a.u.)	-4610.838023
Number of Imaginary Frequencies	0

Molecular Geometry in Cartesian Coordinates

Cu	-0.483020	-0.542997	-0.322862	H	-1.659401	-0.576323	-2.919920
F	1.645270	0.419884	-2.296218	C	3.609754	0.135504	-1.031517
F	1.321993	-1.264659	2.096987	C	-1.644540	-0.837964	2.959424
F	5.535201	-0.161732	0.303211	H	-0.607386	-1.034135	3.201154
C	-3.207612	-0.502725	1.290374	C	-4.939213	-0.467559	-2.087177
N	-2.393083	-0.498910	-0.994355	H	-5.940862	-0.456903	-2.505371
C	-3.465824	-0.472323	-0.172777	C	-3.911543	-0.394508	3.588869
C	-4.760365	-0.459782	-0.707913	H	-4.684243	-0.246858	4.337389
H	-5.621880	-0.464708	-0.052222	C	-0.589970	2.209991	-1.201786
C	-4.223793	-0.302218	2.234844	C	-0.359111	1.386471	-0.107706
H	-5.236819	-0.072271	1.928396	C	-0.114334	2.002858	1.112549
C	1.418073	-0.398907	-0.098202	C	-0.145441	3.387885	1.259301
N	-1.937297	-0.751102	1.658303	C	-0.404338	4.189395	0.151691
C	3.434737	-0.700988	1.238062	C	-0.625163	3.598080	-1.088090
C	2.220068	0.052538	-1.136122	F	-0.801213	1.692778	-2.430276
C	2.048165	-0.771845	1.079186	F	-0.436162	5.522235	0.277655
C	-3.820281	-0.507072	-2.917752	F	0.194515	1.279280	2.207067
H	-3.909139	-0.529438	-3.997864	I	-0.231782	-3.086207	-0.898774
C	-2.599314	-0.677598	3.963465	Cl	4.192444	-1.189067	2.725970
H	-2.312656	-0.764665	5.006040	Cl	4.580507	0.702650	-2.358765
C	4.207815	-0.239585	0.172288	F	0.084452	3.954436	2.453729
C	-2.563704	-0.529058	-2.327135	F	-0.871553	4.366312	-2.160260

TS_RE_Het

Charge	0
Spin State	Singlet
Electronic Energy, BS1 (a.u.)	-2880.580332
Thermal and entropic correction, BS1 (a.u.)	0.188553
Electronic Energy, BS2 (a.u.)	-4611.020303
Gibbs Energy, BS2 (a.u.)	-4610.831750
Number of Imaginary Frequencies	1
Imaginary Frequencies (cm^{-1})	

Molecular Geometry in Cartesian Coordinates

Cu	-0.375963	-0.672818	-0.186477	C	-4.630036	-1.123121	-0.844102
F	1.665113	0.681713	-2.364217	H	-5.520623	-1.116487	-0.228396
F	1.377495	-0.587621	2.181128	C	-4.236369	-0.422263	2.054903
F	5.548318	0.386297	0.261762	H	-5.254078	-0.353017	1.690641
C	-3.170898	-0.665678	1.178715	C	1.411153	0.131709	-0.079328
N	-2.255351	-0.938734	-1.038273	N	-1.896301	-0.717417	1.614990
C	-3.368854	-0.902355	-0.276458	C	3.456805	-0.056782	1.261574

C	2.224288	0.452306	-1.169849	H	-4.783458	-0.069646	4.107321
C	2.072001	-0.137706	1.126058	C	-0.739669	1.884488	-1.341736
C	-3.570906	-1.427473	-2.980137	C	-0.265180	1.299995	-0.159298
H	-3.601182	-1.637034	-4.043460	C	-0.312288	2.099860	0.987096
C	-2.654905	-0.345874	3.861350	C	-0.812815	3.397460	0.972393
H	-2.403624	-0.236364	4.910782	C	-1.278410	3.946789	-0.216932
C	4.222825	0.296943	0.150547	C	-1.243902	3.180896	-1.376960
C	-2.351977	-1.206234	-2.349285	F	-0.806202	1.194264	-2.493574
H	-1.416307	-1.243975	-2.893935	F	-1.761324	5.192437	-0.242896
C	3.613338	0.540972	-1.079392	F	0.145137	1.648399	2.165441
C	-1.649713	-0.569968	2.921523	I	0.360194	-3.212476	-0.429131
H	-0.610563	-0.627091	3.218318	Cl	4.572610	0.954731	-2.468226
C	-4.731049	-1.380086	-2.208190	Cl	4.229612	-0.431520	2.772692
H	-5.703406	-1.554280	-2.658209	F	-0.838336	4.121935	2.099188
C	-3.971093	-0.259368	3.412682	F	-1.724023	3.685049	-2.521859

P1

Charge	0
Spin State	Singlet
Electronic Energy, BS1 (a.u.)	-2880.669175
Thermal and entropic correction, BS1 (a.u.)	0.189147
Electronic Energy, BS2 (a.u.)	-4611.109654
Gibbs Energy, BS2 (a.u.)	-4610.920507
Number of Imaginary Frequencies	0

Molecular Geometry in Cartesian Coordinates

Cu	-0.770416	1.439306	-0.784188	H	-1.884128	3.298082	1.506169
F	2.127358	-1.211163	2.881470	C	3.888093	-0.747715	1.394041
F	1.264642	-1.194418	-1.745567	C	-1.670571	-0.815603	-2.624526
F	5.603081	-0.310972	-0.161331	H	-0.673319	-0.690603	-3.028374
C	-3.273905	-0.009555	-1.144768	C	-5.042923	2.060057	1.508049
N	-2.567137	1.790433	0.285071	H	-6.014186	2.160925	1.982176
C	-3.593392	0.997990	-0.093402	C	-3.770596	-1.951595	-2.482280
C	-4.853000	1.112149	0.503508	H	-4.445427	-2.749909	-2.773844
H	-5.678417	0.483868	0.192099	C	-0.537452	-0.686720	1.702344
C	-4.164973	-1.019425	-1.525089	C	0.205397	-1.502670	0.841414
H	-5.141457	-1.100363	-1.064906	C	-0.476270	-2.547836	0.208596
C	1.633653	-1.234782	0.579553	C	-1.833819	-2.767426	0.410738
N	-2.038041	0.074289	-1.690085	C	-2.542733	-1.940973	1.277142
C	3.452748	-0.788570	-0.996044	C	-1.890739	-0.902400	1.932757
C	2.549426	-1.063411	1.621007	F	0.040553	0.364401	2.300257
C	2.120242	-1.085464	-0.721319	F	-3.857145	-2.116503	1.444861
C	-3.977018	2.870950	1.892662	F	0.176025	-3.363999	-0.630915
H	-4.082999	3.616125	2.673492	I	1.456862	2.595069	-0.739864
C	-2.502192	-1.848218	-3.050291	Cl	4.991243	-0.535785	2.716949
H	-2.154531	-2.551300	-3.798982	Cl	4.003965	-0.587589	-2.627375
C	4.328032	-0.615398	0.076146	F	-2.466208	-3.753487	-0.237212
C	-2.752860	2.699993	1.248481	F	-2.582789	-0.078449	2.729150

Rf-Pf

Charge	0
Spin State	Singlet

Electronic Energy, BS1 (a.u.)	-2176.312034
Thermal and entropic correction, BS1 (a.u.)	0.049447
Electronic Energy, BS2 (a.u.)	-2176.943858
Gibbs Energy, BS2 (a.u.)	-2176.894413
Number of Imaginary Frequencies	0

Molecular Geometry in Cartesian Coordinates

F	1.177573	1.919905	-1.389996	C	-1.114301	-1.135770	-0.362848
F	1.177706	-1.919979	1.390082	C	-2.508538	-1.159388	-0.364545
F	5.242984	0.000038	-0.000030	C	-3.194860	0.000033	0.000064
C	1.093067	-0.000085	-0.000036	C	-2.508439	1.159416	0.364605
C	3.211002	-0.984788	0.694842	F	-0.451884	2.236508	0.731877
C	1.820655	0.972202	-0.694002	F	-4.526942	0.000087	0.000074
C	1.820711	-0.972312	0.693970	F	-0.452096	-2.236592	-0.732060
C	3.907765	-0.000005	-0.000029	F	3.880077	1.927783	-1.368868
C	3.210944	0.984754	-0.694877	F	3.880188	-1.927754	1.368868
C	-1.114204	1.135696	0.362837	Cl	-3.376140	-2.591002	-0.821373
C	-0.384389	-0.000071	0.000014	Cl	-3.375914	2.591116	0.821403

6 [Cu(bipy)I]

Charge	0
Spin State	Singlet
Electronic Energy, BS1 (a.u.)	-704.323242
Thermal and entropic correction, BS1 (a.u.)	0.116440
Electronic Energy, BS2 (a.u.)	-2434.144082
Gibbs Energy, BS2 (a.u.)	-2434-027626
Number of Imaginary Frequencies	0

Molecular Geometry in Cartesian Coordinates

Cu	0.579587	-0.000836	0.000123	C	-2.068460	3.498446	0.006078
C	-2.256743	0.745976	-0.000849	H	-1.947984	4.576139	0.011951
N	-1.040194	-1.332338	-0.039584	C	-0.951327	-2.669289	-0.043131
C	-2.257280	-0.745008	0.000834	H	0.054628	-3.075456	-0.077269
C	-3.427017	-1.511311	0.045252	C	-0.949408	2.669281	0.043642
H	-4.400933	-1.039393	0.088122	H	0.056840	3.074705	0.078146
C	-3.425912	1.513129	-0.045678	C	-3.330110	-2.901645	0.040808
H	-4.400171	1.041951	-0.088932	H	-4.229454	-3.508611	0.074633
N	-1.039257	1.332411	0.040013	C	-3.327981	2.903396	-0.041161
C	-2.071006	-3.497619	-0.005939	H	-4.226866	3.511026	-0.075309
H	-1.951322	-4.575400	-0.011729	I	3.078960	-0.000252	-0.000100

S1

Charge	0
Spin State	Singlet
Electronic Energy, BS1 (a.u.)	-4301.246350
Thermal and entropic correction, BS1 (a.u.)	0.369521
Electronic Energy, BS2 (a.u.)	-7475.448116
Gibbs Energy, BS2 (a.u.)	-7475.078595
Number of Imaginary Frequencies	0

Molecular Geometry in Cartesian Coordinates

Cu	-0.913024	0.974039	-0.436376	C	-0.529400	4.093327	-0.154461
F	-1.753749	-1.796892	0.767829	N	0.591137	2.022653	0.428268
F	-2.917158	1.139891	-2.744471	C	0.588941	3.373515	0.504949
F	-5.321528	-2.856802	-2.119386	C	1.631249	4.043180	1.156762

H	1.647166	5.125054	1.189326	C	2.057452	-1.699899	3.359093
C	-0.759352	5.462096	0.041776	C	1.181784	-1.797487	4.438555
H	-0.146588	6.041932	0.721030	C	0.016249	-2.545781	4.277613
C	-2.277382	-0.281214	-0.964888	H	-1.137907	-3.741863	2.910295
N	-1.309434	3.350423	-0.960703	H	2.975621	-1.123299	3.417284
C	-4.131667	-0.844456	-2.454707	H	1.404147	-1.287690	5.369767
C	-2.513151	-1.474555	-0.295999	H	-0.704503	-2.626687	5.085033
C	-3.107987	0.010501	-2.039152	C	0.525548	-3.636228	0.675977
C	2.649082	1.925255	1.649182	C	-0.425715	-4.619609	0.390534
H	3.433525	1.315913	2.077430	C	1.203475	-3.583839	-1.548920
C	-2.593322	5.305695	-1.500683	C	-0.559617	-5.074177	-0.919815
H	-3.414741	5.741228	-2.059446	H	-1.054265	-5.024591	1.173432
C	-4.339387	-2.029601	-1.749810	C	0.262929	-4.544444	-1.913467
C	1.600041	1.320222	0.968731	H	1.872663	-3.135037	-2.274763
H	1.559825	0.245979	0.838916	H	-1.300352	-5.830319	-1.159340
C	-3.533551	-2.357150	-0.659847	H	0.185135	-4.864443	-2.946685
C	-2.310524	3.945152	-1.617651	N	1.824580	-2.297724	2.183576
H	-2.911592	3.303713	-2.249813	N	1.334759	-3.145556	-0.288384
C	2.660599	3.314815	1.744083	Cu	2.866871	-1.906814	0.315237
H	3.469191	3.828393	2.254519	C	4.377216	-0.830384	-0.188393
C	-1.806336	6.072872	-0.644039	C	4.672148	-0.531767	-1.514002
H	-2.002896	7.131776	-0.507141	C	5.168688	-0.159879	0.733132
C	-1.548837	0.595007	2.346677	C	5.650371	0.375261	-1.912180
C	-2.029157	1.118607	1.154145	C	6.158036	0.761369	0.405431
C	-3.279707	1.718680	1.180726	C	6.400884	1.029174	-0.938568
C	-4.010306	1.846335	2.360804	F	4.963727	-0.351647	2.080114
C	-3.494735	1.331583	3.547009	F	3.975134	-1.134443	-2.522850
C	-2.256652	0.695737	3.540679	F	5.883940	0.637567	-3.214411
F	-0.362816	-0.047203	2.391049	F	7.352048	1.911689	-1.293578
F	-4.189904	1.442317	4.686744	F	6.875103	1.400143	1.353200
F	-3.857410	2.170649	0.048616	Cl	-3.842041	-3.811214	0.244828
I	0.588410	0.225837	-2.425962	Cl	-5.132508	-0.449853	-3.821794
C	-0.224921	-3.178672	3.059846	F	-1.759224	0.181465	4.678010
C	0.707944	-3.038560	2.026777	F	-5.213410	2.441770	2.360572

MECP1

Charge	0
Spin State	Singlet -Triplet
Electronic Energy, BS1 (a.u.)	-4301.234563
Thermal and entropic correction, BS1 (a.u.)	0.365662
Electronic Energy, BS2 (a.u.)	-7475.438113
Gibbs Energy, BS2 (a.u.)	-7475.072451
Number of Imaginary Frequencies	0

Molecular Geometry in Cartesian Coordinates

Cu	-0.801774	0.948974	-0.476715	C	1.721170	4.397667	0.211062
F	-2.085214	-1.409709	1.208385	H	1.762667	5.419178	-0.145972
F	-2.190105	0.301995	-3.200580	C	-0.660699	5.344111	-1.361829
F	-5.040642	-3.237291	-1.970069	H	-0.127365	6.123288	-0.830206
C	-0.378526	3.989906	-1.136508	C	-2.077775	-0.479890	-0.968077
N	0.661765	2.255042	0.196365	N	-1.064411	3.009582	-1.753237
C	0.692084	3.544180	-0.207537	C	-3.613592	-1.462736	-2.612407

C	-2.572995	-1.399464	-0.054269	H	-1.439624	-3.173292	3.538725
C	-2.626645	-0.545639	-2.240654	H	2.590405	-0.395811	3.419347
C	2.668531	2.583530	1.466539	H	1.133940	-0.303783	5.448060
H	3.431963	2.157821	2.104760	H	-0.948716	-1.717632	5.482067
C	-2.349492	4.661371	-2.927946	C	0.112153	-3.397759	1.245739
H	-3.132067	4.877880	-3.647203	C	-0.895130	-4.360881	1.147728
C	-4.086918	-2.354031	-1.650190	C	0.804786	-3.852890	-0.935301
C	1.625146	1.788345	1.006833	C	-1.047581	-5.071115	-0.040267
H	1.551228	0.742856	1.276760	H	-1.560281	-4.549617	1.979931
C	-3.573276	-2.329003	-0.354168	C	-0.182915	-4.817859	-1.104599
C	-2.020943	3.339903	-2.626356	H	1.502627	-3.605237	-1.726798
H	-2.543070	2.512338	-3.091631	H	-1.836353	-5.810463	-0.131727
C	2.713665	3.914906	1.057353	H	-0.267888	-5.348814	-2.046182
H	3.520715	4.565642	1.379647	N	1.427566	-1.775260	2.432341
C	-1.659752	5.679749	-2.272494	N	0.946521	-3.163581	0.208305
H	-1.896234	6.722154	-2.462394	Cu	2.428821	-1.827250	0.447263
C	-1.748217	1.301674	2.277236	C	4.155932	-0.966460	0.150993
C	-2.078847	1.520500	0.951541	C	4.750306	-1.085497	-1.101964
C	-3.337588	2.045693	0.710779	C	4.811646	-0.100958	1.012279
C	-4.225405	2.369699	1.736022	C	5.880412	-0.378050	-1.499476
C	-3.853298	2.140718	3.057885	C	5.937595	0.643371	0.674200
C	-2.602297	1.596177	3.335224	C	6.475719	0.503654	-0.600342
F	-0.550897	0.745753	2.598952	F	4.348876	0.089496	2.287120
F	-4.698560	2.439250	4.058056	F	4.216980	-1.928304	-2.033554
F	-3.779445	2.236072	-0.557988	F	6.404264	-0.523861	-2.731200
I	1.149514	-0.222431	-2.026071	F	7.564057	1.203993	-0.958495
C	-0.544482	-2.564199	3.545483	F	6.504772	1.491213	1.554234
C	0.331680	-2.564656	2.454948	Cl	-4.210717	-3.414736	0.854143
C	1.697470	-1.001926	3.492583	Cl	-4.252322	-1.504784	-4.235193
C	0.880460	-0.956442	4.619837	F	-2.243356	1.350814	4.610365
C	-0.268629	-1.743538	4.636358	F	-5.439786	2.885103	1.468407

T1

Charge	0
Spin State	Triplet
Electronic Energy, BS1 (a.u.)	-4301.263912
Thermal and entropic correction, BS1 (a.u.)	0.367782
Electronic Energy, BS2 (a.u.)	-7475.464925
Gibbs Energy, BS2 (a.u.)	-7475.097143
Number of Imaginary Frequencies	0

Molecular Geometry in Cartesian Coordinates

Cu	1.692199	-1.641897	0.436868	H	1.098615	-6.313689	-1.547040
F	3.322530	1.051654	1.466811	C	2.724527	-0.161828	-0.485986
F	2.258094	-1.193740	-2.570841	N	2.359527	-3.316295	-0.653829
F	4.320643	3.028028	-2.675223	C	3.311966	0.888615	-2.642694
C	1.470513	-4.324828	-0.782874	C	3.283371	0.958583	0.117674
N	0.052270	-2.924286	0.536885	C	2.769437	-0.147713	-1.876798
C	0.142512	-4.070783	-0.170991	C	-2.249698	-3.367704	1.011910
C	-0.960061	-4.918460	-0.305049	H	-3.172840	-3.040206	1.475577
H	-0.887593	-5.835050	-0.877327	C	4.014710	-4.598927	-1.808528
C	1.814933	-5.507553	-1.443589	H	5.026357	-4.668418	-2.192720

C	3.834380	1.993490	-1.974739	H	-0.714381	4.860730	3.953681
C	-1.106420	-2.578321	1.108097	C	0.046858	3.587296	-0.489930
H	-1.111998	-1.633564	1.632416	C	1.030862	4.517522	-0.830954
C	3.825767	2.042390	-0.583057	C	0.177553	2.608597	-2.598186
C	3.595624	-3.447178	-1.148170	C	1.585967	4.481959	-2.108637
H	4.251416	-2.593989	-1.008652	H	1.349020	5.268480	-0.118999
C	-2.167314	-4.559742	0.294961	C	1.146150	3.518612	-3.013009
H	-3.034911	-5.204042	0.192941	H	-0.177982	1.818200	-3.248405
C	3.102136	-5.642390	-1.960645	H	2.354961	5.193968	-2.388394
H	3.387544	-6.555084	-2.474327	H	1.553147	3.451694	-4.015050
C	0.673092	0.063436	2.713468	N	-1.750527	2.792524	0.869340
C	1.526072	-0.944313	2.309978	N	-0.358407	2.641147	-1.369932
C	2.354983	-1.455464	3.291956	Cu	-1.907655	1.437591	-0.715725
C	2.358639	-1.000979	4.608289	C	-3.685807	0.659884	-0.324467
C	1.480734	0.018662	4.967729	C	-4.705255	0.832963	-1.248025
C	0.622720	0.558266	4.012905	C	-3.976746	-0.186018	0.732190
F	-0.175335	0.631917	1.804191	C	-5.939592	0.195005	-1.161158
F	1.456088	0.475903	6.232491	C	-5.184765	-0.865752	0.861752
F	3.242759	-2.447172	2.982181	C	-6.175352	-0.672276	-0.096370
I	-1.300971	-0.748794	-2.279721	F	-3.057971	-0.396504	1.718836
C	-0.255826	4.340564	1.915908	F	-4.508456	1.640454	-2.328118
C	-0.657102	3.584191	0.812621	F	-6.899023	0.389694	-2.085185
C	-2.485294	2.750229	1.986347	F	-7.353891	-1.308341	0.008989
C	-2.159829	3.493485	3.116958	F	-5.398981	-1.713141	1.889739
C	-1.016115	4.288418	3.082350	Cl	4.461707	3.443673	0.243476
H	0.640266	4.947758	1.873458	Cl	3.313240	0.842080	-4.388195
H	-3.347007	2.095369	1.963504	F	-0.231380	1.539674	4.368414
H	-2.778316	3.427484	4.004652	F	3.187060	-1.521731	5.534636

TS_Transmet

Charge	0
Spin State	Triplet
Electronic Energy, BS1 (a.u.)	-4301.239642
Thermal and entropic correction, BS1 (a.u.)	0.365627
Electronic Energy, BS2 (a.u.)	-7475.440563
Gibbs Energy, BS2 (a.u.)	-7475.074936
Number of Imaginary Frequencies	1
Imaginary Frequencies (cm^{-1})	-49.0i

Molecular Geometry in Cartesian Coordinates

Cu	-1.465339	0.424358	-0.496765	N	-2.535886	2.009619	0.424582
F	-2.339262	-1.214421	2.051454	C	-4.849317	-2.175548	-1.070082
F	-3.679864	-0.670250	-2.444514	C	-3.148138	-1.524349	1.000241
F	-5.983479	-3.684184	0.357314	C	-3.816961	-1.245922	-1.224762
C	-2.652272	3.171280	-0.256945	C	-1.300398	2.947726	-4.268350
N	-1.686247	1.985797	-2.108326	H	-0.944733	2.817380	-5.284592
C	-2.179737	3.160298	-1.667240	C	-3.545743	3.008392	2.352170
C	-2.240358	4.282135	-2.502696	H	-3.887471	2.896755	3.375142
H	-2.629794	5.227392	-2.145608	C	-5.005136	-2.787845	0.174806
C	-3.203503	4.303185	0.356342	C	-1.263437	1.881468	-3.371522
H	-3.289839	5.237838	-0.183425	H	-0.871664	0.911021	-3.657944
C	-2.937074	-0.894334	-0.213461	C	-4.152837	-2.467929	1.231690

C	-2.984188	1.924579	1.684941	C	1.399704	-2.101584	4.129578
H	-2.864582	0.963018	2.165330	C	0.142837	-2.669417	1.769947
C	-1.792207	4.170829	-3.817606	C	0.522755	-3.183956	4.074297
H	-1.830442	5.031020	-4.478831	H	1.920807	-1.873529	5.051448
C	-3.646238	4.221826	1.673693	C	-0.120833	-3.477974	2.874329
H	-4.070506	5.095149	2.159040	H	-0.328542	-2.849369	0.809186
C	1.004804	2.429854	-0.560773	H	0.351242	-3.790021	4.958493
C	0.445461	1.477708	0.287063	H	-0.812568	-4.308729	2.788112
C	0.241159	1.922164	1.590631	N	2.777746	0.351813	1.715803
C	0.546687	3.195812	2.045029	N	0.975935	-1.627177	1.820714
C	1.104079	4.106056	1.148036	Cu	1.636834	-0.380986	0.081188
C	1.334014	3.725236	-0.171113	C	3.253997	-1.222882	-0.737049
F	1.269116	2.121801	-1.856685	C	3.570388	-2.567486	-0.630842
F	1.414824	5.344409	1.553865	C	4.164818	-0.447776	-1.434350
F	-0.288286	1.076061	2.515124	C	4.718087	-3.129185	-1.186514
I	0.016245	-1.463476	-1.932757	C	5.327814	-0.952394	-2.010420
C	3.099492	0.401137	4.080510	C	5.603866	-2.310856	-1.882095
C	2.523906	-0.165900	2.936159	F	3.938383	0.891645	-1.591671
C	3.605169	1.397057	1.581898	F	2.739988	-3.418858	0.030900
C	4.215908	2.004713	2.673947	F	4.987120	-4.443599	-1.062545
C	3.947471	1.497420	3.946304	F	6.719062	-2.829325	-2.425351
H	2.878612	0.004884	5.064403	F	6.183678	-0.159086	-2.683676
H	3.766800	1.742726	0.566958	Cl	-4.357473	-3.232019	2.788290
H	4.875459	2.852884	2.525883	Cl	-5.927800	-2.578188	-2.382309
H	4.392687	1.949872	4.826933	F	0.325163	3.567795	3.320538
C	1.603483	-1.334272	2.977199	F	1.870656	4.608648	-1.033832

T2

Charge	0
Spin State	Triplet
Electronic Energy, BS1 (a.u.)	-4301.257248
Thermal and entropic correction, BS1 (a.u.)	0.369215
Electronic Energy, BS2 (a.u.)	-7475.458725
Gibbs Energy, BS2 (a.u.)	-7475.089510
Number of Imaginary Frequencies	0

Molecular Geometry in Cartesian Coordinates

Cu	-2.029171	0.389943	-0.990410	C	-3.285615	-2.195731	-1.477708
F	-1.633869	-1.465577	1.626659	C	-1.755955	3.841222	-3.656792
F	-3.579565	-1.615078	-2.669238	H	-1.211217	4.090714	-4.560414
F	-3.845871	-5.370460	0.174630	C	-4.608257	1.645938	2.313501
C	-3.681573	2.638510	-0.084827	H	-4.932226	1.221333	3.256717
N	-2.158541	2.262261	-1.900139	C	-3.433272	-4.117529	-0.056150
C	-3.067378	3.102921	-1.350861	C	-1.521953	2.624333	-3.022599
C	-3.358625	4.335999	-1.938033	H	-0.804078	1.910790	-3.408102
H	-4.091461	5.000044	-1.497926	C	-2.732031	-3.426819	0.932742
C	-4.512312	3.433422	0.709115	C	-3.788581	0.905502	1.465689
H	-4.771787	4.440154	0.407383	H	-3.459794	-0.088994	1.731759
C	-2.576636	-1.464901	-0.537612	C	-2.694774	4.708396	-3.104723
N	-3.341323	1.387147	0.299429	H	-2.909774	5.664544	-3.571243
C	-3.719078	-3.509005	-1.279718	C	-4.979481	2.929206	1.921019
C	-2.315380	-2.122034	0.652083	H	-5.613023	3.541140	2.554817

C	0.142729	2.635129	0.440139	H	2.910304	-3.073401	4.187522
C	0.450987	1.439487	1.075179	C	0.969864	-3.975824	1.563030
C	-0.187599	1.249102	2.293852	H	0.499064	-2.608019	-0.049988
C	-1.085664	2.159280	2.847629	H	1.647243	-5.020662	3.328356
C	-1.353058	3.344855	2.170970	H	0.403688	-4.802856	1.150166
C	-0.734556	3.588369	0.949922	N	3.085909	0.470085	2.113125
F	0.713338	2.931899	-0.763831	N	1.695106	-1.702410	1.353690
F	-2.228056	4.231994	2.676552	Cu	1.923824	0.309116	0.311923
F	0.053615	0.134637	3.031583	C	3.531129	-0.060502	-0.826055
I	0.182581	-0.529384	-2.274488	C	3.934742	-1.274082	-1.362899
C	3.808398	-0.512054	4.164271	C	4.358748	1.011864	-1.121377
C	3.109546	-0.585710	2.951515	C	5.078728	-1.428994	-2.143629
C	3.732863	1.598112	2.430768	C	5.512558	0.922270	-1.894940
C	4.444980	1.741969	3.617632	C	5.875055	-0.318966	-2.409440
C	4.478157	0.661378	4.498825	F	4.044910	2.259789	-0.652225
H	3.829707	-1.356024	4.842368	F	3.200316	-2.401029	-1.157619
H	3.678465	2.394162	1.696603	F	5.427188	-2.630118	-2.646639
H	4.956411	2.672812	3.837155	F	6.984022	-0.444500	-3.159341
H	5.020394	0.728348	5.436887	F	6.277436	2.001484	-2.154369
C	2.368613	-1.802649	2.514791	Cl	-2.428128	-4.178291	2.477460
C	2.367564	-2.993411	3.253595	Cl	-4.603823	-4.380213	-2.505531
C	1.017258	-2.755810	0.891534	F	-1.711717	1.909480	4.015411
C	1.658506	-4.090253	2.768965	F	-1.022901	4.718327	0.271758

TS_t-c

Charge	0
Spin State	Triplet
Electronic Energy, BS1 (a.u.)	-4301.249067
Thermal and entropic correction, BS1 (a.u.)	0.366608
Electronic Energy, BS2 (a.u.)	-7475.449347
Gibbs Energy, BS2 (a.u.)	-7475.082739
Number of Imaginary Frequencies	1
Imaginary Frequencies (cm^{-1})	-153.4i

Molecular Geometry in Cartesian Coordinates

Cu	2.020940	-0.577444	-0.886140	H	1.482221	-4.855044	-3.803872
F	1.175806	1.595301	1.362260	C	4.154240	-1.273430	2.882854
F	3.897374	1.217718	-2.473225	H	4.368139	-0.700778	3.777921
F	3.617436	5.325707	-0.170235	C	3.257302	4.042304	-0.300789
C	3.501717	-2.646787	0.585317	C	1.670899	-3.138269	-2.528292
N	2.198755	-2.582000	-1.428755	H	1.013922	-2.501992	-3.108114
C	3.023784	-3.317847	-0.645623	C	2.379912	3.472306	0.621872
C	3.347170	-4.636463	-0.972585	C	3.437799	-0.689628	1.841064
H	4.012622	-5.214195	-0.343660	H	3.084451	0.329755	1.906198
C	4.238189	-3.292419	1.581876	C	2.799493	-5.208386	-2.118363
H	4.529075	-4.329287	1.472349	H	3.039558	-6.233204	-2.383168
C	2.499851	1.328018	-0.575212	C	4.567020	-2.595884	2.742709
N	3.125116	-1.355921	0.723104	H	5.125000	-3.089019	3.531972
C	3.771464	3.282074	-1.352978	C	-0.299466	-2.476126	0.814760
C	2.025236	2.130911	0.448258	C	-0.549226	-1.235016	1.380917
C	3.380427	1.944274	-1.450410	C	0.021696	-1.041677	2.630002
C	1.939653	-4.450218	-2.908126	C	0.803698	-1.989251	3.285280

C	1.017413	-3.221009	2.673055	H	-0.517998	2.480099	-1.020138
C	0.459743	-3.471661	1.423897	H	-1.822761	6.122674	0.870325
F	-0.798202	-2.778487	-0.418702	H	-0.477753	4.977439	-0.925221
F	1.787597	-4.148733	3.269114	N	-3.208493	0.653610	2.178621
F	-0.166258	0.138076	3.287862	N	-1.758878	2.269663	0.607099
I	0.138739	0.169607	-2.727439	Cu	-1.882540	0.099219	0.568972
C	-3.858588	2.403375	3.667549	C	-3.327954	-0.274828	-0.722439
C	-3.206490	1.959557	2.510544	C	-3.683227	0.512476	-1.807933
C	-3.852517	-0.235674	2.943051	C	-4.026056	-1.466361	-0.585707
C	-4.531424	0.132807	4.101220	C	-4.656873	0.135729	-2.731062
C	-4.525233	1.478471	4.467827	C	-5.007333	-1.887580	-1.478869
H	-3.839448	3.448599	3.950472	C	-5.323621	-1.073960	-2.562902
H	-3.815318	-1.263808	2.599835	F	-3.757204	-2.304693	0.455486
H	-5.042771	-0.616708	4.695199	F	-3.077233	1.705830	-2.030751
H	-5.032925	1.807747	5.368904	F	-4.959823	0.921533	-3.781357
C	-2.472037	2.866640	1.585422	F	-6.268025	-1.453035	-3.439184
C	-2.516136	4.261301	1.699625	F	-5.652921	-3.056980	-1.311224
C	-1.071154	3.021128	-0.261212	Cl	1.776427	4.416400	1.958191
C	-1.800013	5.040094	0.793724	Cl	4.876571	4.002552	-2.495313
H	-3.104696	4.736552	2.474785	F	1.373589	-1.737752	4.481763
C	-1.058355	4.411947	-0.204832	F	0.700027	-4.651677	0.814636

T3

Charge	0
Spin State	Triplet
Electronic Energy, BS1 (a.u.)	-4301.261225
Thermal and entropic correction, BS1 (a.u.)	0.367660
Electronic Energy, BS2 (a.u.)	-7475.461640
Gibbs Energy, BS2 (a.u.)	-7475.093980
Number of Imaginary Frequencies	0

Molecular Geometry in Cartesian Coordinates

Cu	2.299381	-0.522202	-0.608824	C	2.832232	4.226312	0.003870
F	0.908096	1.530745	1.457291	C	2.389104	-3.112319	-2.230103
F	4.193250	1.490303	-1.935314	H	1.992141	-2.466981	-3.003497
F	2.977069	5.551675	0.129125	C	1.916539	3.550999	0.809685
C	3.333234	-2.554109	1.250446	C	3.150782	-0.516100	2.358019
N	2.618905	-2.527950	-1.045345	H	2.877803	0.527205	2.288979
C	3.113158	-3.273411	-0.025892	C	3.114116	-5.241089	-1.414023
C	3.360686	-4.638888	-0.182190	H	3.299108	-6.302208	-1.547229
H	3.732646	-5.231616	0.643379	C	3.926613	-2.421180	3.578804
C	3.796229	-3.174149	2.414976	H	4.273951	-2.891434	4.493044
H	4.038817	-4.228684	2.422959	C	-0.670622	-2.488792	0.116337
C	2.523629	1.422441	-0.265201	C	-0.782942	-1.375088	0.941339
N	3.021952	-1.239161	1.237762	C	-0.248445	-1.537897	2.214975
C	3.607374	3.528903	-0.924696	C	0.342296	-2.714197	2.667312
C	1.794458	2.168029	0.645842	C	0.440167	-3.794864	1.797758
C	3.431191	2.146782	-1.025454	C	-0.068436	-3.681816	0.509198
C	2.626844	-4.464661	-2.460323	F	-1.136733	-2.462067	-1.156258
H	2.419015	-4.887409	-3.436757	F	1.075677	-4.916836	2.179793
C	3.597062	-1.068088	3.554903	F	-0.261645	-0.506243	3.103147
H	3.674080	-0.447015	4.440063	I	0.813271	0.077504	-2.853241

C	-3.976414	2.978346	3.094174	N	-2.671261	1.116911	2.334778
C	-3.071386	2.396547	2.197479	N	-1.825947	2.357521	0.135253
C	-3.142033	0.383333	3.348111	Cu	-1.707120	0.314410	0.459839
C	-4.040312	0.888804	4.286810	C	-3.189192	-0.346522	-0.731378
C	-4.463602	2.210724	4.150483	C	-3.419258	0.055226	-2.037054
H	-4.311444	4.001078	2.967415	C	-4.063159	-1.302986	-0.238465
H	-2.779352	-0.637392	3.401132	C	-4.440453	-0.464647	-2.830181
H	-4.396751	0.259921	5.095530	C	-5.101711	-1.854915	-0.983568
H	-5.169320	2.641129	4.854822	C	-5.288089	-1.429863	-2.295816
C	-2.484539	3.120139	1.039336	F	-3.925795	-1.766522	1.037023
C	-2.560668	4.507225	0.884946	F	-2.633114	1.000658	-2.620004
C	-1.218147	2.939005	-0.908904	F	-4.623143	-0.048141	-4.098832
C	-1.933281	5.109519	-0.203693	F	-6.284587	-1.942949	-3.038421
H	-3.075787	5.115432	1.618072	F	-5.926672	-2.784757	-0.461758
C	-1.240742	4.315427	-1.114609	Cl	0.976236	4.423652	1.989960
H	-0.704437	2.271107	-1.589221	Cl	4.759601	4.383169	-1.917862
H	-1.975499	6.187071	-0.327604	F	0.862518	-2.809724	3.906917
H	-0.724367	4.741725	-1.967394	F	0.073075	-4.708805	-0.350896

MECP2

Charge	0
Spin State	Triplet - Singlet
Electronic Energy, BS1 (a.u.)	-4301.242122
Thermal and entropic correction, BS1 (a.u.)	0.365919
Electronic Energy, BS2 (a.u.)	-7475.439586
Gibbs Energy, BS2 (a.u.)	-7475.073667
Number of Imaginary Frequencies	0

Molecular Geometry in Cartesian Coordinates

Cu	-2.887920	-0.446081	-0.361185	C	-1.240916	3.242098	-1.766766
F	-0.682714	0.993611	-2.125406	C	-2.770509	-0.992709	-3.491230
F	-4.209155	2.067598	0.832621	H	-2.215681	-0.061092	-3.503759
F	-1.805583	5.496595	-1.328036	C	-4.858663	-4.618751	0.869109
C	-3.787619	-2.616350	-2.174333	H	-5.307465	-5.572746	1.125642
N	-3.703144	-2.187986	0.194462	C	-3.777820	-2.878770	-4.565497
C	-4.077197	-3.050074	-0.783016	H	-4.038322	-3.442459	-5.456193
C	-4.660967	-4.278745	-0.467883	C	0.841770	-1.553420	1.260135
H	-4.946769	-4.970936	-1.250259	C	1.235548	-0.979058	0.060796
C	-4.140073	-3.357350	-3.307524	C	0.702366	-1.494308	-1.112357
H	-4.683568	-4.290195	-3.218036	C	-0.200572	-2.552627	-1.102579
C	-2.467366	1.427783	-0.632847	C	-0.592397	-3.100593	0.115763
N	-3.122179	-1.448337	-2.285145	C	-0.088302	-2.589619	1.308474
C	-3.038015	3.793018	-0.241055	F	1.364020	-1.112517	2.424426
C	-1.489381	1.894140	-1.499388	F	-1.464950	-4.118746	0.141425
C	-3.228111	2.424374	-0.031661	F	1.050549	-0.982093	-2.313025
C	-4.469801	-3.725463	1.864098	I	-2.096768	0.278313	3.675962
H	-4.599231	-3.949229	2.916701	C	4.162280	4.258912	-0.742431
C	-3.075077	-1.678570	-4.665418	C	3.409982	3.152921	-0.342418
H	-2.765808	-1.278736	-5.625113	C	5.121938	1.702847	-0.997326
C	-2.030435	4.192186	-1.120524	C	5.927376	2.761741	-1.404518
C	-3.895537	-2.516755	1.481760	C	5.433138	4.058793	-1.278115
H	-3.569057	-1.783201	2.211887	H	3.763751	5.261425	-0.646657

H	5.450423	0.673263	-1.072572
H	6.912445	2.565381	-1.811924
H	6.029427	4.909885	-1.591169
C	2.072771	3.233723	0.281972
C	1.476762	4.422547	0.698585
C	0.287620	1.989108	1.128834
C	0.253042	4.370848	1.368380
H	1.964811	5.373974	0.528522
C	-0.342250	3.136870	1.606349
H	-0.158235	1.012803	1.264629
H	-0.221893	5.286766	1.704283
H	-1.281460	3.040742	2.136122
N	3.898944	1.896255	-0.483329
N	1.459093	2.039018	0.482051
Cu	2.568309	0.432286	-0.037103
C	3.848821	-1.039620	0.018220
C	4.520376	-1.175895	1.224813
C	4.183802	-1.918267	-0.999611
C	5.498952	-2.144453	1.428848
C	5.161073	-2.898449	-0.835663
C	5.813514	-3.015786	0.388723
F	3.580471	-1.840649	-2.205905
F	4.260241	-0.323583	2.245348
F	6.146744	-2.243632	2.600367
F	6.752095	-3.956590	0.561415
F	5.481234	-3.729194	-1.841187
Cl	0.030690	3.749787	-2.851164
Cl	-3.997943	4.996028	0.582461
F	-0.703269	-3.048819	-2.246382
F	-0.496614	-3.114797	2.472979

S2

Charge	0
Spin State	Singlet
Electronic Energy, BS1 (a.u.)	-4301.262347
Thermal and entropic correction, BS1 (a.u.)	0.372952
Electronic Energy, BS2 (a.u.)	-7475.455239
Gibbs Energy, BS2 (a.u.)	-7475.082287
Number of Imaginary Frequencies	0

Molecular Geometry in Cartesian Coordinates

Cu	-1.026695	-1.229805	1.962324
F	1.918907	-0.294699	2.129190
F	-0.514476	-4.237457	1.187208
F	4.183176	-4.208100	0.801448
C	-2.513030	0.571974	3.825628
N	-2.956928	-0.639199	1.785129
C	-3.434036	0.234827	2.707190
C	-4.692246	0.824673	2.562840
H	-5.042474	1.552016	3.285049
C	-2.927688	1.200847	5.005150
H	-3.973118	1.427344	5.179171
C	0.606769	-2.215085	1.688972
N	-1.222696	0.249510	3.616106
C	1.825404	-4.257992	1.027928
C	1.854643	-1.611787	1.783257
C	0.652960	-3.554309	1.322258
C	-4.986869	-0.403409	0.519115
H	-5.546042	-0.676594	-0.368168
C	-0.631274	1.206629	5.730091
H	0.142980	1.448712	6.450186
C	3.040561	-3.580478	1.119851
C	-3.724205	-0.952246	0.727482
H	-3.305392	-1.659571	0.021579
C	3.071237	-2.241430	1.508828
C	-0.305803	0.561726	4.537872
H	0.716829	0.288224	4.294520
C	-5.475392	0.504474	1.455291
H	-6.446412	0.970231	1.321197
C	-1.970146	1.520417	5.966374
H	-2.268652	2.008306	6.889192
C	-1.342708	1.580478	-1.615179
C	-0.375401	1.527699	-0.614896
C	-0.706679	2.021105	0.642911
C	-1.977120	2.521811	0.909677
C	-2.939818	2.535805	-0.094826
C	-2.625283	2.064449	-1.366900
F	-1.069287	1.113430	-2.846484
F	-4.166244	2.999314	0.163222
F	0.197331	2.049442	1.635152
I	-4.215573	-1.454823	-3.286295
C	4.138184	-2.195441	-2.014456
C	2.977141	-1.462451	-1.767449
C	4.220937	0.503274	-1.535148
C	5.410762	-0.165296	-1.799014
C	5.366674	-1.538747	-2.028431
H	4.087928	-3.263725	-2.183705
H	4.201845	1.568188	-1.343582
H	6.344547	0.384578	-1.808775
H	6.277406	-2.097415	-2.217461
C	1.620108	-2.038291	-1.758711
C	1.331965	-3.359740	-2.090926
C	-0.636164	-1.607209	-1.345161

C	0.009851	-3.802611	-2.042598
H	2.123458	-4.037756	-2.382344
C	-0.990253	-2.915773	-1.661562
H	-1.379890	-0.885892	-1.040885
H	-0.227623	-4.830617	-2.293578
H	-2.031017	-3.209451	-1.614177
N	3.038946	-0.130548	-1.509335
N	0.631647	-1.179614	-1.396820
Cu	1.271650	0.669043	-1.061897
C	1.804463	2.493302	-0.918317
C	1.617333	3.379728	-1.971310
C	2.554812	2.915855	0.173658
C	2.163093	4.660178	-1.951376
C	3.110030	4.193041	0.216850
C	2.905273	5.069143	-0.845927
F	2.821628	2.080346	1.190322
F	0.900184	3.013469	-3.049894
F	1.978833	5.498056	-2.979336
F	3.436331	6.295166	-0.813361
F	3.850630	4.577432	1.264408
Cl	4.597935	-1.399494	1.616566
Cl	1.796274	-5.923709	0.501970
F	-2.282358	2.987763	2.129831
F	-3.550159	2.085806	-2.329691

TS_RE_Homo

Charge	0
Spin State	Singlet
Electronic Energy, BS1 (a.u.)	-4301.258026
Thermal and entropic correction, BS1 (a.u.)	0.36860
Electronic Energy, BS2 (a.u.)	-7475.448999
Gibbs Energy, BS2 (a.u.)	-7475.080391
Number of Imaginary Frequencies	1
Imaginary Frequencies (cm-1)	-220.3i

Molecular Geometry in Cartesian Coordinates

Cu	1.319903	0.876751	1.938232
F	-1.667863	0.225450	2.321609
F	1.052931	3.959637	1.321045
F	-3.642547	4.371911	1.259079
C	2.755582	-1.077699	3.684772
N	3.164746	0.128756	1.634691
C	3.642815	-0.772008	2.530498
C	4.873718	-1.403095	2.333305
H	5.228928	-2.144527	3.039020
C	3.195617	-1.718500	4.848471
H	4.238147	-1.983585	4.981075
C	-0.219052	2.029005	1.827180
N	1.470078	-0.706832	3.527368
C	-1.280971	4.199681	1.328099
C	-1.507818	1.539039	1.994701
C	-0.162076	3.380341	1.510284
C	5.129933	-0.172433	0.284965
H	5.663667	0.087957	-0.621724
C	0.934281	-1.629491	5.671928
H	0.183509	-1.835255	6.427321
C	-2.545386	3.630338	1.477746
C	3.897754	0.420796	0.546831
H	3.476126	1.148738	-0.136678
C	-2.676125	2.284725	1.820974
C	0.583117	-0.976959	4.490722
H	-0.436144	-0.657987	4.293047
C	5.624310	-1.100175	1.198724
H	6.574852	-1.595203	1.026194
C	2.268231	-1.996548	5.851685
H	2.587249	-2.491569	6.763911
C	0.884295	-1.725914	-1.645751
C	-0.093315	-1.732105	-0.636074
C	0.289622	-2.262975	0.603848
C	1.557872	-2.792306	0.812871
C	2.498575	-2.778970	-0.210811
C	2.163905	-2.231295	-1.446085
F	0.634616	-1.139565	-2.830068
F	3.719956	-3.273505	-0.004058
F	-0.571176	-2.325696	1.626637
I	4.398984	1.228489	-3.431809
C	-3.930076	2.897879	-1.693995
C	-2.876223	1.991165	-1.569465
C	-4.377343	0.211011	-1.380499
C	-5.473135	1.056102	-1.522451
C	-5.240315	2.422598	-1.667147
H	-3.735976	3.957576	-1.799631
H	-4.494800	-0.859803	-1.254819
H	-6.477514	0.648834	-1.504661
H	-6.069325	3.116878	-1.758157
C	-1.444725	2.372367	-1.610497
C	-0.991062	3.654419	-1.920055
C	0.745718	1.617575	-1.339028
C	0.381179	3.904439	-1.933226
H	-1.690471	4.449401	-2.145205
C	1.267282	2.872754	-1.638370
H	1.391429	0.784555	-1.093069
H	0.749030	4.897853	-2.167301
H	2.340223	3.018502	-1.646413

N	-3.119130	0.669572	-1.393369	F	-3.005240	-1.742950	1.129895
N	-0.570823	1.376569	-1.326900	F	-1.231727	-2.886971	-3.144751
Cu	-1.454488	-0.400857	-1.039003	F	-2.948908	-4.932400	-3.283638
C	-1.992142	-2.259727	-0.975508	F	-4.656099	-5.451997	-1.214221
C	-2.049029	-3.091061	-2.101632	F	-4.655452	-3.831235	0.982219
C	-2.896504	-2.545147	0.060964	Cl	-4.263986	1.580548	2.005037
C	-2.933452	-4.160371	-2.190951	Cl	-1.132813	5.882578	0.883708
C	-3.787272	-3.611180	-0.012516	F	1.880242	-3.317610	2.001856
C	-3.801926	-4.429782	-1.137536	F	3.076181	-2.177490	-2.418684

S3

Charge	0
Spin State	Singlet
Electronic Energy, BS1 (a.u.)	-4301.314552
Thermal and entropic correction, BS1 (a.u.)	0.368868
Electronic Energy, BS2 (a.u.)	-7475.502919
Gibbs Energy, BS2 (a.u.)	-7475.134051
Number of Imaginary Frequencies	0

Molecular Geometry in Cartesian Coordinates

Cu	-1.099501	0.670748	-1.686114	C	-2.280147	-2.271754	0.833215
F	-0.165458	-2.310238	-1.774765	C	-3.508154	-2.624592	0.289542
F	1.532708	2.015682	-2.658082	C	-4.605704	-1.790809	0.478139
F	4.256432	-1.769342	-3.317943	C	-4.457013	-0.598051	1.178628
C	-4.088449	0.406853	-1.921068	F	-3.125293	0.875112	2.416259
N	-2.485489	1.992157	-1.059476	F	-5.789768	-2.114274	-0.044282
C	-3.796777	1.675377	-1.196349	F	-1.241544	-3.095294	0.622311
C	-4.794730	2.491326	-0.655696	I	1.328085	5.183795	0.446852
H	-5.838593	2.214415	-0.732720	C	4.419585	-3.127765	0.649370
C	-5.371643	0.035458	-2.340444	C	3.426481	-2.143708	0.616109
H	-6.223979	0.683811	-2.178259	C	1.802345	-3.716148	1.179795
C	0.588660	-0.101423	-2.184426	C	2.738321	-4.745207	1.235379
N	-3.022720	-0.389240	-2.139847	C	4.069532	-4.440638	0.959110
C	2.890734	0.144769	-3.042273	H	5.454656	-2.878570	0.450956
C	0.830593	-1.469338	-2.182479	H	0.754980	-3.891437	1.391428
C	1.660977	0.665220	-2.632140	H	2.424391	-5.752213	1.487022
C	-3.085834	3.992925	0.122212	H	4.831961	-5.212754	0.989440
H	-2.755393	4.894487	0.625885	C	3.712423	-0.709060	0.330027
C	-4.435629	-2.012428	-3.193905	C	4.970891	-0.240579	-0.058610
H	-4.529758	-2.978112	-3.678529	C	2.844439	1.455634	0.260388
C	3.074911	-1.237544	-2.973238	C	5.150761	1.124839	-0.274463
C	-2.143702	3.125582	-0.425165	H	5.797791	-0.924633	-0.202532
H	-1.082445	3.334012	-0.350828	C	4.074196	1.993528	-0.106266
C	2.040367	-2.067918	-2.544190	H	1.971348	2.086863	0.383800
C	-3.188949	-1.561557	-2.761258	H	6.121634	1.500838	-0.580224
H	-2.290092	-2.156398	-2.891854	H	4.162507	3.061563	-0.268124
C	-4.433910	3.661816	0.010476	N	2.134168	-2.455030	0.871904
H	-5.200161	4.301730	0.437135	N	2.672124	0.143085	0.472302
C	-5.542009	-1.191414	-2.980618	Cu	0.909511	-0.784457	0.913267
H	-6.530926	-1.501607	-3.303671	C	-0.828239	-0.804182	2.271444
C	-3.219438	-0.262832	1.716631	C	-0.214046	0.479541	2.260911
C	-2.102089	-1.092094	1.563772	C	-0.373474	-1.712865	3.268909

C	0.764431	0.833204	3.217605	F	2.164670	0.199387	5.002163
C	0.613034	-1.380329	4.173155	F	1.053078	-2.275604	5.063317
C	1.186564	-0.097330	4.146669	Cl	2.283097	-3.794950	-2.460678
F	-0.924610	-2.931221	3.334990	Cl	4.176665	1.163313	-3.638534
F	-0.727817	1.474526	1.515122	F	-3.633513	-3.742845	-0.433495
F	1.307941	2.048944	3.178216	F	-5.505166	0.220324	1.328356

TS_I

Charge	0
Spin State	Singlet
Electronic Energy, BS1 (a.u.)	-4301.316900
Thermal and entropic correction, BS1 (a.u.)	0.370831
Electronic Energy, BS2 (a.u.)	-7475.506390
Gibbs Energy, BS2 (a.u.)	-7475.135559
Number of Imaginary Frequencies	1
Imaginary Frequencies (cm^{-1})	-35.2i

Molecular Geometry in Cartesian Coordinates

Cu	-2.208766	0.478826	0.913546	C	-3.153852	-2.350636	-1.233420
F	-1.492400	1.649181	-1.904861	F	-1.867364	-3.432290	0.395896
F	-0.587446	2.466935	2.669944	F	-4.435583	-1.238640	-2.873499
F	0.589452	5.662111	-0.588145	F	0.236469	-0.698907	-2.855759
C	-5.148912	-0.224636	0.568583	C	3.194929	3.413749	-0.281326
N	-3.281259	-0.839392	1.983735	C	2.641354	2.192705	0.118740
C	-4.585725	-1.048566	1.675231	C	2.253100	1.605029	-2.097914
C	-5.341797	-2.003802	2.362497	C	2.788300	2.800476	-2.567485
H	-6.374478	-2.184510	2.091218	C	3.265678	3.721408	-1.637397
C	-6.519024	-0.155104	0.285411	H	3.566663	4.121189	0.449062
H	-7.243568	-0.697805	0.880907	H	1.871928	0.855426	-2.778708
C	-1.091801	1.959205	0.411463	H	2.821004	2.997536	-3.632901
N	-4.247548	0.466129	-0.155693	H	3.686700	4.668592	-1.958676
C	-0.025779	4.087760	1.069225	C	2.527617	1.793068	1.548773
C	-1.006729	2.426322	-0.893522	C	2.877311	2.642026	2.604300
C	-0.572988	2.834853	1.359577	C	1.875117	0.140459	3.051604
C	-3.420075	-2.500780	3.714528	C	2.706515	2.208014	3.916443
H	-2.922264	-3.042466	4.511106	H	3.262869	3.634688	2.414080
C	-6.006954	1.353478	-1.519409	C	2.192404	0.934506	4.150394
H	-6.300759	1.981122	-2.353908	H	1.479620	-0.859449	3.177509
C	0.037285	4.483699	-0.267228	H	2.968066	2.862195	4.742422
C	-2.724312	-1.542101	2.985861	H	2.039215	0.557409	5.155480
H	-1.687118	-1.316800	3.198448	N	2.175338	1.308929	-0.793050
C	-0.459541	3.654365	-1.271915	N	2.042417	0.553992	1.788450
C	-4.662852	1.233671	-1.168583	Cu	1.732754	-0.532068	0.037992
H	-3.881224	1.748951	-1.719291	C	0.587591	-2.443593	-0.638504
C	-4.753995	-2.738722	3.388337	C	0.866758	-2.394069	0.752276
H	-5.331638	-3.486145	3.923062	C	1.479038	-3.204376	-1.435319
C	-6.947581	0.643633	-0.773702	C	1.919722	-3.148941	1.311018
H	-8.005509	0.710871	-1.009077	C	2.532634	-3.907594	-0.892461
C	-1.914198	-2.617312	-0.668890	C	2.753040	-3.881294	0.493868
C	-0.736035	-2.069906	-1.191455	F	1.273140	-3.267223	-2.756533
C	-0.858707	-1.253891	-2.316707	F	-0.070266	-1.930308	1.616379
C	-2.090137	-0.968428	-2.892192	F	2.125569	-3.107989	2.633004
C	-3.241598	-1.525882	-2.349732	F	3.793246	-4.537717	1.003966

F	3.368485	-4.595987	-1.672671	F	-2.175508	-0.139394	-3.936891
Cl	-0.411829	4.172076	-2.938790	F	-4.267929	-2.863696	-0.695132
Cl	0.565107	5.159512	2.317198	I	4.879300	-1.144506	-0.589235

P2

Charge	0
Spin State	Singlet
Electronic Energy, BS1 (a.u.)	-4301.326982
Thermal and entropic correction, BS1 (a.u.)	0.368582
Electronic Energy, BS2 (a.u.)	-7475.526096
Gibbs Energy, BS2 (a.u.)	-7475.157514
Number of Imaginary Frequencies	0

Molecular Geometry in Cartesian Coordinates

Cu	-2.830598	0.946750	0.312862	F	1.755984	0.237474	1.935395
F	-0.789272	-0.711410	1.957109	I	0.534163	1.702182	-2.351795
F	-4.836534	-1.330435	-0.406165	C	0.522577	-4.730586	-0.633067
F	-2.774202	-4.984735	1.746592	C	0.111542	-3.422513	-0.909021
C	-2.564024	3.724049	1.512366	C	1.932194	-2.575363	0.260486
N	-2.843870	2.691238	-0.653221	C	2.411565	-3.847178	0.559264
C	-2.679973	3.847725	0.034021	C	1.688811	-4.945419	0.099359
C	-2.643951	5.079366	-0.627255	H	-0.047734	-5.575014	-1.000695
H	-2.516215	5.998378	-0.068581	H	2.435253	-1.688278	0.612841
C	-2.088565	4.756717	2.329844	H	3.324445	-3.957709	1.134741
H	-1.769416	5.701338	1.906565	H	2.022183	-5.957501	0.306123
C	-2.814786	-0.917252	0.754301	C	-1.108783	-3.109045	-1.703143
N	-2.926765	2.531102	2.020924	C	-2.087487	-4.058148	-2.019624
C	-3.841816	-3.159193	0.679713	C	-2.295912	-1.443877	-2.799713
C	-1.813509	-1.495494	1.524207	C	-3.198483	-3.658544	-2.760774
C	-3.813328	-1.799118	0.360641	H	-2.006071	-5.082166	-1.675620
C	-2.991745	3.927292	-2.707205	C	-3.311892	-2.327398	-3.160327
H	-3.120673	3.909593	-3.783457	H	-2.325359	-0.392963	-3.074114
C	-2.377388	3.302277	4.224473	H	-3.972688	-4.377341	-3.007357
H	-2.318106	3.092599	5.287248	H	-4.166093	-1.977243	-3.729559
C	-2.795400	-3.677126	1.442487	N	0.824520	-2.358640	-0.463782
C	-2.999425	2.736524	-1.986420	N	-1.228482	-1.826097	-2.093613
H	-3.119166	1.779357	-2.480155	Cu	0.326348	-0.543994	-1.243314
C	-1.765371	-2.845630	1.881185	C	4.120710	0.334877	0.436021
C	-2.833505	2.326702	3.338364	C	4.395832	-0.189204	-0.831383
H	-3.131212	1.341218	3.687961	C	4.585480	-0.384720	1.542457
C	-2.798978	5.118956	-2.011465	C	5.075769	-1.390696	-0.996496
H	-2.777631	6.069661	-2.535066	C	5.255877	-1.594423	1.398460
C	-1.997285	4.538639	3.702713	C	5.500588	-2.098101	0.124049
H	-1.624310	5.322967	4.354394	F	4.347430	0.066660	2.781525
C	3.481614	2.723331	-0.048451	F	3.937851	0.430757	-1.926380
C	3.241993	1.510311	0.598653	F	5.279435	-1.890973	-2.220882
C	2.059636	1.399777	1.335914	F	6.114893	-3.275347	-0.018413
C	1.151346	2.444208	1.431578	F	5.644693	-2.290971	2.473770
C	1.413896	3.638985	0.770250	Cl	-0.465837	-3.503773	2.845470
C	2.585037	3.782218	0.033529	Cl	-5.128817	-4.211675	0.140122
F	4.583106	2.875304	-0.797260	F	0.006068	2.280735	2.104743
F	0.522648	4.635596	0.802388	F	2.816735	4.919498	-0.633990

4 [Cu(bipy)Rf]

Charge	0
Spin State	Singlet
Electronic Energy, BS1 (a.u.)	-2141.367447
Thermal and entropic correction, BS1 (a.u.)	0.151550
Electronic Energy, BS2 (a.u.)	-3585.103214
Gibbs Energy, BS2 (a.u.)	-3584.951617
Number of Imaginary Frequencies	0

Molecular Geometry in Cartesian Coordinates

C	-4.769165	1.517032	0.057044	H	-5.554942	-3.521018	-0.119056
C	-3.603724	0.744604	0.014237	H	-3.268405	-4.570866	-0.078444
C	-2.282408	2.657652	0.022737	N	-2.382393	1.322132	-0.000307
C	-3.396657	3.493741	0.062093	N	-2.382184	-1.322257	0.002607
C	-4.660933	2.906648	0.080480	Cu	-0.728211	0.000530	0.001732
H	-5.746618	1.050285	0.075667	C	1.195503	0.000156	0.000606
H	-1.271540	3.052970	0.008678	C	1.949477	-1.166844	0.027533
H	-3.269349	4.570588	0.078913	C	1.949673	1.167019	-0.026957
H	-5.555809	3.520351	0.113379	C	3.346497	-1.209937	0.028282
C	-3.603569	-0.744948	-0.015151	C	3.346693	1.209865	-0.029005
C	-4.768779	-1.517554	-0.060940	C	4.040757	-0.000102	-0.000682
C	-2.281933	-2.657749	-0.020020	F	1.305651	2.366987	-0.054137
C	-4.660260	-2.907158	-0.083900	F	1.305275	-2.366687	0.055417
H	-5.746253	-1.050965	-0.082186	Cl	4.228961	-2.719633	0.063505
C	-3.395940	-3.494036	-0.062103	F	5.381352	-0.000222	-0.001293
H	-1.271036	-3.052902	-0.003497	Cl	4.229404	2.719399	-0.06498

Pf-Pf

Charge	0
Spin State	Singlet
Electronic Energy, BS1 (a.u.)	-1455.573083
Thermal and entropic correction, BS1 (a.u.)	0.053984
Electronic Energy, BS2 (a.u.)	-1456.239228
Gibbs Energy, BS2 (a.u.)	-1456.185244
Number of Imaginary Frequencies	0

Molecular Geometry in Cartesian Coordinates

F	0.825524	2.117247	-1.067703	C	-1.466628	-1.070074	-0.530070
F	0.824509	-2.116999	1.066793	C	-2.856958	-1.082447	-0.529532
F	4.889466	-0.000376	0.000398	C	-3.553970	-0.000033	-0.000161
C	0.738486	0.000325	-0.000025	C	-2.857249	1.082482	0.529382
C	2.856872	-1.082516	0.529617	F	-0.825350	2.117317	1.067472
C	1.467009	1.070452	-0.530109	F	-4.889477	-0.000227	-0.000135
C	1.466544	-1.070143	0.529972	F	-0.824675	-2.116988	-1.066930
C	3.553974	-0.000136	0.000277	F	3.527012	2.120449	-1.044743
C	2.857350	1.082408	-0.529340	F	3.526080	-2.120883	1.044950
C	-1.466914	1.070507	0.529962	F	-3.526292	-2.120927	-1.044825
C	-0.738453	0.000368	-0.000199	F	-3.526839	2.120592	1.044872

Reaction between [Cu(bipy)Pf] and *p*-substituted Ar-I (*p*-X-Ar-I; X = H, OMe, Br, NO2)

(Gibbs energy profile in Figure S20)

PhH-I

Charge	0
Spin State	Singlet

Electronic Energy, BS1 (a.u.)	-243.080884
Thermal and entropic correction, BS1 (a.u.)	0.058391
Electronic Energy, BS2 (a.u.)	-529.548381
Gibbs Energy, BS2 (a.u.)	-529.489990
Number of Imaginary Frequencies	0

Molecular Geometry in Cartesian Coordinates

C	-1.260003	-1.218455	-0.000000	I	1.564232	0.000000	-0.000001
C	-2.657986	-1.208516	0.000000	H	-4.443947	0.000000	0.000002
C	-3.357904	0.000000	0.000002	H	-3.195490	2.152567	0.000003
C	-2.657986	1.208516	0.000002	H	-0.718026	2.157733	0.000002
C	-1.260003	1.218455	0.000001	H	-3.195490	-2.152567	-0.000000
C	-0.578337	0.000000	0.000000	H	-0.718026	-2.157733	-0.000001

PhBr-I

Charge	0
Spin State	Singlet
Electronic Energy, BS1 (a.u.)	-2813.886115
Thermal and entropic correction, BS1 (a.u.)	0.045134
Electronic Energy, BS2 (a.u.)	-3103.131816
Gibbs Energy, BS2 (a.u.)	-3103.086682
Number of Imaginary Frequencies	0

Molecular Geometry in Cartesian Coordinates

C	-0.141984	1.215807	-0.000001	I	-2.964340	0.000000	-0.000001
C	1.254617	1.215390	-0.000000	H	1.795759	-2.154856	0.000002
C	1.936978	0.000000	0.000001	H	-0.677390	-2.158576	0.000001
C	1.254617	-1.215390	0.000001	H	1.795759	2.154856	-0.000001
C	-0.141984	-1.215807	0.000001	H	-0.677390	2.158576	-0.000002
C	-0.828175	0.000000	-0.000000	Br	3.853396	0.000000	0.000002

PhOMe-I

Charge	0
Spin State	Singlet
Electronic Energy, BS1 (a.u.)	-357.609848
Thermal and entropic correction, BS1 (a.u.)	0.087665
Electronic Energy, BS2 (a.u.)	-644.123883
Gibbs Energy, BS2 (a.u.)	-644.036218
Number of Imaginary Frequencies	0

Molecular Geometry in Cartesian Coordinates

C	-0.190523	0.073919	-0.000258	H	2.285924	2.401801	0.000154
C	1.797813	1.432281	-0.000160	H	0.120780	-2.062848	-0.000629
C	0.585328	-1.082768	-0.000541	H	2.568286	-1.896345	-0.000852
C	0.410558	1.337231	-0.000090	O	3.936516	0.483826	-0.000374
C	2.593599	0.274873	-0.000398	C	4.797949	-0.654382	0.000973
C	1.981004	-0.985846	-0.000646	H	4.650257	-1.271280	0.896281
I	-2.325434	-0.077759	0.000129	H	4.651871	-1.272161	-0.894021
H	-0.191228	2.239476	0.000260	H	5.815644	-0.259895	0.001711

$PhNO_2$-I

Charge	0
Spin State	Singlet
Electronic Energy, BS1 (a.u.)	-447.582194
Thermal and entropic correction, BS1 (a.u.)	0.056779

Electronic Energy, BS2 (a.u.)	-734,143699
Gibbs Energy, BS2 (a.u.)	-734,086920
Number of Imaginary Frequencies	0

Molecular Geometry in Cartesian Coordinates

C	-0.419466	-0.000001	0.000041	H	-0.274751	-2.159813	-0.000394
C	1.657296	-1.220379	-0.000143	H	2.212443	-2.150072	-0.000322
C	0.264958	1.220110	0.000281	H	-0.274741	2.159810	0.000467
C	0.264955	-1.220117	-0.000177	H	2.212452	2.150055	0.000401
C	2.332575	-0.000008	0.000062	N	3.799432	0.000002	-0.000025
C	1.657301	1.220364	0.000260	O	4.378572	1.088590	-0.000616
I	-2.548573	0.000002	-0.000019	O	4.378585	-1.088577	0.000499

IO-PhH

Charge	0
Spin State	Singlet
Electronic Energy, BS1 (a.u.)	-1663.729967
Thermal and entropic correction, BS1 (a.u.)	0.235098
Electronic Energy, BS2 (a.u.)	-3393.963093
Gibbs Energy, BS2 (a.u.)	-3393.727995
Number of Imaginary Frequencies	0

Molecular Geometry in Cartesian Coordinates

Cu	-0.049294	0.750494	1.294748	H	-0.606294	3.304127	-0.908988
C	2.300531	2.019484	-0.125539	C	4.592213	0.228880	2.312856
N	1.887738	0.536722	1.731958	H	5.648222	0.118112	2.538777
C	2.818372	1.189188	0.994691	C	2.504751	3.119999	-2.254386
C	4.182351	1.058308	1.271550	H	3.092017	3.401928	-3.122921
H	4.912735	1.606843	0.688745	C	-2.468426	-0.848210	1.225826
C	3.086992	2.377220	-1.227790	C	-1.928582	0.426122	1.108608
H	4.121763	2.063082	-1.299562	C	-2.821079	1.385015	0.647108
C	0.407089	-1.462509	-1.539804	C	-4.144245	1.123754	0.301893
N	1.000643	2.362700	-0.039950	C	-4.625984	-0.176251	0.425546
C	-0.985970	0.203378	-2.572741	C	-3.779004	-1.176160	0.892643
C	-0.694366	-2.298082	-1.347644	F	-1.685700	-1.885630	1.646566
C	0.279739	-0.212658	-2.148752	F	-5.895990	-0.464840	0.086739
C	3.629061	-0.447588	3.060481	F	-2.410986	2.681358	0.488645
H	3.901914	-1.105536	3.878040	I	2.337587	-2.114195	-0.882599
C	1.161045	3.481487	-2.156261	F	-4.231941	-2.445494	0.971617
H	0.670950	4.056339	-2.934913	F	-4.963495	2.095044	-0.152265
C	-2.099637	-0.618754	-2.393068	H	1.140403	0.431369	-2.285944
C	2.288572	-0.260115	2.736588	H	-1.092718	1.179354	-3.036839
H	1.498560	-0.765135	3.281312	H	-3.081026	-0.285953	-2.716882
C	-1.950540	-1.865759	-1.781740	H	-0.584650	-3.259658	-0.859520
C	0.447853	3.074245	-1.028479	H	-2.814525	-2.502700	-1.616560

IO-PhBr

Charge	0
Spin State	Singlet
Electronic Energy, BS1 (a.u.)	-4234.537814
Thermal and entropic correction, BS1 (a.u.)	0.222859
Electronic Energy, BS2 (a.u.)	-5967.548221
Gibbs Energy, BS2 (a.u.)	-5967.325262

Number of Imaginary Frequencies 0

Molecular Geometry in Cartesian Coordinates

Cu	-0.702691	-0.954838	1.405600	H	0.354692	-3.166133	-0.981530
C	-2.671366	-1.959340	-0.661872	C	-5.463793	-0.531758	1.475840
N	-2.691528	-0.772522	1.438626	H	-6.545942	-0.447634	1.488765
C	-3.431537	-1.298783	0.432904	C	-2.392904	-2.721347	-2.926171
C	-4.826008	-1.199136	0.432572	H	-2.773792	-2.857838	-3.933608
H	-5.406421	-1.650864	-0.363037	C	1.700651	0.566991	1.982269
C	-3.195090	-2.132007	-1.949356	C	1.176047	-0.674059	1.646880
H	-4.194600	-1.791845	-2.194438	C	2.129015	-1.618638	1.285330
C	-0.532098	1.674561	-1.102589	C	3.497594	-1.373087	1.236751
N	-1.415823	-2.334935	-0.350538	C	3.964128	-0.104302	1.566312
C	1.003731	0.105239	-2.097081	C	3.056246	0.879635	1.942619
C	0.530406	2.442001	-0.622485	F	0.865712	1.591082	2.330847
C	-0.307704	0.508342	-1.836032	F	5.277451	0.174541	1.496207
C	-4.694794	0.016233	2.501762	F	1.737427	-2.880182	0.924240
H	-5.148537	0.545483	3.332283	I	-2.535215	2.293522	-0.704131
C	-1.096820	-3.117940	-2.595561	F	3.501668	2.125172	2.214546
H	-0.441360	-3.578013	-3.327474	F	4.374922	-2.322563	0.853589
C	2.062780	0.875278	-1.621898	H	-1.132182	-0.093011	-2.199799
C	-3.312252	-0.132513	2.443755	H	1.184430	-0.804509	-2.657615
H	-2.665874	0.277020	3.212161	H	0.353771	3.337632	-0.038581
C	1.840923	2.038648	-0.886342	Br	3.860277	0.321106	-1.983141
C	-0.651097	-2.899615	-1.291579	H	2.670998	2.617969	-0.498320

IO-PhOMe

Charge	0
Spin State	Singlet
Electronic Energy, BS1 (a.u.)	-1778.262725
Thermal and entropic correction, BS1 (a.u.)	0.263358
Electronic Energy, BS2 (a.u.)	-3508.540745
Gibbs Energy, BS2 (a.u.)	-3508.277387
Number of Imaginary Frequencies	0

Molecular Geometry in Cartesian Coordinates

Cu	-0.560124	0.428518	-1.406089	C	2.456311	0.632447	2.012924
C	-3.026354	1.814846	-0.417832	C	-2.804304	-1.443376	-2.059277
N	-2.500280	-0.236919	-1.559594	H	-1.980264	-1.988254	-2.507581
C	-3.467086	0.502611	-0.969862	C	2.483535	-0.763527	1.895040
C	-4.779425	0.028598	-0.873479	C	-1.213084	3.221507	-0.059332
H	-5.550827	0.619529	-0.395352	H	-0.143844	3.357829	-0.184799
C	-3.891100	2.723215	0.204399	C	-5.090574	-1.226849	-1.392608
H	-4.949937	2.514699	0.300076	H	-6.104353	-1.608335	-1.320876
C	0.068253	-0.805727	1.884783	C	-3.370868	3.915006	0.706790
N	-1.708710	2.075321	-0.539465	H	-4.026338	4.630205	1.194084
C	1.222306	1.299738	2.069537	C	1.854640	-1.164949	-1.675061
C	1.285086	-1.480967	1.825066	C	1.327593	0.110899	-1.520730
C	0.029957	0.587126	2.007814	C	2.271075	1.094889	-1.257317
C	-4.087587	-1.981205	-1.999479	C	3.636062	0.856202	-1.135940
H	-4.285963	-2.963204	-2.414657	C	4.103915	-0.444955	-1.285202
C	-2.007098	4.175099	0.575994	C	3.207104	-1.472689	-1.558638
H	-1.564482	5.089941	0.954990	F	1.022523	-2.222712	-1.919218

F	5.414642	-0.712110	-1.113993	H	1.317057	-2.559738	1.717550
F	1.874658	2.390049	-1.063596	H	3.421461	-1.302430	1.840250
I	-1.762812	-1.904066	1.774213	O	3.558611	1.425477	2.070731
F	3.663151	-2.737120	-1.681334	C	4.844622	0.806757	2.115288
F	4.508399	1.838656	-0.828085	H	4.940679	0.151266	2.989855
H	-0.914626	1.116482	2.042828	H	5.060393	0.233968	1.208241
H	1.215182	2.381853	2.148955	H	5.564322	1.623026	2.196066

IO-PhNO$_2$

Charge	0
Spin State	Singlet
Electronic Energy, BS1 (a.u.)	-1868.235707
Thermal and entropic correction, BS1 (a.u.)	0.234105
Electronic Energy, BS2 (a.u.)	-3598.561736
Gibbs Energy, BS2 (a.u.)	-3598.327631
Number of Imaginary Frequencies	0

Molecular Geometry in Cartesian Coordinates

Cu	0.279495	-0.598093	1.296616	C	-4.275937	-0.277265	2.751758
C	-2.093603	-2.200821	0.314017	H	-5.296985	-0.250545	3.119130
N	-1.654621	-0.375765	1.821207	C	-2.407243	-3.680063	-1.555853
C	-2.575297	-1.250505	1.350853	H	-3.054672	-4.185834	-2.265578
C	-3.895686	-1.230169	1.809673	C	2.657839	1.081924	1.268284
H	-4.613303	-1.956048	1.446061	C	2.150662	-0.203639	1.120550
C	-2.954887	-2.847891	-0.579940	C	3.088280	-1.141664	0.706514
H	-4.025685	-2.686153	-0.536372	C	4.421621	-0.849983	0.432467
C	-1.535810	1.284945	-1.390423	C	4.869670	0.457523	0.592024
N	-0.756949	-2.355569	0.248369	C	3.978302	1.438390	1.016439
C	0.127178	-0.180091	-2.328446	F	1.828218	2.103908	1.641924
C	-0.555643	2.219621	-1.039008	F	6.149217	0.773277	0.327494
C	-1.207961	0.092617	-2.042494	F	2.715213	-2.444793	0.519845
C	-3.329036	0.635780	3.213754	I	-3.568075	1.674287	-0.897465
H	-3.582455	1.399564	3.940510	F	4.404763	2.711059	1.148932
C	-1.023029	-3.842197	-1.614626	F	5.281142	-1.799455	0.011506
H	-0.558104	-4.478373	-2.360236	H	-1.972229	-0.628598	-2.305017
C	1.098375	0.752540	-1.970656	H	0.412208	-1.107244	-2.808126
C	-2.028855	0.545283	2.724649	H	-0.819763	3.137666	-0.527406
H	-1.250896	1.225694	3.053502	H	1.558230	2.647586	-1.058404
C	0.777192	1.953357	-1.339147	N	2.505632	0.439770	-2.227915
C	-0.236599	-3.155348	-0.688938	O	3.338166	1.332830	-2.057840
H	0.846461	-3.227944	-0.696416	O	2.786487	-0.701753	-2.602849

TS_OA-PhH

Charge	0
Spin State	Singlet
Electronic Energy, BS1 (a.u.)	-1663.693335
Thermal and entropic correction, BS1 (a.u.)	0.235349
Electronic Energy, BS2 (a.u.)	-3393.927444
Gibbs Energy, BS2 (a.u.)	-3393.692095
Number of Imaginary Frequencies	1
Imaginary Frequencies (cm^{-1})	-176.0i

Molecular Geometry in Cartesian Coordinates

Cu	0.349530	0.155147	-0.085418
C	2.630435	-1.358367	1.303241
N	1.613379	-1.409265	-0.875656
C	2.629688	-1.845636	-0.104754
C	3.595116	-2.722241	-0.616959
H	4.421049	-3.062285	-0.004206
C	3.520930	-1.841883	2.270619
H	4.275238	-2.575940	2.015525
C	-0.320614	2.159389	0.004732
N	1.686199	-0.444680	1.615062
C	-1.174836	3.335641	1.923226
C	-1.498100	2.419894	-0.709160
C	-0.120920	2.665015	1.292672
C	2.418935	-2.712879	-2.716319
H	2.293957	-3.033975	-3.744737
C	2.424346	-0.457584	3.899747
H	2.298257	-0.083039	4.909815
C	-2.380095	3.548196	1.252314
C	1.503643	-1.835994	-2.139474
H	0.647461	-1.457990	-2.688885
C	-2.531315	3.097673	-0.066470
C	1.580570	-0.021885	2.880087
H	0.783189	0.686870	3.075829
C	3.488149	-3.154230	-1.937006
H	4.230414	-3.831223	-2.348494
C	3.419282	-1.380006	3.580609
H	4.101901	-1.746215	4.341220
C	-2.181915	-0.909039	-1.194459
C	-1.478241	-0.661523	-0.028930
C	-2.169770	-0.926863	1.139944
C	-3.477705	-1.405294	1.175309
C	-4.140339	-1.639014	-0.026600
C	-3.488977	-1.385497	-1.231300
F	-1.600888	-0.641663	-2.410120
F	-5.403710	-2.103826	-0.024772
F	-1.578635	-0.695853	2.351262
I	1.702171	2.082541	-1.337243
F	-4.135591	-1.605741	-2.395228
F	-4.115130	-1.644194	2.340536
H	0.827128	2.524371	1.796513
H	-1.037457	3.703796	2.936445
H	-3.190953	4.079010	1.741995
H	-1.619810	2.044956	-1.718381
H	-3.462870	3.269594	-0.598983

TS_OA-PhBr

Charge	0
Spin State	Singlet
Electronic Energy, BS1 (a.u.)	-4234.499491
Thermal and entropic correction, BS1 (a.u.)	0.222118
Electronic Energy, BS2 (a.u.)	-5967.512112
Gibbs Energy, BS2 (a.u.)	-5967.289994
Number of Imaginary Frequencies	1
Imaginary Frequencies (cm^{-1})	-146.21i

Molecular Geometry in Cartesian Coordinates

Cu	-0.901488	-0.038058	-0.168436
C	-3.345844	-0.131209	1.714178
N	-2.859973	0.812745	-0.446519
C	-3.814894	0.525092	0.461841
C	-5.156078	0.851072	0.222970
H	-5.927506	0.593807	0.938529
C	-4.162305	-0.273489	2.843965
H	-5.185305	0.082273	2.840684
C	0.633331	-1.399082	-0.684412
N	-2.062186	-0.547747	1.720039
C	2.387671	-2.211375	0.760927
C	1.583529	-0.898891	-1.586989
C	1.028923	-2.111426	0.452738
C	-4.496025	1.822226	-1.876797
H	-4.717626	2.339520	-2.803992
C	-2.305753	-1.274960	3.995013
H	-1.850947	-1.723630	4.871529
C	3.326763	-1.650140	-0.102216
C	-3.187687	1.451560	-1.576645
H	-2.363187	1.670323	-2.247132
C	2.936021	-1.007080	-1.281645
C	-1.558038	-1.091727	2.833400
H	-0.513026	-1.378959	2.788682
C	-5.497127	1.502937	-0.959918
H	-6.533030	1.759085	-1.159539
C	-3.635954	-0.858742	3.993173
H	-4.255061	-0.974995	4.877350
C	0.646644	2.337488	-1.019596
C	0.352096	1.501441	0.042852
C	1.099801	1.718553	1.187067
C	2.089136	2.693600	1.291950
C	2.346357	3.509605	0.193562
C	1.620184	3.331166	-0.981553
F	-0.007024	2.174232	-2.215573
F	3.293278	4.463193	0.264854
F	0.906981	0.930823	2.287793

I	-1.413031	-2.174529	-1.688395	H	2.700386	-2.730517	1.660438
F	1.876682	4.118760	-2.046673	H	1.268942	-0.376695	-2.482685
F	2.801132	2.863328	2.425006	Br	5.191833	-1.788211	0.315351
H	0.290975	-2.560586	1.105353	H	3.676183	-0.586242	-1.953419

TS_OA-PhOMe

Charge	0
Spin State	Singlet
Electronic Energy, BS1 (a.u.)	-1778.221513
Thermal and entropic correction, BS1 (a.u.)	0.265004
Electronic Energy, BS2 (a.u.)	-3508.499541
Gibbs Energy, BS2 (a.u.)	-3508.234537
Number of Imaginary Frequencies	1
Imaginary Frequencies (cm^{-1})	-124.3i

Molecular Geometry in Cartesian Coordinates

Cu	-0.698750	0.067286	-0.190165	H	-5.618016	3.479619	-0.128158
C	-3.242296	-0.187915	1.570519	C	-3.942935	-1.756991	3.259941
N	-2.397763	1.350722	-0.084702	H	-4.693794	-2.133475	3.947978
C	-3.438708	1.008267	0.703996	C	1.423082	2.140453	-0.104504
C	-4.616792	1.766598	0.704462	C	0.837443	1.114246	0.616174
H	-5.452488	1.496253	1.338233	C	1.655804	0.523106	1.565395
C	-4.224457	-0.656364	2.453928	C	2.997782	0.850380	1.738355
H	-5.192906	-0.175568	2.520329	C	3.546543	1.867618	0.964482
C	0.675491	-1.214023	-1.023125	C	2.751040	2.532139	0.035604
N	-2.039922	-0.790999	1.478101	F	0.722459	2.752416	-1.113039
C	2.246486	-2.652418	0.087761	F	4.853822	2.171471	1.083320
C	1.710006	-0.586279	-1.719628	F	1.188155	-0.511501	2.324577
C	0.926350	-2.312772	-0.197568	I	-1.545025	-1.325321	-2.247789
C	-3.623191	3.233152	-0.921129	F	3.290283	3.509014	-0.723357
H	-3.649609	4.100477	-1.571571	F	3.789145	0.181443	2.602079
C	-2.687508	-2.357107	3.177259	H	0.110437	-2.850704	0.268614
H	-2.421229	-3.207062	3.796315	H	2.473475	-3.456182	0.781391
C	3.304368	-1.927076	-0.480330	H	1.498146	0.227928	-2.402864
C	-2.484868	2.433433	-0.867954	H	3.831879	-0.379206	-1.910836
H	-1.603054	2.657432	-1.457331	O	4.556416	-2.274434	-0.074261
C	3.030173	-0.923635	-1.425991	C	5.620666	-1.357219	-0.333384
C	-1.768220	-1.833676	2.269513	H	5.847250	-1.282014	-1.404043
H	-0.772525	-2.252821	2.172816	H	5.394233	-0.359571	0.063850
C	-4.709332	2.885571	-0.119140	H	6.494378	-1.759513	0.183143

TS_OA-PhNO$_2$

Charge	0
Spin State	Singlet
Electronic Energy, BS1 (a.u.)	-1868.198873
Thermal and entropic correction, BS1 (a.u.)	0.233430
Electronic Energy, BS2 (a.u.)	-3598.527820
Gibbs Energy, BS2 (a.u.)	-3598.294390
Number of Imaginary Frequencies	1
Imaginary Frequencies (cm^{-1})	-138.3i

Molecular Geometry in Cartesian Coordinates

Cu	-0.715094	-0.026084	-0.171626	C	-3.203937	0.168496	1.603091

N	-2.524247	1.057342	-0.529130	H	-5.963836	2.637133	-1.275153
C	-3.530320	0.920615	0.359313	C	-3.707570	-0.645592	3.814408
C	-4.788438	1.482909	0.109616	H	-4.396148	-0.758957	4.645990
H	-5.600312	1.366666	0.816874	C	1.153854	2.112364	-0.971636
C	-4.106534	0.033833	2.665747	C	0.726200	1.316016	0.076397
H	-5.102011	0.456768	2.608738	C	1.452645	1.438736	1.248325
C	0.653509	-1.555805	-0.658577	C	2.545550	2.290623	1.392575
N	-1.961429	-0.355647	1.669036	C	2.935616	3.072784	0.308952
C	2.315855	-2.487845	0.824695	C	2.236207	2.983385	-0.892428
C	1.657397	-1.176294	-1.574977	F	0.527647	2.028120	-2.188175
C	0.981868	-2.277043	0.505172	F	3.983932	3.907949	0.419710
C	-3.938815	2.346849	-1.969106	F	1.130883	0.676225	2.334917
H	-4.053786	2.903687	-2.892600	I	-1.409606	-2.146339	-1.637426
C	-2.415011	-1.162418	3.883727	F	2.620503	3.736984	-1.942457
H	-2.057469	-1.685347	4.764111	F	3.230727	2.372347	2.550560
C	3.308484	-2.036492	-0.050974	H	0.200285	-2.636293	1.162007
C	-2.717719	1.756213	-1.654814	H	2.592552	-3.002718	1.736601
H	-1.855581	1.841633	-2.307634	H	1.391712	-0.655923	-2.486953
C	2.985371	-1.395932	-1.257099	H	3.775300	-1.071558	-1.923269
C	-1.577800	-0.988343	2.783543	N	4.703162	-2.256183	0.284234
H	-0.558127	-1.357268	2.790137	O	5.565935	-1.855992	-0.508027
C	-4.992798	2.198093	-1.068179	O	4.967432	-2.832704	1.347433

P1-PhH

Charge	0
Spin State	Singlet
Electronic Energy, BS1 (a.u.)	-1663.810820
Thermal and entropic correction, BS1 (a.u.)	0.240302
Electronic Energy, BS2 (a.u.)	-3394.036215
Gibbs Energy, BS2 (a.u.)	-3393.795913
Number of Imaginary Frequencies	0

Molecular Geometry in Cartesian Coordinates

Cu	-0.269236	-1.307231	-0.827172	H	2.072730	-3.107195	0.248493
C	1.475788	0.804999	-1.819065	C	-4.014298	1.643188	0.013945
N	1.836940	-1.306377	-0.719392	C	-0.741539	1.335868	-2.281676
C	2.412482	-0.200878	-1.240522	H	-1.776105	1.017368	-2.225926
C	3.799225	-0.020106	-1.195100	C	4.591935	-1.000272	-0.600555
H	4.259993	0.867384	-1.610384	H	5.668576	-0.870802	-0.552141
C	1.900890	2.034351	-2.332172	C	0.955594	2.926488	-2.834615
H	2.949727	2.302955	-2.331941	H	1.272033	3.886661	-3.229811
C	-1.817868	2.038576	0.972879	C	-0.325995	0.308170	2.047936
N	0.162432	0.477911	-1.788866	C	-0.485110	1.526558	1.373144
C	-3.367539	3.881089	0.655471	C	0.696774	2.198915	1.036773
C	-2.776302	1.161122	0.437056	C	1.956459	1.695346	1.339461
C	-2.127382	3.404214	1.079545	C	2.073017	0.479185	2.002245
C	3.988204	-2.138115	-0.068638	C	0.924499	-0.219705	2.352177
H	4.569260	-2.920314	0.407257	F	-1.400373	-0.405012	2.424458
C	-0.391406	2.573873	-2.816044	F	3.281088	-0.037759	2.253893
H	-1.160420	3.238379	-3.193888	F	0.650442	3.346947	0.336478
C	-4.314066	3.003865	0.119950	I	-2.040473	-3.014858	-0.311736
C	2.601785	-2.248299	-0.152528	F	1.027863	-1.415874	2.947679

F	3.059380	2.343152	0.933445	H	-5.278379	3.378208	-0.211860
H	-1.397591	4.091005	1.494239	H	-2.544272	0.106695	0.331180
H	-3.593783	4.939725	0.745473	H	-4.740971	0.952676	-0.404485

P1-PhBr

Charge	0
Spin State	Singlet
Electronic Energy, BS1 (a.u.)	-4234.615756
Thermal and entropic correction, BS1 (a.u.)	0.227872
Electronic Energy, BS2 (a.u.)	-5967.618666
Gibbs Energy, BS2 (a.u.)	-5967.390794
Number of Imaginary Frequencies	0

Molecular Geometry in Cartesian Coordinates

Cu	0.074851	-1.743636	0.562686	H	1.361923	-0.316336	3.265010
C	-1.696228	-0.002782	2.125476	C	-4.720238	-1.541703	0.427463
N	-1.940007	-1.651645	0.386129	H	-5.804078	-1.491913	0.444915
C	-2.573459	-0.808895	1.236072	C	-1.219372	1.869940	3.554572
C	-3.968331	-0.733677	1.279150	H	-1.508757	2.811444	4.010797
H	-4.458604	-0.065620	1.977871	C	-1.526655	0.461389	-2.008391
C	-2.102323	1.203918	2.705381	C	-0.936954	1.429545	-1.183561
H	-3.069408	1.632536	2.468684	C	-1.828338	2.260888	-0.490108
C	0.532475	1.516417	-1.011834	C	-3.208207	2.125866	-0.587225
N	-0.460332	-0.507179	2.325403	C	-3.752508	1.160786	-1.426170
C	2.463175	1.788427	0.445078	C	-2.903044	0.331202	-2.148365
C	1.401917	1.311861	-2.095950	F	-0.764380	-0.443942	-2.648167
C	1.084677	1.765442	0.255109	F	-5.077651	0.996922	-1.499658
C	-4.061090	-2.413139	-0.438214	F	-1.370574	3.219813	0.335157
H	-4.605606	-3.057015	-1.119531	I	2.400833	-2.450202	-0.023678
C	0.038756	1.318395	3.799161	F	-3.414359	-0.639378	-2.918549
H	0.750315	1.801753	4.459879	F	-4.014879	2.901577	0.153241
C	3.301095	1.566481	-0.645492	H	0.437744	1.928649	1.105542
C	-2.668491	-2.437110	-0.423601	H	2.872816	1.962033	1.433513
H	-2.104088	-3.085849	-1.084408	H	1.003619	1.127448	-3.086673
C	2.783071	1.335518	-1.918662	Br	5.197810	1.570051	-0.386747
C	0.380456	0.133613	3.147021	H	3.442588	1.169813	-2.762844

P1-PhOMe

Charge	0
Spin State	Singlet
Electronic Energy, BS1 (a.u.)	-1778.339131
Thermal and entropic correction, BS1 (a.u.)	0.268487
Electronic Energy, BS2 (a.u.)	-3508.613478
Gibbs Energy, BS2 (a.u.)	-3508.344991
Number of Imaginary Frequencies	0

Molecular Geometry in Cartesian Coordinates

Cu	-1.381949	-0.733665	-1.010819	N	0.623526	-0.577886	-1.594147
C	-0.050406	1.718600	-1.845270	C	0.955494	0.643655	-2.068877

C	2.183050	0.865734	-2.701788	H	-0.531335	5.078910	-1.811004
H	2.430945	1.842085	-3.099735	C	2.247638	-0.898147	2.062746
C	0.247747	3.078651	-1.976375	C	1.799629	0.328797	1.548618
H	1.245109	3.402044	-2.247979	C	2.699076	0.985115	0.694815
C	0.448618	0.864436	1.830325	C	3.936891	0.456140	0.351028
N	-1.277376	1.304210	-1.458919	C	4.333918	-0.771119	0.867712
C	-1.065139	2.745730	2.133892	C	3.481768	-1.448480	1.732454
C	-0.658081	0.004579	1.909741	F	1.479404	-1.601890	2.914698
C	0.217806	2.248508	1.959772	F	5.515647	-1.299075	0.526446
C	2.747725	-1.437406	-2.302325	F	2.365202	2.156873	0.116989
H	3.428903	-2.279385	-2.358655	I	-3.100230	-2.506626	-0.490821
C	-2.012641	3.583082	-1.328444	F	3.854579	-2.628352	2.248578
H	-2.813127	4.281226	-1.109985	F	4.729280	1.105436	-0.515207
C	-2.165623	1.873906	2.181787	H	1.049125	2.942122	1.916228
C	1.497007	-1.588310	-1.707437	H	-1.241413	3.813119	2.219142
H	1.172973	-2.540777	-1.300337	H	-0.525335	-1.064483	1.796531
C	-1.952390	0.493112	2.086637	H	-2.775724	-0.209299	2.103790
C	-2.229339	2.212321	-1.206266	O	-3.384020	2.459904	2.304972
H	-3.188777	1.815769	-0.890827	C	-4.539055	1.617937	2.288237
C	3.088738	-0.186640	-2.816378	H	-4.604947	1.044079	1.355814
H	4.049628	-0.027842	-3.294915	H	-4.545963	0.926407	3.139509
C	-0.747648	4.019107	-1.718695	H	-5.397945	2.287224	2.362392

P1-PhNO$_2$

Charge	0
Spin State	Singlet
Electronic Energy, BS1 (a.u.)	-1868.311544
Thermal and entropic correction, BS1 (a.u.)	0.240216
Electronic Energy, BS2 (a.u.)	-3598.629967
Gibbs Energy, BS2 (a.u.)	-3598.389751
Number of Imaginary Frequencies	0

Molecular Geometry in Cartesian Coordinates

Cu	0.527783	-1.595804	0.550360	H	-1.478718	-3.213485	-1.053904
C	-1.421205	-0.070428	2.126107	C	2.708730	1.596258	-2.076993
N	-1.476752	-1.757801	0.404954	C	0.633263	0.350595	3.114041
C	-2.199457	-0.997239	1.261832	H	1.664836	0.030207	3.228447
C	-3.591278	-1.105026	1.330702	C	-4.246439	-2.011844	0.498793
H	-4.151612	-0.501534	2.035300	H	-5.326915	-2.104388	0.536918
C	-1.970000	1.089353	2.684494	C	-1.167758	1.881797	3.504974
H	-2.985255	1.389550	2.451003	H	-1.568111	2.790483	3.943381
C	0.482117	1.621039	-1.106760	C	-1.503527	0.370219	-2.018245
N	-0.129327	-0.410150	2.318984	C	-0.978187	1.403665	-1.229418
C	2.416978	2.056597	0.297720	C	-1.914385	2.154910	-0.503785
C	1.332098	1.469909	-2.216969	C	-3.276176	1.880329	-0.533318
C	1.042957	1.931049	0.145234	C	-3.757445	0.849605	-1.332267
C	-3.494770	-2.796562	-0.374413	C	-2.864573	0.099826	-2.088941
H	-3.962668	-3.511514	-1.041548	F	-0.684968	-0.462749	-2.683873
C	0.152958	1.499405	3.742863	F	-5.059536	0.551137	-1.338765
H	0.805553	2.084833	4.381511	F	-1.516768	3.166275	0.287234
C	3.233343	1.880308	-0.817320	I	2.935712	-2.031469	0.021501
C	-2.111028	-2.638329	-0.386593	F	-3.311484	-0.925134	-2.826296

F	-4.121132	2.580591	0.237773	H	3.370312	1.469256	-2.924254
H	0.405255	2.054136	1.008252	N	4.687518	1.969363	-0.654784
H	2.853335	2.267080	1.265485	O	5.393655	1.802958	-1.651758
H	0.918548	1.243877	-3.191942	O	5.130672	2.203803	0.472036

PhH-Pf

Charge	0
Spin State	Singlet
Electronic Energy, BS1 (a.u.)	-959.457152
Thermal and entropic correction, BS1 (a.u.)	0.100056
Electronic Energy, BS2 (a.u.)	-959.872007
Gibbs Energy, BS2 (a.u.)	-959.771951
Number of Imaginary Frequencies	0

Molecular Geometry in Cartesian Coordinates

C	1.652555	0.000107	0.000004	C	-1.965679	-1.194547	-0.125273
C	3.760709	0.961562	-0.730890	F	0.049482	-2.364534	-0.268509
C	2.366382	-0.963958	0.731942	F	-4.004660	-0.000064	-0.000236
C	2.366353	0.963968	-0.732061	F	0.049348	2.364550	0.268687
C	4.462706	0.000049	-0.000030	F	-2.632448	-2.350081	-0.255730
C	3.760674	-0.961558	0.730804	F	-2.632636	2.349963	0.256010
C	-0.574848	-1.181756	-0.125552	H	1.828671	1.711319	-1.305260
C	0.168176	0.000020	0.000118	H	4.298097	1.711218	-1.304587
C	-0.574929	1.181798	0.125721	H	5.548975	-0.000012	-0.000103
C	-1.965774	1.194540	0.125198	H	1.828608	-1.711189	1.305206
C	-2.667037	-0.000001	-0.000263	H	4.298143	-1.711189	1.304452

S18. References

(1) D. B. G. Williams, M. Lawton, *J. Org. Chem.* **2010**, *75*, 8351–8354.

(2) C. Ammann, P. Meier, A. Merbach, *J. Magn. Reson.* **1982**, *46*, 319–321.

(3) M. Pérez-Iglesias, O. Lozano-Lavilla, J. A. Casares, *Organometallics* **2019**, *38*, 739–742.

(4) A. L. Casado, P. Espinet, *Organometallics* **1998**, *17*, 954–959.

(5) J. A. Casares, P. Espinet, J. M. Martín-Alvarez, J. M. Martínez-Ilarduya, G. Salas, *Eur. J. Inorg. Chem.* **2005**, *2*, 3825–3831.

(6) A. Doshi, A. Sundararaman, K. Venkatasubbaiah, L. N. Zakharov, A. L. Rheingold, M. Myahkostupov, P. Piotrowiak, F. Jäkle, F. Ja, *Organometallics* **2012**, *31*, 1546–1558.

(7) B. W. Skelton, A. F. Waters, A. H. White, *Aust. J. Chem.* **1991**, *44*, 1207–1215.

(8) J. Niu, H. Zhou, Z. Li, J. Xu, S. Hu, *J. Org. Chem.* **2008**, *73*, 7814–7817.

(9) G. A. Bowmaker, L. D. Brockliss, R. Whiting, *Aust. J. Chem.* **1973**, *26*, 29–42.

(10) S. Hoops, R. Gauges, C. Lee, J. Pahle, N. Simus, M. Singhal, L. Xu, P. Mendes, U. Kummer, *Bioinformatics* **2006**, *22*, 3067–3074.

(11) E. D. Kalkman, M. G. Mormino, J. F. Hartwig, *J. Am. Chem. Soc.* **2019**, *141*, 19458–19465.

(12) A. Cairncross, W. A. Sheppard, *J. Am. Chem. Soc.*, **1968**, *90*, 2186–2187.

(13) For The kinetic models we have assumed that the polifluorinated groups Rf ($C_6Cl_2F_3$) and Pf (C_6F_5) behave as electronically equivalent groups. The equivalence between Rf and Pf has been verified in our group on different occasions, see for example a) A. L. Casado, J. A. Casares, P. Espinet, *Organometallics* **1997**, *16*, 5730. b) J. A. Casares, P. Espinet, K. Soulantica, I. Pascual, A. G. Orpen, *Inorg. Chem.* **1997**, *36*, 5251–5256. c) P. Villar, M. H. Pérez-Temprano, J. A. Casares, R. Álvarez, P. Espinet, *Organometallics* **2020**, *39*, 2295–2303. And the control experiments described in S6 show that this equivalence apply in the system under study

(14) CrysAlisPro Software System, Version 1.171.33.51, 2009, Oxford Diffraction Ltd, Oxford, UK.

(15) O. V. Dolomanov, L. J. Bourhis, R. J. Gildea, J. A. K. Howard, H. Puschmann, IUCr. *J. Appl. Crystallogr*. **2009**, *42*, 339–341.

(16) Gaussian 16, Revision B.01, M. J. Frisch, G. W. Trucks, H. B. Schlegel, G. E. Scuseria, M. A. Robb, J. R. Cheeseman, G. Scalmani, V. Barone, G. A. Petersson, H. Nakatsuji, X. Li, M. Caricato, A. V. Marenich, J. Bloino, B. G. Janesko, R. Gomperts, B. Mennucci, H. P. Hratchian, J. V. Ortiz, A. F. Izmaylov, J. L. Sonnenberg, D. Williams-Young, F. Ding, F. Lipparini, F. Egidi, J. Goings, B. Peng, A. Petrone, T. Henderson, D. Ranasinghe, V. G. Zakrzewski, J. Gao, N. Rega, G. Zheng, W. Liang, M. Hada, M. Ehara, K. Toyota, R. Fukuda, J. Hasegawa, M. Ishida, T. Nakajima, Y. Honda, O. Kitao, H. Nakai, T. Vreven, K. Throssell, J. A. Montgomery, Jr., J. E. Peralta, F. Ogliaro, M. J. Bearpark, J. J. Heyd, E. N. Brothers, K. N. Kudin, V. N. Staroverov, T. A. Keith, R. Kobayashi, J. Normand, K. Raghavachari, A. P. Rendell, J. C. Burant, S. S. Iyengar, J. Tomasi, M. Cossi, J. M. Millam, M. Klene, C. Adamo, R. Cammi, J. W. Ochterski, R. L. Martin, K. Morokuma, O. Farkas, J. B. Foresman, and D. J. Fox, Gaussian, Inc., Wallingford CT, 2016.

(17) A. V. Marenich, C. J. Cramer, D. G. Truhlar, *J. Phys. Chem. B* **2009**, *113*, 6378-6396.

(18) a) C. Lee, W. Yang, R. G. Parr, *Phys. Rev. B* **1988**, *37*, 785-789. b) B. Miehlich, A. Savin, H. Stoll, H. Preuss, *Chem. Phys. Lett.* **1989**, *157*, 200-206. c) A. D. Becke, *J. Chem. Phys.* **1993**, *98*, 5648-5652.

(19) S. Grimme, J. Antony, S. Ehrlich, H. Krieg, *J. Chem. Phys.* **2010**, *132*, 154104.

(20) a) W. J. Hehre, R. Ditchfield, J. Pople, *J. Chem. Phys.* **1972**, *56*, 2257-2261. b) M. M. Francl, W. J. Pietro, W. J. Hehre, J. S. Binkley, M. S. Gordon, D. J. DeFrees, J. A. Pople, *J. Chem. Phys.* **1982**, *77*, 3654-3665.

(21) D.Andrae, U. Haeussermann, M. Dolg, H. Stoll, H. Preuss, *Theor. Chim. Acta* **1990**, *77*, 123-141.

(22) A. W. Ehlers, M. Boehme, S. Dapprich, S. A. Gobbi, A. Hoellwarth, V. Jonas, K. F. Koehler, R. Stegmann, A. Veldkamp, G. Frenking, *Chem. Phys. Lett.* **1993**, *208*, 111-114.
(23) A. Hoellwarth, M. Boehme, S. Dapprich, A. W. Ehlers, A. Gobbi, V. Jonas, K. F. Koehler, R. Stegmann, A. Veldkamp, G. Frenking, *Chem. Phys. Lett.* **1993**, *208*, 237-240.
(24) a) F. Weigend, R. Ahlrichs, *PCCP* **2005**, *7*, 3297-3305. b) F. Weigend, *PCCP* **2006**, *8*, 1057-1065.
(25) V. S. Bryantsev, M. S. Diallo, W. A. Goddard, *J. Phys. Chem. B* **2008**, *112*, 9709-9719.
(26) J. N. Harvey, M. Aschi, H. Schwarz, W. Koch, *Theor. Chem. Acc.* **1998**, *99*, 95–99.
(27) A. G. Baboul, H. B. Schlegel, *J. Chem. Phys.* **1997**, *107*, 9413.
(28) CYLview20; C. Y. Legault, Université de Sherbrooke, 2020 (http://www.cylview.org).
(29) A. V. Marenich, S. V. Jerome, C. J. Cramer, D. G. Truhlar, *J. Chem. Theory Comput.* **2012**, *8*, 2, 527–541.
